# Body composition and adipokines changes after initial treatment with darunavir-ritonavir plus either raltegravir or tenofovir disoproxil fumarate-emtricitabine: A substudy of the NEAT001/ANRS143 randomised trial

**DOI:** 10.1371/journal.pone.0209911

**Published:** 2019-01-28

**Authors:** Jose I. Bernardino, Amanda Mocroft, Cedrick Wallet, Stéphane de Wit, Christine Katlama, Peter Reiss, Patrick W. Mallon, Laura Richert, Jean-Michel Molina, Hernando Knobel, Philippe Morlat, Abdel Babiker, Anton Pozniac, Francois Raffi, Jose R. Arribas, the NEAT001/ANRS143 Trial Study Group

**Affiliations:** 1 Department of Internal Medicine, Hospital Universitario La Paz, IdiPAZ, Madrid, Spain; 2 University College London, London, United Kingdom; 3 University of Bordeaux INSERM, Bordeaux Population Health Research Center, UMR 1219, Bordeaux, France; 4 CHU Saint Pierre, Université Libre de Bruxeles, Brussels, Belgium; 5 Hôpital Pitié Salpétrière, Sorbonne Universités, UPMC Univ Paris 06, UMR_S 1136, Institut Pierre Louis d’Epidémiologie et de Santé Publique, Paris, France; 6 Department of Global Health and Division of Infectious Diseases, Academic Medical Center, University of Amsterdam, Amsterdam, The Netherlands; 7 School of Medicine, University College Dublin, Dublin, Ireland; 8 Hôpital Saint-Louis, University of Paris Diderot, Paris, INSERM U941, France; 9 Department of Infectious Diseases, Hospital del Mar, Universidad Autónoma de Barcelona, Spain; 10 Service de Medicine Interne et Maladies Infectieuses, CHU Bordeaux, Université de Bordeaux, Inserm U 1219, Bourdeaux, France; 11 MRC Clinical Trials Unit at UCL, London, United Kingdom; 12 Chelsea and Westminster hospital NHS Foundation Trust, London, United Kingdom; 13 Department of infectious diseases, CHU de Nantes and CIC 1413, INSERM, Nantes, France; Rush University, UNITED STATES

## Abstract

**Background:**

Comparison of changes in body composition, adipokines and inflammatory markers after initial therapy with a nucleos(t)ide reverse transcriptase inhibitor (N(t)RTI)- sparing or containing regimen are scarce.

**Design:**

Randomised Clinical Trial.

**Methods:**

This is the body composition substudy of NEAT 001/ANRS 143, a randomised trial comparing darunavir/ritonavir (DRV/r) plus either raltegravir (RAL) or tenofovir disoproxil fumarate/emtricitabine (TDF/FTC) in 805 ART naïve HIV-infected adults. The primary endpoint was percentage change in limb fat at week 96. Secondary endpoints were associations among these changes and metabolic markers (IL-6, insulin, leptin, adiponectin, FGF-23).

**Results:**

126 subjects (61 DRV/r + RAL and 65 DRV/r + TDF/FTC) were included. The rate of change in BMI between groups for RAL versus TDF/FTC at week 96 was 1.5% per 48-week period (p = 0.015). The rate of change in limb fat mass, trunk fat mass, total body fat and total lean mass was for RAL versus TDF/FTC at week 96 was 2.5% (p = 0.38), 7.3% ((p = 0.021), 4.9% (p = 0.061) and 1.3% (p = 0.12) respectively. Baseline insulin and leptin levels were correlated with baseline limb fat and trunk fat mass [r = 0.31 (p = 0.0043)/r = 0.28 (p = 0.0011) for limb fat, and r = 0.63 (p<0.0001)/r = 0.50(p<0.0001) for trunk fat]. After adjustment, a 10% faster increase in leptin between baseline and week 48 was associated with a more rapid increase in limb fat at week 48 (0.5% per 48 weeks, p<0.001), total body fat mass (0.6% per 48 weeks, p<0.001), and trunk fat mass (0.3% per 48 weeks, p = 0.0026).

**Conclusions:**

After week 96 a N(t)RTI sparing regimen of DRV/r + RAL produced a numerically greater percentage increase in body composition variables with only change in trunk fat mass and BMI being significant.

## Introduction

HIV-infected patients receiving currently guideline-recommended 1^st^ line antiretroviral therapy (ART) rarely develop visible alterations in fat distribution[1]. Lipoatrophy has been linked to the use of thymidine nucleoside reverse transcriptase inhibitors as a consequence of mitochondrial toxicity[2,3]. The nucleos(t)ide reverse transcriptase inhibitors (N(t)RTI) combinations currently in use (abacavir/lamivudine and tenofovir disoproxil fumarate/emtricitabine) are not considered to be related with clinical lipoatrophy although in the A5224 substudy both N(t)RTIs similarly decreased mitochondrial DNA in fat biopsies[4]. Although lipohypertrophy was initially thought to be the hallmark of exposure to protease inhibitors[5], recent trials have reported increases in visceral fat with most ART drugs including non-nucleosides reverse transcriptase inhibitors and integrase inhibitors [6,7].

Obesity and visceral adiposity are increasing concerns in people living with HIV especially in the era of modern antiretroviral therapy. In addition to traditional risk factors, HIV- and ART-related factors may also play a role.

Recent data from the ACTG 5202 and two different HIV-uninfected cohorts have shown that HIV-infected patients gained fat and lean mass in the first two years after initiating antiretroviral treatment but in the long term they lost lean body mass and gained trunk fat[8]. These body composition changes are relevant because they are associated with an increased risk of diabetes, liver disease, atherosclerosis and cardiovascular disease. Therefore it is important to investigate the contribution of different ART drugs to visceral adiposity.

Data regarding body composition changes with N(t)RTI sparing regimens in ART-naïve patients are limited. In the PROGRESS study a combination of lopinavir/ritonavir plus raltegravir (RAL) led to greater increases in peripheral fat over 96 weeks compared with lopinavir/ritonavir plus tenofovir disoproxil fumarate/emtricitabine (TDF/FTC)[9]. The RADAR study comparing a combination of darunavir/ritonavir (DRV/r) plus RAL with DRV/r plus TDF/FTC found no evidence of significant differences in total body fat or lean fat mass over 48 weeks[10].

Chronic inflammation and immune activation associated with HIV infection could modulate visceral adiposity changes seen in HIV-infected individuals under long-term antiretroviral therapy[11,12]. There is some evidence of an association between chronic inflammation and increases in visceral adipose tissue in patients with lipodystrophy[13]. Few studies have looked into the associations between antiretroviral drugs, body composition changes and adipokines and some have raised the hypothesis that increases in visceral adipose tissue could be associated with higher leptin and lower adiponectin levels[7,14].

NEAT 001/ANRS 143 evaluated the virologic efficacy and tolerability of ritonavir-boosted darunavir in combination with tenofovir disoproxil fumarate (TDF)-emtricitabine or raltegravir in treatment naïve HIV-infected adults in Europe[15]. In the current report, we examined the body composition effect, as well as adipokines and metabolic biomarkers changes over 96 weeks after starting ART.

## Patients and methods

NEAT 001/ANRS 143 was a randomised, open-label, 96 weeks, non-inferiority trial of ART-naïve conducted in 78 clinical sites in 15 European countries between August 2010 and October 2013. The present substudy was conducted over 19 clinical sites. Inclusion criteria were: HIV RNA greater than 1000 copies per mL and CD4 cell count under 500 cells per μL in ART-naïve participants and no evidence of major International Antiviral Society-USA resistance mutations. Exclusion criteria were: receiving treatment for mycobacteriosis or malignant disease, tested positive for HBsAg, pregnancy, relevant laboratory abnormalities and an estimated creatinine clearance less than 60 mL/min. Additional exclusion criteria for the bone mineral density and body composition sub-studies were: treatment for osteoporosis and concomitant use of steroids or oestrogen replacement therapy at screening [15,16].

Any patient enrolling NEAT001/ANRS143 study in a site participating in the bone mineral density substudy and fulfilling the inclusion criteria was suitable for this pre-planned analysis. Randomisation was performed at the same time for the parent study and after having consented for the bone mineral density substudy. Briefly, patients were enrolled into the substudy before being randomised in the core trial to receive 800 mg darunavir and 100 mg ritonavir once daily plus 400 mg raltegravir twice daily [N(t)RTI-sparing regimen] or 800 mg darunavir and 100 mg ritonavir plus tenofovir disoproxil fumarate/emtricitabine in a 300 mg and 200 mg fixed-dose combination. The primary endpoint of this body composition analysis was the mean percentage change of limb fat mass between baseline (randomisation) and week 96. The secondary endpoints included: mean percentage change of trunk fat mass, total body fat and total body lean mass and body mass index (BMI) at 96 weeks, predictors of body composition changes and associations between adipokines and inflammatory markers with body composition changes. The study protocol was approved by the Ethics Committee Regional de Madrid in accordance with the principles of the Declaration of Helsinki. The study was also approved by individual ethics committees in the following centres: Ethikkomission der Stadt Wien, Universitair Ziekenhuis Antwerpen, Region Hovedstaden De Videnskabseliske Komiteer, CPP Ile de France VI Groupe Hospitalier Pitié Salpatreire, Hanover School of Medicine, Hellenic Republic Minister of Health and Welfare National ethic committee, Medical research council Ethics committee in Hungary, Mater Private Hospital Research Ethics Committee, Centrum Medyczne Ksztakcenia podyplomowego Komisja Biothyczna, Comissao de Etica para a Investigacao Clinica, Karolinska university hospital regional etikprovningsnamnden i Stockholm, NHS leeds East Research Ethics Committee and Medisch Ethische Toetsingscommissie Academisch Medisch Centrum. All trial participants gave specific written informed consent for the bone mineral density substudy and the parent study at the same time and therefore were included in this body composition substudy prior to randomised treatment allocation. (ClinicalTrials.gov registration number NCT01066962).

### Study procedures

Whole body dual energy X-ray absorptiometry (DXA) scans using either Hologic (Hologic Inc., Bedford, Massachusetts, USA) or GE Lunar (GE Healthcare Lunar, Madison, Wisconsin, USA) devices were performed at baseline, weeks 48 and 96. Total limb fat (upper plus lower extremities fat), trunk fat, total body lean mass and total body fat mass were quantified using standardized scanning protocols and locally calibrated based on each manufacturer’s specifications. Technicians -blinded to study clinical information- were instructed to use the same device on the same patient during the trial without central reading.

Adipokines and markers of inflammation were performed in the department of Laboratory Medicine at Hospital Universitario La Paz, Madrid on batched serum samples stored at -80°C from weeks 0 and 48. The following markers were analysed: IL-6; IL-1β; TNF-α, Insulin, Leptin, Adiponectin and Fibroblast Growth Factor-23 (FGF-23) with a multiplex magnetic bead panel assay (HBNMAG-51 Merck Millipore) using a Luminex 200 analyzer.

### Statistical analysis

Sample size was calculated for bone mineral density changes. We calculated that 64 patients per arm would provide 80% power to detect a treatment difference of 2.5% in BMD in lumbar spine assuming a standard deviation of 5 and a 2-sided t-test with alpha of 0.05. Assuming a 10% drop out rate a total of 142 patients would be needed. All analysis were performed in an intent to treat approach (participants who changed treatment during the study were analysed in the same groups as at randomisation).

Descriptive statistics were used to summarise patient characteristics in each treatment arm and were compared using chi-squared tests, unpaired t-tests or non-parametric Wilcoxon tests. Percentage changes in body composition variables from baseline to week 48 and baseline to week 96 were assessed and compared between treatment arms using mixed models with a random intercept and allowing for repeated observations per patient. This method estimates the difference in the rate of change between treatment arms and the difference was standardised per 48-week period. Multivariable models were adjusted for factors that were associated with changes in univariable analyses with p<0.1. Mixed models were also used to assess whether the percentage change in biomarkers were associated with changes in body composition from baseline to week 48. Any adipokines or inflammatory markers that were significant in univariable analyses (p<0.1) were included in the multivariable analysis. Sensitivity analysis was planned using medians and non-parametric tests, and also including laboratory variables as binary (>5% change).

Statistical analyses were performed using SAS (Statistical Analysis Software; Cary, NC, USA) Version 9.3. All tests of significance were 2-sided.

## Results

**Fig 1:**
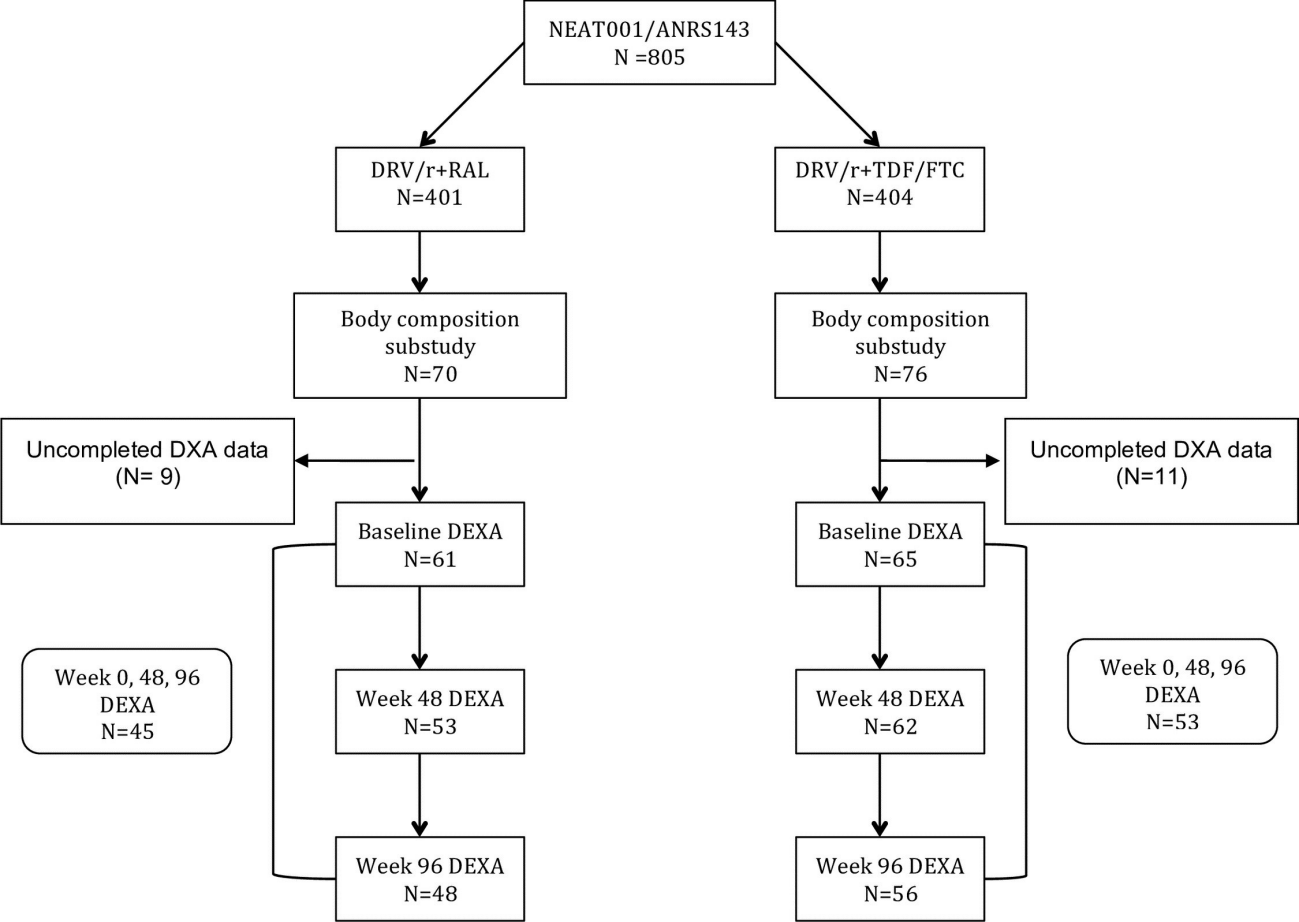
Flow chart of NEAT001/ANRS143 body composition substudy.

A total of 146 subjects were included and randomized. The intention-to-treat-exposed population comprised 126 subjects with at least one DXA performed, 61 subjects in the DRV/r + RAL group and 65 subjects in the DRV/r + TDF/FTC, of whom 48 and 56, respectively, completed week 96 (Fig 1). Baseline characteristics were well balanced between groups (Table 1) and did not differ from those of the core trial (data not shown). Participants were 90.5% male and 82% Caucasian. The median age was 40 years (IQR 31–46); median CD4 count was 338 cells/mm^3^ (IQR 279–407); median viral load was 4.7 log_10_ copies/mL (IQR 4.3–5.1); median body mass index (BMI) was 23.2 Kg/m^2^ (IQR 21.4–26.2); and median waist to hip ratio was 0.91 (IQR 0.86–0.98).

There were no statistical differences between groups in body composition at randomization except for a median higher total body fat mass in the DRV/r + TDF/FTC group [17.1 kg (IQR 11–19.8) vs. 14.3 kg (IQR 9.8–22.9); p = 0.014] (Table 1).

**Table 1:** Baseline characteristics.

	DRV/r + RAL (N = 61)	DRV/r + TDF/FTC (N = 65)	Total (N = 126)
Gender (Male): n (%)	53 (86.9)	61 (93.9)	114 (90.5)
Age, years: Median (IQR)	39 (31–45)	40 (32–46)	40 (31–46)
Ethnicity: n (%)			
Caucasian	50 (82)	53 (81.5)	103 (81.7)
Other	11 (18)	12 (18.5)	23 (18.3)
Current Smoking: n (%)	25 (41%)	31 (47.7%)	56 (44.4%)
Current Alcohol use: n (%)	3 (4.9)	2 (3.1)	5 (4%)
Baseline HIV-1 RNA, log _10_ copies/ml: Median (IQR)	4.8 (4.3–5.2)	4.7 (4.4–5.0)	4.7 (4.3–5.1)
Baseline CD4+, cells/mm^3^: Median (IQR)	347 (280–393)	331 (278–414)	338 (279–407)
CD4 + nadir, cells/mm^3^: Median (IQR)	327 (236–370)	327 (268–374)	327 (260–374)
Weight, Kg: Median (IQR)	72 (64–80)	72 (67–81)	72 (65–80)
BMI, Kg/ m^2^: Median (IQR)	22.4 (20.9–26.1)	23.4 (21.7–26.3)	23.2 (21.4–26.2)
Waist, cm: Median (IQR)	82 (77–89)	84 (79–93)	84 (78–91)
Hip, cm: Median (IQR)	92 (86–99)	94 (87–99)	93 (86–99)
Waist: hip ratio: Median (IQR)	0.92 (0.85–0.97)	0.91 (0.87–0.98)	0.91 (0.86–0.98)
Total body fat mass, Kg: Median (IQR)	14.3 (9.8–22.9)	17.1 (11–19.8)	15.4 (10.7–21.9)
Limb fat, Kg: Median (IQR)	6.7 (4.7–10.3)	6.4 (5.2–9.1)	6.7 (4.7–9.7)
Trunk fat, Kg: Median (IQR)	7.8 (4.6–11.9)	8.8 (5.4–11.6)	8.5 (4.5–11.7)
Lean body mass, Kg: Median (IQR)	49.2 (45.1–52.5)	50.5 (46.8–54.4)	50.1 (46.1–54.2)
Walking hours/week: Median (IQR)	4 (2–6)	4 (2–7)	4 (2–7)
IL-6, pg/mL: Mean (95%CI)	1.2 (0.0–2.4)	1.4 (0.6–2.2)	1.3 (0.5–2.1)
IL-1β, pg/dL: Mean (95%CI)	0.4 (0.3–0.5)	0.4 (0.3–0.5)	0.4 (0.3–0.5)
TNF-α, pg/dL: Mean (95%CI)	4.1 (2.7–5.5)	4.0 (3.6–4.4)	4.0 (3.4–4.6)
Insulin, μU/ml: Mean (95%CI)	6.3 (2.6–10)	5.1 (3.9–6.3)	5.6 (3.8–7.4)
Leptin, ng/mL: Mean (95%CI)	5.58 (3.01–8.15)	4.45 (2.67–6.21)	4.96 (3.45–6.48)
Adiponectin, mcg/mL: Mean (95%CI)	23.9 (19–28.8)	15.8 (13.3–18.3)	19.5 (16.8–22.2)
FGF-23, pg/mL: Mean (95%CI)	36.5 (32–41)	38.8 (35.1–42.5)	37.7 (34.8–40.6)
HOMA-IR: Mean (95% CI)	1.6 (0.97–2.23)	1.97 (0.6–3.34)	1.30 (0.99–1.61)

DRV/r, darunavir/ritonavir; RAL, raltegravir; TDF/FTC, tenofovir disoproxil fumarate/emtricitabine, BMI: body mass index; IL, interleukin; TNF, tumour necrosis factor; FGF, fibroblast growing factor; HOMA-IR, homeostatic model assessment-insulin resistance

### Changes in body composition

There was a small but significant increase in BMI in both arms from baseline to week 48 and baseline to week 96. The rate of change in the DRV/r + TDF/FTC group was 2.2% per 48 week period (95% CI 0.6–3.7, p = 0.0074) at week 48 and 1.0% per 48 week period (95% CI 0.2–0.8, p = 0.023). The rate of change was higher in the DRV/r + RAL group at both 48 weeks (difference 2.1% per 48 week period; 95% CI -0.2–4.2;p = 0.078) and 96 weeks (difference 1.5% per 48 week period; 95% CI 0.3–2.6;p = 0.015).

**Fig 2:**
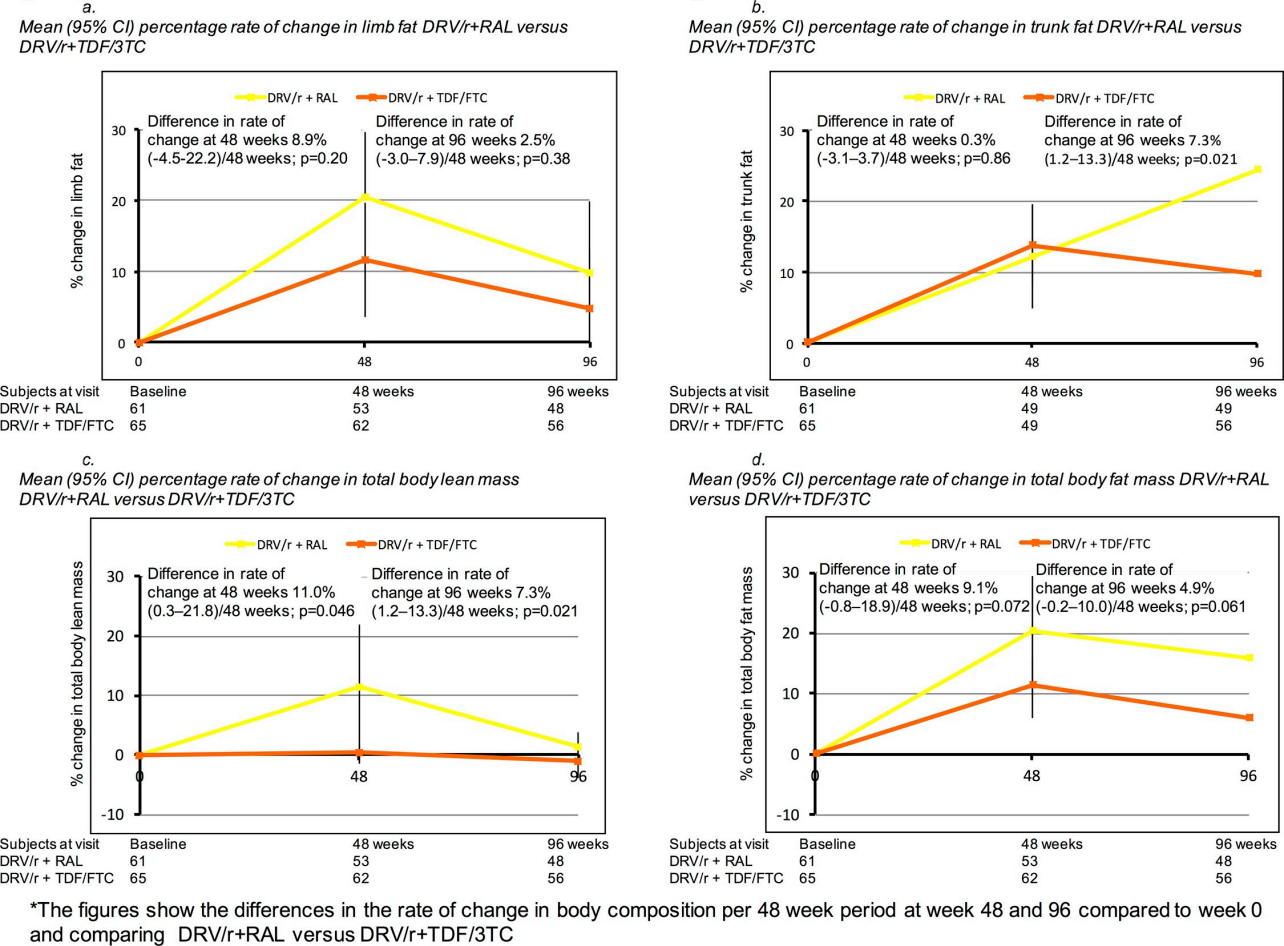
Changes in body composition variables over 96 weeks. (A) Mean (95% CI) percentage rate of change in limb fat. (B) Mean (95% CI) percentage rate of change in trunk fat. (C) Mean (95%CI) percentage rate of change in total body lean mass. (D) Mean (95%CI) percentage rate of change in total body fat mass. Abbreviations: DRV/r, Darunavir/ritonavir; RAL, Raltegravir; TDF/FTC, Tenofovir disoproxil fumarate/Emtricitabine; HIV, Human immunodeficiency virus; CI, confidence interval.

Of the 126 patients included in the current analysis, 115 (53 in the DRV/r + RAL arm and 62 in the DRV/R + TDF/FTC arm) had a DXA performed at 48 week and 114 (48 in the DRV/r + RAL arm and 56 in the DRV/R + TDF/FTC arm) at 96 week. Body composition variables increased from baseline to week 48 and baseline to week 96 in the combined study population and in both treatment groups, with higher increases in the DRV/r +RAL groups (Fig 2), particularly for limb fat mass, trunk mass, total body lean mass and total body fat. For example, the rate of change in limb fat mass was higher at 48 weeks for DRV/r + RAL versus DRV/r + TDF/FTC (8.9% per 48 week period; 95% CI -4.5–22.2, p = 0.20) and at 96 weeks (2.5% per 48 week period; 95% CI -3.0–7.8, p = 0.38), although not statistically significant. The largest differences in the rate of change comparing DRV/r + RAL versus DRV/r + TDF/FTC were seen for trunk fat at week 96 (7.3% per 48 week period, 95% CI 1.2–13.3, p = 0.021), total body lean mass at 48 weeks (11.0% per 48 week period; 95% CI 0.3–21.8%, p = 0.046) and in total body mass at 48 weeks (9.1% per 48 week period, 95% CI -0.8–18.9%, p = 0.072) and 96 weeks (4.9% per 48 week period; 95% CI -0.2–10.0, p = 0.061).

Although globally there was a trend for increases in fat, there were some participants with fat loss. Indeed, 33 participants (26%) had a limb fat loss ≥ 10% and 18 participants (14.4%) lost ≥ 20% of limb fat, but differences in proportions with limb fat loss ≥ 10% or ≥ 20% were not statistically significant between treatment arms (p = 0.12 and p = 0.28 respectively).

### Associations between adipokines/inflammatory markers and body composition

We measured at least one of the inflammatory markers in 87 patients. Absolute baseline values for adipokines and inflammatory biomarkers were similar between treatment groups except for adiponectin [15.8 mcg/mL in DRV/r + TDF/FTC vs. 23.9 mcg/mL in DRV/r + RAL; mean difference -8.1 (95% CI -13.5, -2.7); p = 0.004]. Correlations between baseline adipokines, inflammatory markers and body composition variables (limb, trunk and total body fat) were weak except for leptin r = 0.63 (p<0.0001), r = 0.50 (p<0.0001) and r = 0.63 (p<0.0001) respectively. Other significant correlations were between baseline insulin and baseline limb fat/trunk fat (r = 0.31, P = 0.004/r = 0.28, P = 0.012) and baseline adiponectin with baseline limb fat and total body fat (r = 0.4, P<0.001/r = 0.27, P = 0.01). Insulin resistance at baseline, measured by the homeostatic model assessment (HOMA-IR), was also significantly correlated with limb, trunk and total body fat mass (Table 2).

**Table 2:** Correlation between inflammatory markers and body composition parameters.

	**IL-6 (n = 87)**		**Insulin (n = 84)**		**Leptin (n = 87)**		**Adiponectin (n = 86)**		**FGF-23 (n = 85)**		**HOMA-IR (n = 84)**	
**Baseline**	R	P	R	P	R	P	R	P	R	P	R	P
Limb fat	-0.03	0.78	0.31	0.004	0.63	<0.001	0.40	<0.001	0.17	0.12	0.32	0.0035
Trunk fat	-0.07	0.48	0.28	0.012	0.50	<0.001	0.08	0.44	0.09	0.39	0.25	0.023
Lean body mass	0.07	0.48	-0.13	0.25	-0.25	0.02	-0.14	0.20	-0.07	0.52	-0.09	0.41
Total body fat mass	-0.06	0.59	0.32	0.0028	0.63	<0.001	0.27	0.01	0.15	0.17	0.31	0.0040
**Percentage change at week 48**	**IL-6 (n = 69)**		**Insulin (n = 64)**		**Leptin (n = 64)**		**Adiponectin (n = 68)**		**FGF-23 (n = 64)**		**HOMA-IR (n = 64)**	
Limb fat	0.16	0.2	0.46	<0.001	0.70	<0.001	-0.05	0.68	-0.00	0.99	0.31	0.014
Trunk fat	0.22	0.07	-0.23	0.072	0.27	0.03	-0.07	0.57	0.01	0.91	0.11	0.42
Lean body mass	-0.16	0.18	-0.03	0.82	-0.08	0.51	0.03	0.83	-0.19	0.14	-0.04	0.76
Total body fat mass	-0.15	0.23	0.46	<0.001	0.60	<0.001	-0.08	0.50	-0.04	0.75	0.33	0.0084

IL, interleukin; TNF, tumour necrosis factor; FGF, fibroblast growth factor 23; HOMA-IR, homeostatic model assessment-insulin resistance

We then explored if changes in these markers between baseline and week 48 were related with percentage changes in body composition variables at week 48. Again the strongest correlations were between changes in insulin, leptin, and HOMA-IR with limb fat and total body fat mass (Table 2).

We used multivariable mixed model to assess the rate of change in adipokines and inflammatory markers between randomisation and week 48 in relation to percentage change in body composition parameters (Table 3), after adjustment for treatment arm, history of fractures at baseline, nadir CD4, CD4 count and viral load at randomisation. In univariable analyses, a faster increase in leptin was associated with an increase in limb fat, trunk fat or total body fat mass at week 48, while a faster increase in HOMA-IR was associated with an increase in limb fat mass and total body fat mass. There was no evidence that IL-6, adinopectin, FGF-23, IL1-beta and TNF-alpha were associated with changes in body composition parameters in univariable analyses (p>0.1), and these were therefore not included in multivariable models.

**Table 3:** Associations between percentage change in biomarkers and percentage change in body composition from randomisation to 48 weeks.

	Univariate			Multivariate		
	Estimate	95% CI	P-value	Adjusted^b^ Estimate	95% CI	P-value
**Limb fat mass**						
Insulin	0.53	0.39, 0.67	<0.001	0.09	-0.05, 0–23	0.2
Leptin	0.61	0.53, 0.69	<0.001	0.52	0.42, 0.62	<0.001
HOMA-IR	0.34	0.19, 0.49	<0.001	0.17	0.05, 0.29	0.0065
RAL + DRV/r vs. TDF/FTC + DRV/r	8.86	-4.47, 22.19	0.2	2–30	-4.95, 9.55	0.54
**Trunk fat mass**						
Insulin	-0.32	-0.50, -0.14	0.0012	-0.48	-0.67, -0.29	<0–001
Leptin	0.32	0.17, 0.47	<0.001	0.25	0.10, 0.40	0.0026
HOMA-IR	0.10	-0.03, 0.23	0.14	0.25	0.12, 0.38	<0.001
RAL + DRV/r vs. TDF/FTC + DRV/r	-1.60	-9.54, 6.34	0.69	-4.90	-12.90, 3.10	0.23
**Total body fat mass**						
Insulin	0.50	0.35, 0.65	<0.001	0.03	-0.12, 0.18	0.7
Leptin	0.64	0.55, 0.73	<0.001	0.59	0.49, 0.69	<0.001
HOMA-IR	0.32	0.17, 0.47	<0.001	0.17	0.04, 0.30	0.011
RAL + DRV/r vs. TDF/FTC + DRV/r	9.05	-0.75, 18.85	0.072	1.23	-6.49, 8.95	0.76

The estimates show the association between biomarkers and measures of body composition from mixed models which estimate the rate of percentage change in body composition (limb fat mass, trunk fat mass or total body fat mass) per 48 week period associated with a 10% change in the change in biomarkers over the same time period.

^b^Adjusted additionally for fractures at baseline, nadir CD4+, CD4+ count and viral load at randomisation. DRV/r +RAL: darunavir/ritonavir plus raltegravir. DRV/r + TDF/FTC: darunavir/ritonavir plus tenofovir/Emtricitabin. HOMA-IR, homeostatic model assessment-insulin resistance

After adjustment, on average, a 10% faster increase in leptin between baseline and week 48 was associated with a more rapid increase in limb fat at week 48 (0.5% per 48 weeks, 95% CI 0.4, 0.6; p<0.001), a 0.6% per 48 weeks faster increase in total body fat mass (95% CI 0,5, 0.7; p<0.001) and a 0.3% per 48 weeks faster increase in trunk fat mass (95% CI 0.1,0.4; p = 0.0026). A faster increase in HOMA-IR was also associated with greater increases in limb fat mass, trunk fat or total body fat mass (Table 3).

## Discussion

In the present analysis we report a statistically significant greater increase in body mass index and trunk fat in the DRV/r + RAL at week 96. We found that gains in limb and total body fat peaked at 48 weeks in both arms and subsequently decreased except for trunk fat mass in the DRV/r + RAL. These changes in body composition variables were related with leptin changes during the first 48 weeks.

There are limited data on the effect of integrase inhibitors containing regimens on body composition in antiretroviral naïve patients. The STARTMRK study randomized HIV-infected patients to efavirenz vs. raltegravir both with TDF/FTC. In a convenience sample of 86 participants at 48 weeks and 75 participants at 96 weeks, percentage changes in appendicular and trunk fat were similar between arms [6]. Those allocated to the TDF/FTC + RAL arm had a 19% increase in trunk fat at week 48 and 21.6% at 96 weeks. In the ACTG 5260s study, central and peripheral fat gains were similar in patients treated with raltegravir or the protease inhibitors atazanavir or darunavir all in combination with TDF/FTC[7]. Those allocated to the TDF/FTC + RAL arm had a 19.4% increase in trunk fat at week 96. Our study gave the opportunity to compare a NtRTI-containing and a NtRTI-sparing regimen, with a backbone of ritonavir-boosted-darunavir.

We did not find significant differences in body changes from baseline between the RAL and the TDF/FTC arm at week 96 except for trunk fat mass that was significantly higher in the N(t)RTI sparing arm. In ACTG 5260s percentage change in limb fat and trunk fat at 96 weeks in the DRV/r plus TDF/FTC arm were greater than in our study (14.2%, 20.8% vs. 4.9%, 9.8%). These differences could be partially explained by patients’ baseline characteristics: slightly older population (median age of 40 years vs. 36 years) and Caucasian origin in a majority of participants (89% vs. 44%) in our study. In the STARTMRK baseline CD4 count was lower than in our study (median of 212 cells/mm^3^ vs. 338 cells/mm^3^) and the return to health phenomena could have played a more determinant role in the results. Differential effects on fat redistribution across studies could also be explained by genetic determinants. This has been suggested in the recent SECOND-LINE metabolic study where African participants were more prone to lose fat in comparison with Asian participants[17].

In the NEAT 001/ANRS 143 study participants randomized to the RAL group tended to have higher increases in limb, trunk and total body fat mass as well as higher increases in BMI over 96 weeks than participants randomized to the TDF/FTC group. Two other studies have reported body composition changes associated to initial treatment with a N(t)RTI sparing regimen consisting of an integrase strand-transfer inhibitor plus a ritonavir-boosted protease inhibitor. In the PROGRESS study increases in peripheral and trunk fat were greater in patients treated with lopinavir/ritonavir plus raltegravir arm compared to patients treated with lopinavir/ritonavir plus tenofovir disoproxil fumarate/emtricitabine, although this difference was only significant for peripheral fat, both in arms and legs [9]. In the RADAR study[10], with a smaller sample size and only data at 48 weeks, there was a non significant greater increase in total body fat in the DRV/r + RAL arm compared to DRV/r + TDF/FTC. Data on trunk fat were not provided. These two studies had a virologic primary endpoint and therefore may have been underpowered to detect differences in body composition variables. All these studies focusing on the effect of raltegravir-based regimens in naïve HIV-infected patients on body composition variables show a differential effect when RAL is used with or without TDF/FTC. The present substudy was specifically designed to find differences in bone mineral density and body composition variables between treatment arms. Combined results of NEAT 001/ANRS143, PROGRESS and RADAR suggest that the greater increase in body composition fat variables observed in patients receiving N(t)RTI sparing regimens could be related to the avoidance of a deleterious effect on fat of TDF/FTC. It has been reported that TDF/FTC decreases both mitochondrial DNA in fat biopsies and the activity of mitochondrial oxidative phosphorylation enzymes[4].

Data about the relationship between insulin, adipokines, and body composition changes in HIV-infected patients are limited. In the ACTG 5260 higher levels of inflammatory markers at baseline were associated with increases in peripheral fat and lean body mass but not central adiposity[7]. In this trial, lower baseline leptin level, higher adiponectin and higher viral load were associated with greater gains in visceral adipose tissue, although the association with adiponectin was marginal. In our study we found, as expected, a positive correlation between baseline insulin and leptin levels and limb, trunk and total body fat. After adjustment, a 10% increase in leptin from baseline was correlated with a highly significant increase in limb fat mass at week 48 independently of treatment arm. Associations between circulating adipokines and visceral fat are not always robust[18]. The discrepancies between NEAT 001/ANRS 143 and ACTG 5260 may be due to participants’ characteristics. In NEAT 001/ANRS 143 participants were mostly Caucasians and had lower BMI, lower limb fat, trunk fat and lean body mass at baseline. In ACTG 5260 percentage of moderate physical exercise was roughly 80% in the three arms. We cannot exclude a differential contribution of physical exercise among these studies although in our study physical exercise was not associated with percentage change n body composition variables.

Other studies have reported an association between inflammation and visceral/subcutaneous adipose tissue [19,20]. Recent findings suggest that chronic inflammation could partially influence long-term body composition changes. In a study of malnourished African patients, a one log reduction in CRP was associated with almost 600 grams increase in fat-free mass after 6 weeks of starting ART[21]. We did not find associations between IL-6 and body composition changes in our study and this could be explained by the smaller sample size, the good health at baseline and the recent HIV diagnosis (median HIV duration 1.8 years) of NEAT 001/ANRS 143 participants.

Our study has several limitations. Participants were mainly Caucasian males and therefore the results might not be generalizable. DXA measures were locally but not centrally calibrated and there was no central reading. We did not have accurate data on visceral adiposity because we did not perform CT scans, however in the recent ACTG 5260s trial there was a good correlation between visceral adiposity measured by CT scan and trunk fat measured by DXA so we consider this limitation should not greatly influence our results. Sample size was calculated for bone mineral density changes and therefore we could have had insufficient power to detect mild differences in the body composition variables between arms. Another limitation is that inflammatory markers and adipokines were only measured in a limited subset of participants.

In summary, after 96 weeks a N(t)RTI sparing regimen of DRV/r + RAL produced a numerically greater percentage increase in body composition variables, with change in trunk fat mass and BMI being significant, compared to the TDF/FTC + DRV/r regimen. Changes in leptin levels during the first 48 weeks were correlated with changes in limb, trunk and total body fat independently of treatment arm. Future adequately powered studies with longer follow-up are needed to better understand the interplay between modern antiretroviral drugs and visceral adiposity.

## Supporting information

S1 Table Individual authors and affiliations of NEAT001/ANRS143 Trial Study Group. (PDF)

S1 File Complete NEAT001/ANRS143 protocol and substudies. (PDF)

S1 Fig Consort checklist. (DOC)
