# Supplementary material for: Body composition and adipokines changes after initial treatment with darunavir-ritonavir plus either raltegravir or tenofovir disoproxil fumarate-emtricitabine: A substudy of the NEAT001/ANRS143 randomised trial
Source: PLoS One. 2019 Jan 28;14(1):e0209911. doi: 10.1371/journal.pone.0209911 (PMC6349314; doi:10.1371/journal.pone.0209911)
Supplement: S1 File — (PDF) [file pone.0209911.s002.pdf]

NEAT Protocol 001 / ANRS 143

ACRONYM: NEAT 001

An open-label randomised two-year trial comparing two first-line regimens in HIV-infected antiretroviral naïve subjects:
darunavir/r + tenofovir/emtricitabine vs. darunavir/r + raltegravir

Clinicaltrials.gov identifier: NCT01066962
EUDRACT #: 2009-015113-44
CTA #:

Protocol version number 6.0
including amendments #1, #2, #3, #4 and #5

Protocol date 17/04/2013

Chair of the Trial Development Team: François Raffi
Centre Hospitalier Universitaire de Nantes,
Dep.of Infectious Diseases HIV Clinical Research Unit,
Nantes, France
phone: + 33 2 40 08 33 72
fax: + 33 2 40 08 31 81
e-mail: francois.raffi@chu-nantes.fr

Protocol authors:
François Raffi, University Hospital Nantes, France
Clotilde Allavena, University Hospital Nantes, France
Abdel Babiker, MRC, London, UK
Laura Richert, Inserm U897, Bordeaux, France
on behalf of the Trial Development Team

Legal sponsor:
Inserm – ANRS (Institut national de la santé et de la recherche médicale - Agence nationale de recherche sur le sida et les hépatites virales), 101 rue de Tolbiac, 75013 Paris, France

CONTACT DETAILS

- **Medical expert** *(or Clinical Principal Investigator or Coordinating Investigator)*
  This should cover emergency advice. Name, title, address, telephone
- **Regional CTU**
- **National coordinating investigator**

Contact details are country-specific. Names, telephone and fax numbers to be inserted by regional CTU

- **Chairs of the Trial Management Team**

**Chair**	François Raffi	Centre Hospitalier Universitaire de Nantes, Dep.of Infectious Diseases HIV Clinical Research Unit, Nantes, France
**Co-chair**	Anton Pozniak	St Stephen's AIDS trust, Dep. of HIV, London, UK

- **Clinical laboratories, medical and technical departments, institutions**
  See working groups (appendix 18)

RANDOMISATIONS

Tel: 00000000 (Mon - Fri, 09:00 – 17:00)
Fax: 0000000

SAE NOTIFICATION

Within 24 hours of becoming aware of an SAE, please send a completed SAE form to the NEAT regional CTU.

Fax: 00 0000 0000

Randomisation and SAE notification numbers are specific to each regional CTU. Telephone and fax numbers to be inserted by regional CTU

PROTOCOL SIGNATURE PAGE

**Protocol details**	
Study title:	An open-label randomised two-year trial comparing two first-line regimens in HIV-infected antiretroviral naïve subjects: darunavir/r + tenofovir/emtricitabine vs. darunavir/r + raltegravir
Protocol number:	NEAT 001/ANRS 143
Version:	
Last modified on:	
Status:	

The signatures below constitute approval of this protocol including appendices and provides assurance that this study is conducted according to the stipulations contained within this document, including the statements of confidentiality, provision of indemnity for clinical staff employed by the institution, and good clinical practice. The signature of Jean-François Delfraissy on behalf of the legal sponsor Inserm - ANRS provide assurance of the Sponsors' approval of the protocol including statements about provision of a policy to provide cover for clinical trial liability other than clinical negligence and negligence during the conduct of the clinical trial.

Signed ________________ Date: __________
Legal sponsor representative by delegation

Signed ________________ Date: __________
Chair of the Trial Development Team

Signed ________________ Date: __________
Trial Statistician

Signed ________________ Date: __________
National coordinating investigator

CONTENTS

Contact Details 2
List of Tables 8
List of Figures 8
Abbreviations and Glossary 9
General information 12
1. Summary 13
2. Background 20
2.1 Introduction 20
2.2 Rationale for the NEAT 001/ANRS 143 trial 20
2.2.1 Standard first-line antiretroviral regimens 20
2.2.2 Novel treatment strategies 22
2.2.3 Comparator groups of the NEAT 001/ANRS 143 trial 25
2.3 Investigational products 25
2.3.1 Tenofovir disoproxil difumarate and emtricitabine 26
2.3.2 Darunavir/ritonavir 26
2.3.3 Raltegravir 27
2.4 Objectives 27
2.4.1 Primary objective 27
2.4.2 Secondary objectives 27
2.5 Methodological rationale 28
2.6 Risks and benefits 28
2.6.1 Darunavir/ritonavir + tenofovir/emtricitabine arm 28
2.6.2 Darunavir/ritonavir + raltegravir arm 29
2.7 Overview of ancillary studies and sub-studies 29
2.7.1 Ancillary studies 29
2.7.2 Sub-studies 30
3. Study design 30
3.1 Primary endpoint 30
3.2 Secondary endpoints 31
3.3 Other endpoints 31
3.4 Trial calendar 32
4. Study population 32
4.1 Number and type of subjects 32
4.2 Inclusion criteria 32
4.3 Non-inclusion criteria 33
4.4 Enrolment recommendations 34
5. Investigational products 35
5.1 Introduction 35
5.2 Name and description of the investigational medicinal products 35
5.2.1 Darunavir/r + tenofovir/emtricitabine arm 35
5.2.2 Darunavir/r + raltegravir arm 35
5.3 Provision of the trial drugs 36
5.4 Packaging and labelling 36
5.5 Handling and storage 36
5.6 Responsibilities 37

5.7 Accountability ... 37

6. Concomitant medication ... 38
6.1 Hepatitis C treatment ... 38
6.2 Not permitted medications / Precautions ... 38
6.2.1 HIV medication ... 38
6.2.2 Tenofovir/emtricitabine containing arm ... 38
6.2.3 Darunavir/ritonavir containing arms ... 39
6.2.4 Raltegravir containing arm ... 44
6.3 Data on concomitant medication ... 45

7. Study conduct ... 46
7.1 Signature of informed consent ... 46
7.2 Screening visit (W-6 to W-1) ... 46
7.3 Randomisation and treatment allocation ... 47
7.4 Baseline visit (W00) ... 48
7.5 Follow-up visits ... 50
7.5.1 Visit W02 ... 50
7.5.2 Visit W04 ... 51
7.5.3 Visit W08 ... 52
7.5.4 Visit W12 ... 53
7.5.5 Visit W18 ... 54
7.5.6 Visit W24 ... 55
7.5.7 Visit W32 ... 56
7.5.8 Visit W48 ... 57
7.5.9 Visit W64 ... 58
7.5.10 Visit W80 ... 59
7.5.11 Visit W96 ... 60
7.6 Visits after W96 ... 61
7.7 Assessment of safety ... 62
7.8 Assessment of efficacy ... 62
7.9 Assessment of treatments ... 62
7.9.1 Antiretroviral treatment ... 62
7.9.2 Concomitant treatment ... 63
7.10 Assessment of compliance and adherence ... 63
7.11 Other assessments ... 63
7.12 Study regimen termination ... 64
7.12.1 Management in the case of insufficient virologic response ... 65
7.12.2 Management in the case of protocol-defined virologic failure ... 65
7.12.3 Management in the case of disease progression ... 66
7.12.4 Management in the case of treatment limiting adverse events ... 66
7.12.5 Second line antiretroviral regimens ... 67
7.13 Management in the case of pregnancy ... 67
7.14 Management in the case of suspicion of Immune Reconstitution Syndrome ... 67
7.15 Patient transfers ... 67
7.16 Discontinuation and withdrawal ... 67
7.16.1 Premature discontinuation from the study ... 67
7.16.2 Withdrawal of consent ... 68
7.16.3 Loss to follow-up ... 68
7.17 Data collection ... 68
7.18 Trial close out ... 69

8. Safety reporting ... 69
8.1 Adverse Events ... 70
8.1.1 Definitions ... 70
8.1.2 Clarifications and Exceptions ... 70

8.2 Serious Adverse Events 71
8.2.1 Definitions 71
8.2.2 Suspected unexpected serious adverse reactions (SUSAR) 72
8.2.3 Trial Specific Events requiring Expedited SAE Notification and Reporting 72
8.3 Investigator responsibilities 72
8.3.1 Severity and causality of adverse events 72
8.3.2 Pregnancy notifications 75
8.4 Sponsor's responsibilities 75

9. Statistical considerations 75
9.1 Sample size 75
9.2 Analysis of the primary endpoint 76
9.3 Interim monitoring and analyses 77
9.4 Analyses of other data 78

10. Data management 78
10.1 Data collection 78
10.2 Data not to be recorded in the CRF 79
10.3 Data quality control 79
10.3.1 Data monitoring at the regional CTUs 79
10.3.2 On site monitoring 79
10.3.3 Direct access to source data 79
10.4 Further data management 79

11. Ethical considerations 80
11.1 Ethical approval 80
11.2 Subject confidentiality 80
11.3 Amendments 81
11.4 Annual progress report (country specific) 81
11.5 End of study report 81
11.6 Quality assurance 82
11.6.1 Definitions 82
11.6.2 Audits 82

12. Administrative matters 82
12.1 Financing and insurance 82
12.2 Investigator indemnity 82
12.3 Publication policy 82
12.4 Study documentation and record keeping 83
12.5 Sample handling 84

13. Trial Committees 84
13.1 Trial Development Team 84
13.2 Trial Steering Committee 85
13.3 Trial Management Team 86
13.4 Independent Data Monitoring Committee 86
13.5 Endpoint Review Committee 88
13.6 Protocol Team (the NEAT 001/ANRS 143 Agorà) 88

14. References 89

15. Appendices 93
Appendix 1: Sample patient information sheet 94
Appendix 1: Sample patient information sheet 94
Appendix 2: Sample consent form 101
Appendix 3: Sample pharmacogenetics consent 102
Appendix 4: CDC classification system for HIV infection (revision 1993) 104
Appendix 5: Serious non-AIDS events 107

Appendix 6: Table for grading adverse experiences 108
Appendix 7: Estimation of creatinine clearance (Cockcroft and Gault equation) 117
Appendix 8: Biobank procedures and assessments 118
Appendix 9: Pharmacogenetics and population pharmacokinetics................................. 121
Appendix 10: Viral sub-type analysis 123
Appendix 11: Renal function study 125
Appendix 12: Quality of life assessments 127
Appendix 13: Adherence study.. 129
Appendix 14: Pharmacoeconomics study 131
Appendix 15: Viral and immunologic dynamics and inflammation sub-study 133
Appendix 16: Bone sub-study 139
Appendix 17: Neurocognitive function sub-study 143
Appendix 18: Working groups.. 146
Appendix 19: Trial management 147
Appendix 20: Insurance policy 148

LIST OF TABLES

TABLE 1: SCHEDULE OF ASSESSMENTS
TABLE 2: DEFINITIONS OF ADVERSE EVENTS
TABLE 3: DEFINITION OF SERIOUS ADVERSE EVENTS
TABLE 4: DEFINITIONS OF CAUSALITY

LIST OF FIGURES

FIGURE 1: TRIAL ENTRY, RANDOMISATION AND TREATMENT
FIGURE 2: SAFETY REPORTING FLOWCHART

ABBREVIATIONS AND GLOSSARY

Ab	Antibody
ACTG	AIDS Clinical Trials Group
AE	Adverse event
Ag	Antigen
AIQ	Adherence-adjusted inhibitory quotient
ALAT	Alanine Aminotransferase
ANRS	Agence Nationale de Recherche sur le SIDA et les Hépatites Virales
AR	Adverse reaction
ARV	Antiretroviral
ASAT	Aspartate Aminotransferase
AUC	Area Under the Curve
BMD	Bone Mineral Density
BID	Bis In Die (twice daily)
CCR5	C-C Chemokine Receptor 5
CDC	Centers for Disease Control and Prevention
CES-D	Center for Epidemiologic Studies Depression Scale
CFR	Code of Federal Regulation
CHIP	Copenhagen HIV Programme
CI	Confidence Interval
CK	Creatinine Kinase
Cmax	Maximum Concentration
Cmin	Minimum Concentration
CNS	Central Nervous System
CPT	Vacutainer Cell Preparation Tube
CRF	Case Report Form
CT	Computer Tomography
CTA	Clinical Trials Authorisation
CTU	Clinical Trials Unit
CYP	Cytochrome P450
DCF	Data Clarification Form
dl	decilitre
DXA	Dual X-ray Absorptiometry
DRV	Darunavir
EATG	European AIDS Treatment Group
EDTA	Ethylenediaminetetraacetic acid
EFV	Efavirenz
EMEA	European Medicines Agency
EQ-5D	EQ-5D self-report questionnaire
ERC	Endpoint Review Committee
EUDRACT	European Union Drug Regulatory Agency Clinical Trial
FDA	U.S. Food and Drug Administration
FTC	Emtricitabine
g	gramme
GCP	Good Clinical Practice
GGT	Gamma Glutamyl Transpeptidase
GMP	Good Manufacturing Practice
H2	Histamine H2 receptors
HAART	Highly active antiretroviral therapy
HBc Ag	Hepatitis B Virus core antigen
HBs Ag	Hepatitis B Virus surface antigen
HBV	Hepatitis B Virus
HCG	Human Chorionic Gonadotropin
HCV	Hepatitis C Virus

HDL	High Density Lipoprotein
HIVTSQs	HIV Treatment Satisfaction Questionnaire
HLA	Human Leukocyte Antigen
HMG-CoA	3-Hydroxy-3-Methyl-Glutaryl-Coenzyme A
HOMA	Homeostasis Model Assessment
HR	Hazard Ratio
HRQoL	Health Related Quality of Life
IAS-USA	International AIDS Society-USA
IATEC	IATEC Clinical Trial Services, The Netherlands
IB	Investigator’s Brochure
IC50	50% Inhibitory Quotient
ICER	Incremental Cost Effectiveness Ratio
ICH	International Conference on Harmonisation
IEC	Independent Ethics Committee
IDMC	Independent Data Monitoring Committee
IL	Interleukine
IMP	Investigational Medicinal Product
INR	International Normalised Ratio
Inserm	Institut national de la santé et de la recherche médicale, France
IRB	Institutional Review Board
IRS	Immune Reconstitution Syndrome
ITT	Intention To Treat
kg	kilogramme
l	litre
LDL	Low Density Lipoprotein
LLN	Lower Limit of Normal
mEq	milli-equivalent
mg	milligramme
min	minute
mmHg	millimeter of mercury
ml	milliliter
mm^3	cubic millimeter
mmol	millimole
µmol	micromole
MRC	Medical Research Council (UK)
NA	Not Applicable
NCEP ATP	National Cholesterol Education Program
NEAT	European AIDS Treatment Network
NMR	Nuclear Magnetic Resonance
NNRTI	Non-Nucleoside Reverse Transcriptase Inhibitor
NRTI	Nucleoside Reverse Transcriptase Inhibitor
OBR	Optimised Background Regimen
PBMC	Peripheral Blood Mononuclear Cell
PD	Pharmacodynamics
PDE-5	Phosphodiesterase Type 5
PGx	Pharmacogenomics
PI	Protease Inhibitor
PK	Pharmacokinetics
PTH	Parathormone
QALY	Quality Adjusted Life Years
QD	Quaque Die (once daily)
QoL	Quality of Life
/r	Ritonavir boost
RAL	Raltegravir
RT	Reverse Transcriptase

SAE	Serious Adverse Event
SAR	Serious Adverse Reaction
SJS	Stevens-Johnson Syndrome
SOP	Standard Operating Procedure
SmPC	Summary of Product Characteristics
SSRI	Selective Serotonin Reuptake Inhibitors
SUSAR	Suspected Unexpected Serious Adverse Reaction
TDF	Tenofovir
TDT	Trial Development Team
TEN	Toxic Epidermal Necrolysis
TLOVR	Time to Loss Of Virologic Response
TMT	Trial Management Team
TSC	Trial Steering Committee
UAR	Unexpected Adverse Reaction
UGT	UDP-Glucuronyltransferase
UI	Unités Internationales / International Units
ULN	Upper Limit of Normal
VAS	Visual Analog Scale
VCAM	Vascular Cell Adhesion Molecule
W	Week

GENERAL INFORMATION

This document describes the NEAT 001/ANRS 143 trial and provides information about procedures for entering patients into it. The protocol should not be used as an aide-memoire or guide for the treatment of other patients; every care was taken in its drafting, but corrections or amendments may be necessary. These are circulated to the registered investigators in the trial, but centres entering patients for the first time are advised to contact the designated NEAT regional CTU to confirm they have the most up to date version. Clinical problems relating to this trial should be referred to the National Coordinating Investigator.

- **Compliance**
  The trial is to be conducted in compliance with the protocol and with international and national requirements (ICH and, as applicable, MRC GCP, Data Protection Act (DPA number g0027154)/CNIL, etc.).

- **Sponsors**
  Legal sponsor: Inserm – ANRS (Institut national de la santé et de la recherche médicale - Agence nationale de recherche sur le sida et les hépatites virales)
  101 rue de Tolbiac, 75013 PARIS, FRANCE
  Co-sponsor: European AIDS treatment network (NEAT), funded by the European Commission through FP6 Network of Excellence programme

Trial Development Team
François Raffi, Nantes, France (Chair)
Anton Pozniak, London, UK (Co-Chair)
Stefano Vella, Rome, Italy (Vice Chair)
Clotilde Allavena, Nantes, France (Clinical Coordinator)
Laura Richert, Bordeaux, France (Project Coordinator)
Andrea Antinori, Rome, Italy (Senior Trial Clinician)
José Arribas, Madrid, Spain (Senior Trial Clinician; Coordinator toxicity working group)
Abdel Babiker, London, UK (Trial Statistician)
Geneviève Chêne, Bordeaux, France (Head of Coordinating CTU)
Nikos Dedes, EATG (Community Representative)
Andrzej Horban, Warsaw, Poland (East/Central Europe coordinator)
Deenan Pillay, London, UK (Coordinator virology working group)
Brigitte Autran, Paris, France (Coordinator immunology working group)
Marta Boffito, London, UK (Coordinator pharmacology & adherence working group)
Rita Murri, Rome (Coordinator Quality of life working group)

Coordinating CTU
Inserm 897 Unit, University of Bordeaux 2, Bordeaux, France (Head: Geneviève Chêne)

Trial statistician
Abdel Babiker, MRC, London, UK

1. SUMMARY

NEAT 001/ANRS 143 Trial; protocol version 6.0, April 17, 2013

TITLE	An open-label randomised two-year trial comparing two first-line regimens in HIV-infected antiretroviral naïve subjects:<br>darunavir/r + tenofovir/emtricitabine vs. darunavir/r + raltegravir
ACRONYM	NEAT 001
SPONSORS	Inserm - ANRS (legal sponsor, trial no. ANRS 143), NEAT
TYPE	Phase III, randomised, open-label, multinational, multicenter trial (countries participating in NEAT)
INDICATION	HIV-1 infected antiretroviral naïve patients
RATIONALE	Antiretroviral treatment in HIV-infected patients requires long-term administration. Although the virologic efficacy of most antiretroviral regimens today is excellent, long-term toxicity of these treatments remains a major concern. Novel combinations of antiretroviral drugs may have the advantage of an excellent efficacy combined with little long-term toxicity and with convenient treatment modalities.<br>The triple therapy darunavir/r + tenofovir/emtricitabine is likely to become a relevant first-line treatment option in the years to come. One trial evaluating this regimen in antiretroviral naïve patients has demonstrated non-inferiority in comparison to lopinavir/r with a more favourable safety profile at 48 and 96 weeks. Significantly higher response rates were observed with darunavir/r in patients with HIV RNA > 5 log copies/ml.<br>The dual combination of boosted darunavir + raltegravir is an innovative treatment option that combines two potent new antiretroviral drugs, one of which belongs to a new drug class (integrase inhibitor). The expected efficacy profile of this combination is promising. Moreover, this combination might have a better tolerance profile and has the advantage of sparing the NRTI class.<br>In the context of tenofovir/emtricitabine currently being a reference backbone in first-line antiretroviral regimens, we hypothesise that, in combination with darunavir/r, raltegravir may be an alternative option if its efficacy is non-inferior to tenofovir/emtricitabine.
OBJECTIVES	Primary objective<br>• To assess the non-inferiority of darunavir/r + raltegravir compared to darunavir/r + tenofovir/emtricitabine as first-line treatment strategies in HIV-1 infected, antiretroviral naïve adults over 96 weeks (i.e. to assess if the absolute difference in the proportion of participants with clinical or virological failure with darunavir/r + raltegravir is at most 9% higher than the proportion of participants with clinical or virologic failure with darunavir/r + tenofovir/emtricitabine)<br>Secondary objectives<br>To compare the two different regimens in terms of:<br>• virologic response<br>• occurrence of disease progression<br>• occurrence of severe non AIDS defining events<br>• occurrence of death<br>• change in CD4 cell count<br>• occurrence of immune reconstitution syndrome<br>• genotypic resistance at virologic failure<br>• clinical and biological tolerance

	• adherence<br>• quality of life

MAIN INCLUSION CRITERIA	• Patient with confirmed HIV infection<br>• Age ≥ 18 years<br>• Written informed consent<br>• Male patient or non-pregnant, non-lactating female<br>• No previous treatment with any antiretroviral drugs<br>• HIV-1 RNA > 1000 copies/ml<br>• Indication to start an antiretroviral treatment as long as subject has also a CD4 cell count ≤ 500/mm3 either at screening or on a sample taken within 3 months before screening.<br>• No major IAS-USA mutations on genotypic testing at the screening visit or on any historical genotype, if available
MAIN NON-INCLUSION CRITERIA	• Woman without effective contraception method (recommended contraception during the trial is mechanical + a second method other than an oral contraceptive)<br>• Pregnant or breastfeeding woman<br>• Woman expecting to conceive during the study<br>• HIV-2 co-infection<br>• Creatinine clearance < 60 ml/mn (Cockcroft & Gault equation), alkaline phosphatase, ASAT, or ALAT ≥ 5 ULN<br>• Patient with significant impairment of hepatic function, defined as serum albumin < 2.8 g/dl or INR > 1.7 or presence of ascites, in the absence of another explanation for the abnormal finding<br>• CD4 > 500/mm^3 at screening except in case of symptomatic HIV disease (defined by conditions qualifying for CDC category B or C) or CD4 ≤ 500/mm3 on a sample taken within 3 months before screening.<br>• Any major IAS-USA mutation conferring resistance to one or more of reverse transcriptase or protease inhibitors on genotypic testing at screening<br>• Mycobacteriosis under treatment<br>• Malignancy requiring chemotherapy or radiotherapy<br>• Positive HBs Ag<br>• HCV infection for which specific treatment is ongoing or planned during the first year on trial treatment<br>• Known hypersensitivity to one of the trial drugs or its excipients<br>• Contraindicated concomitant treatment<br>• Anticipated non-compliance with the protocol<br>• Participation in another clinical trial with an on-going exclusion period at screening<br>• Subject under legal guardianship or incapacitation<br>• Subject, who in the opinion of the investigator, is unable to complete the study period<br>Note: For patients with acute opportunistic infection, the screening period should be as short as possible and should not exceed 2 weeks. If screening genotype results are still pending at trial entry, these patients can be enrolled under specific conditions (see full protocol).
STUDY DRUGS	Group 1: darunavir/r QD + tenofovir/emtricitabine QD (fixed dose combination)<br>Group 2: darunavir/r QD + raltegravir BID

DOSAGE AND ADMINISTRATION OF THE STUDY DRUGS	Patients are randomly allocated in a 1:1 ratio at trial entry to one of the first-line regimens of open-label treatment during at least 96 weeks with:<br><br>Group 1: darunavir/r + tenofovir/emtricitabine<br>darunavir 800 mg, i.e. 2 tablets of 400 mg (Prezista®) once daily (QD)<br>ritonavir 100 mg (Norvir®), 1 tablet once daily (QD)<br>tenofovir/emtricitabine 245/200 mg, fixed dose combination (Truvada®), 1 tablet once daily (QD)<br><br>Group 2: darunavir/r + raltegravir<br>darunavir 800 mg, i.e. 2 tablets of 400 mg (Prezista®) once daily (QD)<br>ritonavir 100 mg (Norvir®), 1 tablet once daily (QD)<br>raltegravir 400mg (Isentress®), 1 tablet twice daily (BID)
PRIMARY ENDPOINT	Time to virologic or clinical failure, as the first occurrence of any of the following components:<br>• failure to achieve virologic response by W32 (defined as HIV-1 RNA ≥ 50 copies/ml at W32, confirmed by a subsequent measurement)<br>• change of any component of the initial randomised regimen before W32 because of documented insufficient virologic response, defined as HIV-1 RNA reduction < 1 log10 copies/ml by W18 or HIV-1 RNA ≥ 400 copies/ml at W24 (confirmed by a subsequent measurement)<br>• HIV-1 RNA ≥ 50 copies/ml (confirmed by a subsequent measurement) at any time after W32<br>• death due to any cause<br>• any new or recurrent AIDS defining event confirmed by the Endpoint Review Committee<br>• any new serious non AIDS defining event confirmed by the Endpoint Review Committee
MAJOR SECONDARY ENDPOINTS	• Time to loss of virologic response by W48 and W96 (TLOVR)<br>• Time to virologic failure (as defined in primary endpoint)<br>• Time to clinical failure (as defined in primary endpoint)<br>• Time to permanent discontinuation of any component of the initial randomised regimen for treatment-limiting adverse event, i.e. intolerance or toxicity<br>• Time to discontinuation of any component of the initial randomised regimen for any reason<br>• Change in absolute CD4 count and in percentage from baseline<br>• Adverse events: number, nature and time to occurrence of clinical and biological grade 3 and 4 events<br>• Genotypic resistance at virologic failure<br>• Change in quality of life scores from W00 to W96
ANCILLARY STUDIES AND SUB-STUDIES	Viral sub-type, viral and immunologic dynamics and inflammation, kidney function, bone, neurocognitive function, pharmacogenetics, population pharmacokinetics, quality of life, adherence, pharmaco-economics
TRIAL DURATION	• Screening period: 1 to 6 weeks<br>• Treatment period: minimum 96 weeks (All patients remain in the trial until the last patient enrolled has completed the W96 visit.)<br>• Enrolment period: 14 months<br>Overall duration of the trial: 3 years

STATISTICAL CONSIDERATIONS	The study design consists of 2 parallel arms. The final analysis is based on a non-inferiority hypothesis tested by a 95% confidence interval and absolute difference in proportions calculated using Kaplan Meier. If non-inferiority of DRV/r + RAL compared to DRV/r + TDF/FTC is demonstrated, superiority will be assessed.<br>Sample size assumptions:<br>- The cumulative failure rates (virologic or clinical) for DRV/r + TDF/FTC at W32 (confirmed by W36) and W96 are 13% and 20% respectively.<br>- Recruitment period: 48 weeks<br>- Minimum follow-up: 96 weeks<br>- Cumulative probability of loss to follow-up 10% at W144<br>- Non-inferiority margin: HR = 1.53 (that is no higher than a 9% difference at W96 if failure rate for DRV/r + TDF/FTC at W96 is 20%)<br>- Power 80%<br>- Two-sided logrank test for time to failure with α = 0.05<br>Under the above assumptions, sample size is 800 patients (400 per arm) with total expected number of failures 176.
SCHEDULE	1st patient in: Q2/2010 Q3/2010<br>Last patient in: Q2/2011 Q3/2011<br>Final results: Q4/2013 Q1/2014

Flow diagram and schedule of assessments

Figure 1: Trial entry, randomisation and treatment

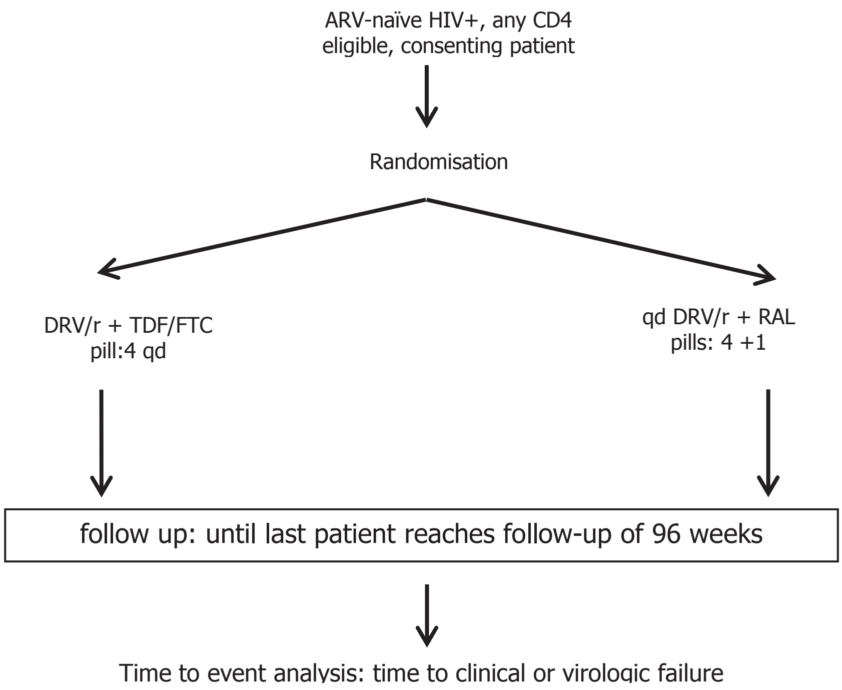

Table 1: Schedule of assessments, core protocol

**Visit**	W-6	W00	W02	W04	W08	W12	W18	W24	W32	W48	W64	W80	W96	FU-M
**Week**	**-6 to -1**	**0**	**2**	**4**	**8**	**12**	**18**	**24**	**32**	**48**	**64**	**80**	**96**	**Every 3 to 4 months**[14]
**Day**	-42 to -7	0	14±2	28±2	56±7	84±7	126± 7	168± 7	224± 7	336± 7	448± 15	560± 15	672± 15	
Informed consent	x													
Demographic data	x													
Medical / Medication history	x	x	x	x	x	x		x	x	x	x	x	x	x
Eligibility criteria	x	x												
Cardiovascular risk assessment		x						x		x			x	
Concomitant medication	x	x	x	x	x	x		x	x	x	x	x	x	x
Adverse events		x	x	x	x	x		x	x	x	x	x	x	x
Physical examination	x	x	x	x	x	x		x	x	x	x	x	x	
Morphologic evaluation		x		x	x	x		x		x			x	
Electrocardiogram		x								x			x	
Pregnancy test (beta-HCG)[1]	x	x		x	x	x	x	x	x	x	x	x	x	
Hepatitis serology[2]	x													
Plasma HIV-1 RNA[3]	x	x		x	x	x	x	x	x	x	x	x	x	x
HIV-1 genotype and subtype [4]	x								(x)	(x)	(x)	(x)	(x)	(x)
$CD4^+$ T-cells	x	x		x	x	x	x	x	x	x	x	x	x	x
$CD8^+$ T-cells		x						x		x		x	x	
Safety laboratory assessments[5]														
- haematology	x	x	x	x	x	x	x	x	x	x	x	x	x	
- chemistry[6]	x	x	x	x	x	x	x	x	x	x	x	x	x	
- coagulation	x													
- urine[7]		x	x	x	x	x	x	x	x	x	x	x	x	

Visit	W-6	W00	W02	W04	W08	W12	W18	W24	W32	W48	W64	W80	W96	FU-M
**Week**	**-6 to -1**	**0**	**2**	**4**	**8**	**12**	**18**	**24**	**32**	**48**	**64**	**80**	**96**	**Every 3 to 4 months**[14]
**Day**	-42 to -7	0	14±2	28±2	56±7	84±7	126± 7	168± 7	224± 7	336± 7	448± 15	560± 15	672± 15	
Central laboratory assessments														
- genotype[8]		x							(x)	(x)	(x)	(x)	(x)	(x)
- drug concentrations[9]				x				x						
- pharmacogenomic assessment[10]		x												
Plasma for storage[11]		x	x	x	x	x	x	x	x	x	x	x	x	x[8]
Full blood for storage[11]		x	x	x	x	x	x	x	x	x	x	x	x	
Urine for storage[11]		x		x		x				x			x	
Study drug dispensation		x		x	x	x		x	x	x	x	x	x[15]	x[15]
Drug accountability				x	x	x		x	x	x	x	x	x	x[15]
Patients' Reported Outcomes Questionnaire[12]		x		x		x		x		x			x	
In-patient care resource utilisation data[13]				x	x	x		x	x	x	x	x	x	

1 Pregnancy test in all women of child bearing potential at each visit (except visit W02): on blood sample at visit W-6, on urinary sample on other visits.
2 Hepatitis B and hepatitis C serology if not available, or if last assessment > 12 months before screening and HCV Ab negative, HBs Ab and HBc Ab negative
3 If viral load ≥50 copies/ml at Week 32, a retest needs to be performed as soon as possible, and in any case before Week 36.
4 At the screening visit (W-6 to W-1): Genotype and subtype to be performed at the local laboratory if not previously availables. At first HIV-1 RNA ≥ 500 copies/ml after 32 weeks: Genotype to be performed at the local laboratory.
5 See section 7 for details on laboratory assessments
6 Fasting for at least 10 hours
7 Urinary spot and dipstick
8 Central assessment of genotype at W00 (if not previously available or if no FASTA file is available) and at first HIV-1 RNA ≥ 500 copies/ml after 32 weeks. Storage of 1 ml of plasma (see appendices 8 and 10). Sample handling and shipment detailed in specific SOP.
9 Patients should not take their trial drugs on the morning of sampling. Sampling of 10 ml of blood (EDTA tube) and storage of plasma (see appendixes 8 and 9). Sample handling and shipment detailed in specific SOP.
10 Only in patient having signed the separate pharmacogenomics informed consent. If not performed at W00, this can be performed at any point during the trial. Sampling and storage of 1.5 ml of full blood. Sample handling and shipment specified in specific SOP.

11 Biobank for additional measurements, eg additional genotypes, proviral DNA and circular DNA, renal assessments or other. For sampling amount see appendix 8. Sample handling and shipment detailed in specific SOP.
12 Self-questionnaire: Adherence, quality of life
13 Recording of type and duration of hospital stays
14 All patients remain in the trial until the last patient enrolled has completed the W96 visit. Follow-up visits after W96 should be scheduled every three to four months and procedures performed according to standard of care. Assessments to be performed are described in chapter 7.6
15 For those patients having not reached the end of the trial (defined as the last patient's W96 visit)

2. BACKGROUND

2.1 Introduction

Antiretroviral therapy is recommended in HIV-infected patients with either symptomatic disease or, if asymptomatic, a confirmed decrease in CD4 cell count. Current guidelines recommend treatment initiation in asymptomatic patients when the CD4 cell count is < 350/mm^3 [1]. For patients with CD4 cell counts between 350 and 500/mm^3 treatment initiation may also be considered, especially in persons who have evidence of a high plasma HIV RNA level, a rapid decline in CD4 T-cells, age > 55 years, hepatitis B co-infection requiring therapy, or hepatitis C co-infection [1-3]. The clinical scenario, the presence of comorbidities, patient readiness, potential impact on quality of life, and adherence should also be considered in the decision of when and if to initiate therapy in patients with a CD4 T-cell count >350 cells/mm^3.
The current immunologic objective is to maintain HIV-infected subjects with CD4 cell counts above 500/mm^3, as mortality rates of an HIV-infected antiretroviral-treated population have reached values of the general population when CD4 counts are greater than 500/mm^3 after at least 6 years of combined antiretroviral therapy [4].

With an ever increasing armamentarium of antiretroviral drugs, there are more and more potential options for first-line treatment. To select initial treatment, emphasis is being put on identifying regimens that combine both optimal efficacy and long-term safety. In this context, issues like potency, ease of dosing, low pill burden, and good long-term tolerability have come to the foreground when comparing different therapeutic options.

Long-term toxicity of today's first-line antiretroviral regimens remains a major drawback. Although a fixed dose of the two nucleoside reverse transcriptase inhibitors (NRTIs) tenofovir and emtricitabine is to date the recommended backbone for initial therapy, there is increasing concern about renal and bone complications associated with long-term exposition to tenofovir.
Thus, there is a need for new antiretroviral agents in drug classes and new drug combinations to expand the options of initial treatment of HIV-1 infection. Novel combinations of antiretroviral drugs should have the advantage of an excellent efficacy combined with little long-term toxicity and with convenient treatment modalities.

2.2 Rationale for the NEAT 001/ANRS 143 trial

2.2.1 Standard first-line antiretroviral regimens

Triple therapies combining two nucleoside reverse transcriptase inhibitors (NRTIs) with either one protease inhibitor (PI) or one non-nucleoside reverse transcriptase inhibitors (NNRTI) are recommended for the initial antiretroviral treatment of HIV-infected adults [2,3].

The adverse effects profiles of the NNRTI- and PI-based regimens differ. NNRTI-based regimes are associated with rash and central nervous system adverse effects, whereas PI-based regimens generally are associated with more gastrointestinal symptoms and lipid abnormalities. The ACTG 5142 study showed a higher proportion of patients with lipoatrophy (>20% loss of limb fat by DXA scan) in the efavirenz (NNRTI) group than in the lopinavir/ritonavir (PI) group [5].

Drug resistance to most PIs requires multiple mutations in the HIV protease, and it seldom develops after early virologic failure, especially when ritonavir boosting is used. Resistance to efavirenz or nevirapine, however, is conferred by a single mutation in reverse transcriptase, and it develops rapidly after virologic failure [6].
All PI-based regimens include ritonavir. Some of them, e.g. darunavir combined with low dose of ritonavir (100 mg), allow for once daily regimens. Each PI has its own virologic efficacy, adverse effect profile, and pharmacokinetic properties. The choice of PI is based on virologic efficacy, the barrier for virologic resistance, and tolerability.

Darunavir containing triple therapies

Among the ritonavir-boosted protease inhibitors, ritonavir-boosted darunavir has been added as a preferred component in the latest U.S. guidelines for the use of antiretroviral agents in HIV-1-infected adults and adolescents [7], and has been approved by EMEA in antiretroviral therapy naïve adults although it is not yet licensed in the European countries. Darunavir/ritonavir has significant advantages over other approved ritonavir-boosted protease inhibitors.
Once-daily darunavir/ritonavir (800/100 mg) has been compared with ritonavir-boosted lopinavir, each in combination with tenofovir and emtricitabine, in a phase III open label randomised trial (ARTEMIS trial). The study included 689 treatment naïve subjects who had a median CD4 count of 225 cells/mm^3 and a median plasma HIV RNA level of 4.85 log10 copies/ml. Non-inferiority was demonstrated with 84% of those taking darunavir achieving HIV-1 RNA levels <50 copies/ml at 48 weeks (the primary endpoint) compared to 78 % of those taking lopinavir (95% CI of the difference: -0.1;11.0%). The rate of virologic failure > 50 copies/ml at week 48 was very low (10%), with no emergence of protease inhibitor-associated resistance mutations. Population pharmacokinetics demonstrated the consistency of active darunavir concentrations in all patients, allowing for a high inhibitory quotient [8].
At week 96, a higher proportion of patients attained HIV RNA < 50 copies/ml with darunavir/ritonavir vs. lopinavir/ritonavir (97% vs. 71%, 95% CI of the difference: 1.9% to 14.8%) and statistical superiority was demonstrated (p = .01). Darunavir/ritonavir was associated with higher rates of viral load < 50 copies/ml in patient with adverse prognostic factors (i.e. high baseline HIV RNA and low baseline CD4 cell count) [9]. Suboptimal adherence (< 95 %) to darunavir/ritonavir did not compromise virologic response, whereas patients receiving lopinavir/ritonavir who had suboptimal adherence were significantly more likely to fail therapy [10].
At week 96, treatment discontinuations occurred more frequently with lopinavir/ritonavir than with darunavir/ritonavir (23% vs 17% at week 96, respectively). Overall, there was no statistically significant difference in grade 2-4 potentially drug related clinical adverse events between the darunavir/ritonavir and lopinavir/ritonavir arms (23% and 34%, respectively). The overall incidence of rash-related adverse events (any grade, regardless of causality) was similar in the darunavir/r (15%) and lopinavir/r (13%) treatment groups. Grade 2-4 rash adverse events (all types) possibly related to study treatment were reported in 3% and 1% of the subjects in the darunavir/r and the lopinavir/r treatment groups, respectively. There was one case of Stevens-Johnson syndrome in the darunavir/r arm; although other factors may have contributed to this event, a relationship with darunavir/r could not be excluded. Darunavir was associated with statistically smaller increases in triglycerides, total cholesterol, and HDL cholesterol than lopinavir/ritonavir. The limited influence of darunavir/ritonavir on plasma lipid profiles is in part related to the low daily ritonavir dose, i.e. 100 mg [11].
Like with other protease inhibitors, hepatitis has been reported in patients receiving combination therapy with darunavir/ritonavir. In clinical trials, the rate of occurrence was 0.5%, and patients with preexisting liver dysfunction, including chronic active hepatitis B or C, had an increased risk for liver function abnormalities including severe hepatic adverse

events. Post-marketing cases of liver injury, including some fatalities, have been reported. These have generally occurred in patients with advanced HIV-1 disease taking multiple concomitant medications, having comorbidities including hepatitis B or C coinfection, and/or developing immune reconstitution syndrome [12]. Although a causal relationship with darunavir/ritonavir therapy has not been established, it is recommended to monitor liver transaminases during darunavir/ritonavir therapy, as is the case for other antiretroviral drugs and especially for protease inhibitors.

Altogether, these results suggest that once-daily ritonavir-boosted darunavir could offer advantages over lopinavir/ritonavir. Like all other protease inhibitors, darunavir has a high genetic barrier to resistance. Its favourable tolerability profile, including limited dyslipidemia, makes it an interesting alternative for first-line therapy.

2.2.2 Novel treatment strategies

Integrase inhibitor containing strategies

Among the latest antiretrovirals, integrase inhibitors represent a new class of drugs targeting one of the 3 major enzymes of the HIV intracellular replicating cycle, the viral integrase enzyme. These drugs act by blocking incorporation of the proviral HIV DNA into the host cell DNA. As productive infection requires integration of HIV DNA, an integrase inhibitor prevents an infectious virion that has progressed through reverse transcription from successfully infecting the cell [13].
The first representative of this class is raltegravir. Because of its unique mechanism of action, raltegravir does not share any cross resistance with other antiretrovirals. It induces more rapid reduction in viral decay during the second-phase decay when it is associated with other antiretroviral drugs [14].
Raltegravir is metabolised via glucuronidation (UGT1A1), is not a potent inducer or inhibitor of CYP3A4 and does not need dose adjustment when used with other antiretroviral drugs. Furthermore it would be less prone to significant drug-drug interactions with other co-medications that might be used in HIV-infected individuals.
In part 1 of the randomised, open-label, phase IIa protocol 004, 35 antiretroviral naïve subjects were randomised to receive 1 of 4 twice daily doses of raltegravir (100 mg, 200 mg, 400 mg or 600 mg twice daily) or placebo over 10 days [15]. After 10 days of monotherapy, a mean 2.0 log10 copies/ml reduction in plasma HIV-1 RNA was observed for all raltegravir treatment groups. The proportion of patients attaining HIV-1 RNA < 400 copies/ml by day 10 ranged from 50% to 60% for subjects receiving raltegravir vs. 0% for the placebo arm. No differences were observed in the slope of HIV-1 RNA decline across the raltegravir arms nor between patients with plasma HIV-1 RNA < or > 50,000 copies/ml at baseline.
In part 2 of the study, 150 additional subjects were randomised to 1 of 4 twice-daily doses of raltegravir, or efavirenz, both in combination with tenofovir plus lamivudine. At week 96, 84% vs 84% achieved HIV-1 RNA <400 copies/ml, and 83% vs 84% achieved <50 copies/ml in the raltegravir and efavirenz groups, respectively (non-completer=failure). Both raltegravir and efavirenz groups showed similar increases in CD4+ T-cells (220 vs. 232/mm^3, respectively). Two patients met the protocol definition of virologic failure after week 48, 1 in each group; no known raltegravir resistance mutations were observed. Drug-related clinical adverse events were less frequent in the raltegravir arms than in the efavirenz arm (51% vs 74%). Most frequent raltegravir-related adverse events were nausea, dizziness and headache with mild to moderate intensity. Contrary to efavirenz, raltegravir did not induce lipid changes [16,17].

In the BENCHMRK Phase III studies, patients presenting virologic failure were randomly allocated in a ratio of 2:1 to receive raltegravir 400 mg twice daily or placebo, each combined with an optimised background regimen (OBR). At week 48, the proportion of patients with HIV-1 RNA <50 copies/ml was twice as high in the raltegravir arms compared to the placebo arms (64 % vs. 34 %, respectively). Clinical and laboratory drug related adverse events in patients treated with raltegravir in the two studies were similar to placebo [18-20]. After adjustment for person-years of follow-up, the relative risk of cancer in the raltegravir groups compared to the placebo groups was 1.54 (95% CI 0.5-6.34) [21].

There is a big interest in further studying raltegravir-based regimens in treatment naïve subjects:
Results of STARTMRK protocol 021, a randomised, double-blind, phase III study, have shown that raltegravir plus tenofovir/emtricitabine was associated with non-inferior virologic efficacy vs. efavirenz plus tenofovir/emtricitabine in 563 treatment naïve patients at week 48. 86% and 82% of patients achieved HIV1-RNA<50 copies/ml in the raltegravir and in the efavirenz treatment groups, respectively [22]. The time to virologic response was significantly shorter (logrank p<.001) and CD4 cell count increase was significantly greater (+189 and +163 CD4/mm^3, 95% CI of the difference: 4-47) with raltegravir than with efavirenz. The safety profile of raltegravir was favourable with significantly less moderate to severe drug related clinical adverse events than in the efavirenz arm (16% vs. 32%, p<.001). Fewer patients in the raltegravir arm than in the efavirenz arm experienced CNS events by week 8 (10% vs 1_%, respectively; p=.015) with a persistent significant difference through week 48. Furthermore, changes in fasting lipid levels were significantly smaller with raltegravir (p<.001). Virologic response at week 48 did not differ in relation to baseline viral load (below or above 5 log copies/ml), baseline CD4 count, HCV or HBV coinfection, gender, race and B or non B viral subtype [23]. At week 96, 81% in the raltegravir group vs. 79% in the efavirenz group had HIV-1 RNA < 50 copies/ml [24].
A phase III trial comparing raltegravir QD vs. raltegravir BID both given in combination with tenofovir/emtricitabine in 750 treatment naïve patients (MK-0518-071-01 study) is underway. The primary endpoint is the proportion of patients achieving HIV RNA<50 copies/ml at week 48 in a non-inferiority design (non-inferiority margin 10%).

In a pooled analysis of five randomised trials evaluating a raltegravir-containing regimen and of an expanded access program it has been shown that there was no difference in risk of cancer in HIV-infected patients receiving raltegravir versus other antiretrovirals [25].

Based on its high antiviral potency and good tolerability, twice daily raltegravir is currently approved by the FDA and EMEA for use in treatment experienced and antiretroviral naïve patients.
Further evaluation of raltegravir-based regimens within large clinical trials with prolonged follow-up and combined endpoints is needed to assess the future role of integrase inhibitors in initial antiretroviral treatment.
Compared to NNRTIs and PIs, raltegravir containing first-line regimens could further improve virologic efficacy due to the unique antiviral properties of integrase inhibitors, with a possible stronger immunologic recovery. Furthermore, better short-term and long-term tolerability, could be expected.

NRTI-sparing first-line treatment strategies

Studies evaluating NRTI-sparing regimens as alternative first-line antiretroviral therapy have failed mainly because of high rates of discontinuations for adverse events, essentially in regard to lopinavir/ritonavir and efavirenz.

In the BIKS pilot study, the combination of lopinavir/ritonavir and efavirenz was associated with only 1 case of virologic failure over 48 weeks, but with a high rate of discontinuation (24 %) mainly due to drug-related adverse events or poor adherence [26].

ACTG 5142 has demonstrated the immuno-virologic efficacy of a dual therapy with 2 potent antiretroviral drugs, lopinavir/ritonavir and efavirenz, despite a low genetic barrier for the second one. However, tolerability was poor with frequent gastro-intestinal adverse events and severe hypertriglyceridemia [5]. The virologic efficacy of the nucleoside-sparing regimen of lopinavir/ritonavir plus efavirenz in the ACTG 5142 study clearly shows that NRTIs are not absolutely required for effective antiretroviral therapy.

NRTI-sparing regimens exploring new combinations seem desirable to expand treatment options for first-line therapy [27]. Combination of two very potent and well-tolerated antiretroviral agents like darunavir/ritonavir and raltegravir, which do not have any significant pharmacological interaction, could offer a very interesting NRTI-sparing treatment option.

Combination of darunavir/r and raltegravir

The pharmacokinetic and safety of the combination darunavir/ritonavir + raltegravir has been evaluated in a sequential 2-period study in 18 healthy subjects. In period 1, subjects were administered raltegravir 400 mg twice a day (BID) for 4 days. In period 2, subjects received raltegravir 400 mg BID + darunavir/ritonavir (600/100 mg BID) for 12 days. Eight subjects presented a clinical adverse event of rash. Seven were rated mild to moderate in intensity, one progressed to a serious adverse event of maculopapular rash. All rashes had an onset during co-administration of raltegravir + darunavir/ritonavir between day 8 and day 12 [28].

In phase III studies, evaluating raltegravir vs. placebo in treatment experienced patients with HIV infection receiving an optimised background regimens (OBR), a subset of 301 patients received raltegravir (n=200) or placebo (n=101) in combination with darunavir/ritonavir as part of the OBR. The occurrence of rash regardless of drug relationship was higher in patients receiving raltegravir + darunavir/ritonavir in OBR compared to placebo + darunavir/ritonavir (15% vs. 4%) after at least 48 weeks of therapy. However, the occurrence of rash considered to be drug-related was similar in the raltegravir + darunavir/ritonavir and placebo + darunavir/ritonavir (3.5% vs. 2%). None of the rashes was considered serious nor led to discontinuation of therapy [21].

Thus, at this stage, there is no indication that darunavir/ritonavir in combination with raltegravir is associated with increased risk of treatment-limiting cutaneous adverse events in HIV-infected patients. Such events are carefully assessed during the trial and any signal of increased incidence is to be reviewed by the Trial Steering Committee and the IDMC.

Based on limited pharmacokinetic data, co-administration of darunavir/ritonavir and raltegravir appears to result in a modest, and clinically non significant, effect on raltegravir [28]. These results are consistent with a report from the French expanded access program that includes an arm of darunavir/ritonavir + raltegravir with 83 patients and which concludes that, despite a large interpatient variability, darunavir/ritonavir combination had no deleterious effect on raltegravir minimal concentration [29].

2.2.3 Comparator groups of the NEAT 001/ANRS 143 trial

The triple combination of ritonavir-boosted darunavir and tenofovir/emtricitabine has been selected as the reference arm in this clinical trial.
Once daily darunavir/ritonavir is better tolerated than lopinavir/ritonavir and exerts an equivalent immuno-virologic efficacy overall, with a higher efficacy in the most advanced patients with either high viral load or low CD4 cell counts. It is likely to become a widely used first-line regimen in the near future.

Combination of the two very potent and well-tolerated antiretroviral agents darunavir/ritonavir and raltegravir, which do not have any significant pharmacological interactions, may offer an interesting NRTI-sparing treatment option. Compared to the triple combination of darunavir/r + tenofovir/emtricitabine, this regimen could demonstrate a better tolerability profile while having a similar or even superior antiviral potency. The second treatment arm of the NEAT 001/ANRS 143 trial therefore consists in darunavir/ritonavir + raltegravir, allowing to assess the efficacy and safety of this NRTI-sparing regimen compared to darunavir/ritonavir + tenofovir/emtricitabine.

Preliminary results of an ongoing study (PROGRESS) comparing boosted PI, namely lopinavir/ritonavir, plus raltegravir vs. lopinavir/ritonavir plus TDF/FTC in antiretroviral naïve patients confirm the interest of further studying such NRTI-sparing strategies in well-powered trials. In the PROGRESS study, the percentage of patients with a viral load below 40 copies/ml at week 8 was 81% with lopinavir/ritonavir + raltegravir versus 42% with lopinavir + TDF/FTC, without any major tolerance issues [30].

In conclusion, this study compares 2 antiretroviral combinations for the initial treatment of HIV-1 infected patients:

- a reference regimen of once-daily darunavir/ritonavir and tenofovir/emtricitabine,

- an innovative regimen of a dual combination of 2 individually potent and well tolerated drugs: once daily ritonavir-boosted darunavir and twice daily raltegravir.

In the context of tenofovir/emtricitabine currently being a reference backbone in first-line antiretroviral regimens, we hypothesise that, in combination with darunavir/r, raltegravir may be an alternative option if its efficacy is non-inferior to tenofovir/emtricitabine.

The study has a minimum duration of 2 years and assesses the relative efficacy of the 2 treatment regimens on a virologic and clinical composite endpoint, to evaluate non-inferiority of the novel ("new" and "innovative") combination over the "reference" arm.
The study assesses, in secondary endpoints more specific virologic, immunologic, pharmacologic, toxicity and quality of life aspects.

2.3 Investigational products

In this study, two combinations are compared in an antiretroviral naïve HIV-1 infected population:

- Darunavir/ritonavir (800/100 mg QD) + tenofovir/emtricitabine (245/200 mg QD)
- Darunavir/ritonavir (800/100 mg QD) + raltegravir (400 mg BID)

2.3.1 Tenofovir disoproxil difumarate and emtricitabine

Tenofovir disoproxil difumarate and emtricitabine are NRTIs. In August 2004 the FDA approved the combination of these two drugs in one fixed-dose combination tablet, i.e. Truvada®, to allow for once-a-day dosing. Each tablet contains 300 mg tenofovir disoproxil fumarate (equivalent to 245 mg tenofovir disoproxil) and 200 mg emtricitabine to be used in combination with a boosted PI or NNRTI for the treatment of HIV-1 infection in adults. The approval of Truvada® is based on safety and efficacy data that exist for both components individually, and on bioequivalence studies demonstrating similar pharmacokinetic parameters of the combination product and the individual products. Most common adverse reactions (incidence $\geq$ 10%) are diarrhea, nausea, fatigue, headache, dizziness, depression, insomnia, abnormal dreams, and rash. Truvada® should not be administered to patients with low creatinine clearance or those who require haemodialysis. Emtricitabine and tenofovir are eliminated by a combination of glomerular filtration and active tubular secretion, i.e. renal clearance in adults with normal renal function. Renal impairment, including cases of acute renal failure and Fanconi syndrome (renal tubular injury with severe hypophosphatemia), has been reported with the use of tenofovir. In a 144-week study in antiretroviral naïve patients, tenofovir has been associated with significant increases in biochemical markers of bone metabolism, suggesting increased bone turnover, and a significantly greater mean percentage decrease from baseline in bone mineral density (BMD) at the lumbar spine [31]. The effects of tenofovir-associated changes in BMD and biochemical markers on long-term bone health and future fracture risk are unknown.
Following a single oral dose, the plasma emtricitabine and tenofovir half-life are approximately 10 and 17 hours, respectively. Compared to fasted administration, dosing of tenofovir and emtricitabine in combination with either a fat meal or a light meal increased the mean area under the curve (AUC) and maximum plasma concentrations (Cmax) of tenofovir by 35% and 15%, respectively, without affecting emtricitabine exposures.

2.3.2 Darunavir / ritonavir

Darunavir is an inhibitor of the HIV-1 protease. In July 2007, the FDA approved darunavir boosted with ritonavir for use in combination with other antiretroviral agents in adults with HIV infection. In Europe, darunavir co-administered with 100 mg ritonavir is indicated in combination with other antiretroviral medicinal products for the treatment of HIV-1 infection in highly pre-treated adult patients who failed more than one regimen containing a protease inhibitor. Darunavir is extensively metabolised by CYP enzymes, primarily CYP3A. Ritonavir inhibits CYP3A4, thereby increasing the plasma concentration of darunavir. When administered with food, the Cmax and AUC of darunavir co-administered with ritonavir are higher than in the fasting state. Therefore, darunavir/ritonavir should always be taken with food but the type of food does not affect exposure to darunavir. Unmetabolised darunavir accounts for approximately 41.2% and 7.7% of the administered dose in feces and urine, respectively. The terminal elimination half-life of darunavir is approximately 15 hours when combined with ritonavir.
During the clinical development program of darunavir, severe skin rash, including erythema multiforme and Stevens-Johnson syndrome, has been reported. In some cases, fever and elevations of transaminases have also been reported. The discontinuation rate due to rash was 0.3%. Rash was generally mild to moderate, self limited maculopapular skin eruptions. The most common treatment emergent adverse events (>10%) reported in de novo subjects, regardless of frequency or causality, were diarrhoea, headache, nausea, and nasopharyngitis.

In first-line therapy, darunavir/ritonavir 800/100 mg once daily, i.e. two 400 mg tablets of darunavir plus one 100 mg capsule of ritonavir, has been evaluated with a good efficacy and tolerability profile (ARTEMIS study) [8,32]. Tablets of 400 mg have been approved by the FDA and by the EMEA in November 2008. Among the ritonavir-boosted protease inhibitors, ritonavir-boosted darunavir has been added as a preferred component in the latest U.S. guidelines for the use of antiretroviral agents of first-line therapy in HIV-1-infected adults and adolescents [7], although it is not yet licensed in the European countries.

2.3.3 Raltegravir

Raltegravir is a human immunodeficiency virus integrase strand transfer inhibitor. Commercialised under the brand name of Isentress®, it is indicated in combination with other antiretroviral agents for the treatment of HIV-1 infection in adult patients. This indication is based on safety and efficacy data from two double-blind, placebo-controlled trials of 48 weeks duration in treatment-experienced patients and one double-blind, active-controlled trial of 48 weeks duration in treatment-naïve patients [33].
With twice daily dosing, pharmacokinetic steady state is achieved within approximately the first two days of dosing. Considerable variability has been observed in the pharmacokinetics of raltegravir. Administration of raltegravir following a high-fat meal increased raltegravir AUC by approximately 19%, allowing an administration of raltegravir without regard to food. The apparent terminal half-life of raltegravir is approximately 9 hours and the major mechanism of clearance of raltegravir is UGT1A1-mediated glucuronidation. There are no contraindications. The most common adverse events (>10%) of all intensities, reported in subjects in either the raltegravir or the placebo group, regardless of causality were: nausea, headache, diarrhoea and pyrexia. Creatine kinase elevations were observed in subjects who received raltegravir. Myopathy and rhabdomyolysis have been reported; however, the relationship of raltegravir to these events is not known [33].
For the treatment of patients in the NEAT 001/ANRS 143 trial, the dosage of raltegravir is 400 mg administered orally, twice daily with or without food.

2.4 Objectives

2.4.1 Primary objective

To assess the non-inferiority of darunavir/r + raltegravir compared to darunavir/r + tenofovir/emtricitabine as first-line treatment strategies in HIV-1 infected, antiretroviral naïve adults over at least 96 weeks (i.e. to assess if the absolute difference in the proportion of participants with clinical or virological failure with darunavir/r + raltegravir is at most 9% higher than the proportion of participants with clinical or virologic failure with darunavir/r + tenofovir/emtricitabine).

2.4.2 Secondary objectives

To compare the two different regimens in terms of:

- virologic response
- occurrence of disease progression
- occurrence of severe non AIDS defining events

- occurrence of death
- change in CD4 cell count
- occurrence of immune reconstitution syndrome
- genotypic resistance at virologic failure
- clinical and biological tolerance
- adherence
- quality of life

2.5 Methodological rationale

The NEAT 001/ANRS 143 trial is designed as a non-inferiority trial.
The primary objective is to assess the non-inferiority of darunavir/r + raltegravir compared to darunavir/r + tenofovir/emtricitabine as first-line treatment strategies in HIV-1 infected, antiretroviral naïve adults over at least 96 weeks (i.e. to assess if the absolute difference in the proportion of the participants with clinical or virological failure with darunavir/r + raltegravir is at most 9% higher than the proportion of participants with clinical or virologicial failure with darunavir/r + tenofovir/emtricitabine). If non-inferiority of darunavir/r + raltegravir compared to darunavir/r + tenofovir/emtricitabine is demonstrated, superiority will be assessed.

The non-inferiority design is justified a) by the low expected virological/clinical failure rate in the reference arm at 2 years– not leaving much room for superiority and b) by the fact that besides virologic efficacy, the novel treatment arm might have advantages over the reference arm in terms of toxicity and tolerability.

The NEAT 001/ANRS 143 is thought to be a pragmatic trial evaluating different treatment strategies of already available drugs under “real life” conditions. The trial is therefore conducted as an open-label trial. An objective assessment of the primary outcome being essential in an open label trial, the primary endpoint of the NEAT 001/ANRS 143 trial has been constituted of objective components. All clinical endpoint components are to be adjudicated by an Endpoint Review Committee blinded to the random treatment allocation.

2.6 Risks and benefits

2.6.1 Darunavir/ritonavir + tenofovir/emtricitabine arm

Benefits

- Once-a-day regimen
- In treatment naïve patients, once daily darunavir/ritonavir is better tolerated than lopinavir/ritonavir and exerts an equivalent immuno-virologic efficacy overall, which is even higher in the most advanced patients with either high viral load or low CD4 cell counts [8].

Risks

- Long-term tenofovir-related risk of renal dysfunction and osteopenia remains unclear.

2.6.2 Darunavir/ritonavir + raltegravir arm

Benefits

- NRTI-sparing dual therapy, i.e. lopinavir/ritonavir + efavirenz, has shown similar efficacy to "classical" triple regimens. Lipoatrophy was less common than in the NRTI-based regimen [34]. However, this dual combination was associated with high rate of discontinuation for tolerability problems. New NRTI-sparing regimens with good efficacy and tolerance, offering alternative strategy, are needed.
- In treatment naïve patients, once daily darunavir/ritonavir is superior to lopinavir/ritonavir because of a better virologic efficacy and a better tolerability profile. Superiority is even more pronounced in the most advanced patients with high viral load or low CD4 cell counts [9].
- Better gastrointestinal tolerability and better lipid profile are expected with a low ritonavir dosage (100 mg QD).
- The risk of development of resistance to PI in case of virologic failure is very low, and has not been observed in a study with darunavir/r + tenofovir/emtricitabine [8].
- Raltegravir is a new drug with a potent antiviral effect and no cross resistance with other agents.
- No raltegravir dose-related toxicities have been identified at this time.
- No drug interactions between raltegravir and darunavir/ritonavir have been reported and no dose adaptation is needed.

Risks

- No preliminary efficacy and tolerability data are available in antiretroviral naïve HIV-infected patients, for this dual regimen.
- Contrary to clinical experience with the combination in HIV-infected treatment experienced patients, co-administration of raltegravir with darunavir/ritonavir resulted in an unexpected high incidence of rash in HIV negative healthy volunteers. No data are available with this dual regimen in HIV-infected treatment naïve patients.
- Risk of rapid emergence of raltegravir resistance mutation in case of virologic failure. However, as observed with NRTIs when combined with PI/r, the risk of resistance at failure might actually be decreased.

2.7 Overview of ancillary studies and sub-studies

2.7.1 Ancillary studies

The following ancillary studies are performed in all patients:

- Pharmacogenetics study (in consenting patients)
- Population pharmacokinetics
- Viral sub-type study
- Renal function study
- Quality of life study
- Adherence study
- Pharmacoeconomics study (in-patient resource utilisation data in all patients; detailed pharmacoeconomic assessment in Italy only)

For the full assessment items, please refer to appendices 9-14.

2.7.2 Sub-studies

The following sub-studies are performed in subgroups of patients:

- Viral and immunologic dynamics and inflammation sub-study
- Bone sub-study
- Neurocognitive function sub-study

Summaries of objectives and procedures of these sub-studies are described in appendices 15-17.

Any proposals regarding additional sub-studies or ancillary assessments must be submitted to the Trial Steering Committee and approved prior to their implementation.

3. STUDY DESIGN

This is a phase III randomised multi-centre trial to compare the efficacy and safety of two different first-line treatment strategies in HIV-1 infected, antiretroviral naïve adults over at least 96 weeks: darunavir/r + tenofovir/emtricitabine vs. darunavir/r + raltegravir. The trial continues until the last patient has completed the week 96 (W96) visit.

3.1 Primary endpoint

The primary endpoint is time to virologic or clinical failure, defined as the first occurrence of any of the following six components:

- Component 1: failure to achieve virologic response by W32 (defined as HIV-1 RNA ≥ 50 copies/ml at W32, confirmed by a subsequent measurement*)
- Component 2: change of any component of the initial randomised regimen before W32 because of documented insufficient virologic response, defined as HIV-1 RNA reduction < 1 $\log_{10}$ copies/ml by W18 or HIV-1 RNA ≥ 400 copies/ml at W24 (confirmed by a subsequent measurement*)).
- Component 3: HIV-1 RNA ≥ 50 copies/ml (confirmed by a subsequent measurement*)) at any time after W32
- Component 4: death due to any cause
- Component 5: any new or recurrent AIDS defining event (category C events as defined in appendix 4) confirmed by the Endpoint Review Committee
- Component 6: any new serious non AIDS defining event, as defined in appendix 5, confirmed by the Endpoint Review Committee

*In case of treatment modification for virological reasons, it is mandatory to take the retest sample before any treatment modification.

3.2 Secondary endpoints

Secondary endpoints are:

- Time to loss of virologic response (TLOVR) by W48 and W96 defined as time to virologic failure (as defined in primary endpoint, components 1-3) or regimen discontinuation for any reason or death
- Time to virologic failure (as defined in primary endpoint, components 1, 2 and 3)
- Time to clinical failure (as defined in primary endpoint, components 4, 5 and 6)
- Time to permanent discontinuation of any component of the initial randomised regimen for treatment-limiting adverse event, i.e. intolerance or toxicity
- Time to discontinuation of any component of the initial randomised regimen for any reason
- Time to loss of virologic response by end of follow-up defined as time to virologic failure (as defined in primary endpoint, components 1-3) or regimen discontinuation for any reason or death
- Change in absolute CD4 count and in percentage from baseline
- Adverse events: number, nature and time to occurrence of clinical and laboratory grade 3 and 4 events
- Genotypic resistance at virologic failure
- Change in quality of life scores between W0 and W96

3.3 Other endpoints

- Time to HIV-1 RNA ≥ 50 copies/ml for patients with HIV-1 RNA < 50 copies/ml at some point
- Proportion of patients with HIV-1 RNA < 50 copies/ml at W24, W32, W48, and W96, respectively
- Proportion of patients with immune reconstitution syndrome
- Time to occurrence of any cardiovascular event requiring unplanned hospitalisation
- Change in patient reported symptoms between W00 and W96
- Time to clinical or virologic failure (as defined in the primary endpoint) in patients with low CD4 counts at W00 (CD4 $< 50/mm^3$; CD4 $< 200/mm^3$)
- Time to clinical or virologic failure (as defined in the primary endpoint) in patients with high HIV-1 RNA at W00 (HIV-1 RNA > 5 log copies/ml)
- Time to CD4 count $> 350/mm^3$ and $> 500/mm^3$, respectively, in patients with CD4 counts below these limits at W00
- Time to clinical failure (as defined in primary endpoint, components 4-6)

3.4 Trial calendar

- Enrolment period: planned 12 months; actual 14 months
- Duration of patient's participation: minimum 96 weeks plus a 1-6 week screening period
- All patients remain in the trial until the last patient enrolled has completed the W96 visit.
- The average expected follow-up for a patient was planned to be 120 weeks with range 96 to 144 weeks; given the longer enrolment period then average expected follow-up for a patient will be 121 weeks and will range from 96 to 150 weeks.
- Overall duration of the trial: 3 years

The trial is scheduled to start in the second trimester 2010.

4. STUDY POPULATION

4.1 Number and type of subjects

In this study, 800 patients will be enrolled. All patients who satisfy the following inclusion and non-inclusion criteria are eligible.

4.2 Inclusion criteria

- Patient with confirmed HIV-1 infection (HIV antibody positive confirmation prior to screening)
- Age ≥ 18 years
- Written informed consent
- Male patient or non-pregnant, non-lactating female patient
  (Women of childbearing age must have a negative serum pregnancy test at screening. All female participants of child bearing potential must use contraception for the month preceding study entry and for the entire duration of the study. Recommended contraception during the trial is mechanical + a second method other than an oral contraceptive)
- No previous treatment with any antiretroviral drugs while being seropositive (including single dose administration, for example to prevent mother-to-child transmission). Having received ART while seronegative (post-exposure prophylaxis (PEP) or pre-exposure prophylaxis (Prep)) is not a non-inclusion criterion as long as a HIV negative test at least 3 months post-PEP or -Prep is documented (and so patients having participated in HIV vaccine studies before inclusion may be included in the trial).
- HIV-1 RNA > 1000 copies/ml
- Indication to initiate ARV therapy according to current guidelines, as long as subject has also a CD4 cell count ≤ 500 /mm^3 either at screening or on a sample taken within 3 months before screening.
- No major IAS-USA mutations on genotypic testing at the screening visit or on any historical genotype, if available
  (December 2009 list, see http://www.iasusa.org/resistance_mutations)

- For French volunteers only: subjects covered by Health Insurance

4.3 Non-inclusion criteria

- Woman of child-bearing potential without effective contraception method.
  For contraception during the trial all women must use a combination of mechanical method and a second method other than an oral contraceptive. Oral contraceptives are not to be used during the trial unless there is a medical indication. In these circumstances women must also use a mechanical method plus a second method other than an oral contraceptive and this is not a non-inclusion criterion.
- Pregnant or breastfeeding woman
- Woman expecting to conceive during the study period
- HIV-2 co-infection
- Creatinine clearance < 60 ml/mn (estimated glomerular filtration rate according to the Cockcroft & Gault equation), alkaline phosphatase, ASAT or ALAT ≥ 5 times the upper limit of the norm (ULN)
- Patient with significant impairment of hepatic function, defined as serum albumin < 2.8 g/dl or INR > 1.7 or presence of ascites, in the absence of another explanation for the abnormal finding
- CD4 > 500/mm^3 at screening except in case of symptomatic HIV disease (defined by conditions qualifying for CDC category B or C, see appendix 4) or CD4 ≤ 500/mm^3 on a sample taken within 3 months before screening.
- Any major IAS-USA mutation conferring resistance to one or more of reverse transcriptase or protease inhibitors on genotypic testing at screening or on any historical genotype if available (December 2009 list, see http://www.iasusa.org/resistance_mutations). Any previous genotype result is valid, with no time limit, as long as the original test result is documented.
- Mycobacteriosis under treatment
- Malignancy requiring chemotherapy or radiotherapy
- Positive HBs Ag
- HCV infection for which specific treatment is ongoing or planned during the first year on trial treatment. Protocol-specified concomitant hepatitis C treatment is allowed after the first 12 months on trial treatment.
- Known hypersensitivity to one of the trial drugs, the metabolites or formulation excipients
- Contraindicated concomitant treatment
- Anticipated non-compliance with the protocol
- Participation in another clinical trial with an on-going exclusion period at screening
- Subject under legal guardianship or incapacitation
- Subject, who in the opinion of the investigator, is unable to complete the study period

Resistance testing at screening
In accordance with current HIV treatment guidelines, mutations conferring resistance to reverse transcriptase or protease inhibitors are assessed on genotypic testing at screening or on historical genotype if available. Genotypic testing is performed in the local laboratory. Any

previous genotype result is valid, with no time limit, as long as the original test result is documented. Raltegravir resistance testing is not required at screening, since this is currently not recommended in patients naïve to integrase inhibitors and not widely available.

Additional sequencing of integrase will be performed by centralised laboratories using biobank samples to correlate baseline mutations to integrase and virologic outcome (see viral sub-type analysis, appendix 10).

Patients with acute opportunistic infections

Rapid introduction of antiretroviral therapy seems the best option in patients with acute opportunistic infections [35]. For patients with acute opportunistic infection, the screening period should therefore be as short as possible and should not exceed 2 weeks.

Results of genotypic testing should be available before randomisation. However, if the patient presents with an acute opportunistic infection requiring urgent initiation of antiretroviral therapy, the patient may be enrolled and start trial treatment even if the results of the genotype are still pending. In this case, one of the following conditions must be fulfilled, to enrol the patient in the trial:

- The patient has an acute opportunistic infection requiring urgent initiation of antiretroviral therapy (as judged by the investigator) and there is no evidence of any major IAS-USA mutation conferring resistance to one or more of reverse transcriptase or protease inhibitors mutations on a historical genotype.
- The patient has an acute opportunistic infection requiring urgent initiation of antiretroviral therapy (as judged by the investigator) and there is no historical gentotype available and screening genotyping is underway, but results are not yet available.

4.4 Enrolment recommendations

Enrolment of both genders
Attention should be paid that the proportion of women included in the trial is representative of the epidemiological situation of HIV infection in participating countries.

Enrolment of patients with CD4 counts < $200/mm^3$
Patients with CD4 counts <$200/mm^3$ can be enrolled in the trial. During the trial, the site investigator should carefully evaluate the individual virologic and clinical evolution of these patients and adhere to current guidelines for management of patients with low CD4 cell counts, such as prophylaxis of opportunistic infections.

5. INVESTIGATIONAL PRODUCTS

5.1 Introduction

Patients are randomised in a 1:1 ratio between darunavir/r + tenofovir/emtricitabine and darunavir/r + raltegravir.

Participating patients must have the intention at baseline to continue the antiretroviral regimen during the course of the trial. If, for any reason, the HAART regimen is being changed or interrupted, the patient remains in the study programme, and the changes and/or interruption are documented.

5.2 Name and description of the investigational medicinal products

5.2.1 Darunavir/r + tenofovir/emtricitabine arm

The study medications used in this arm are:

- darunavir 800 mg, i.e. 2 tablets of 400 mg (Prezista®) once daily (QD), orally
  and
- ritonavir 100 mg (Norvir®), 1 tablet once daily (QD), orally
  and
- tenofovir/emtricitabine 245/200 mg, fixed dose combination (Truvada®), 1 tablet once daily (QD), orally

from W00 to the end of patient follow-up (when last patient reaches W96).

Specific data about the study medication can be found in the Summary of Product Characteristics (SmPC).
The first patients enrolled started the trial with RTV capsules. RTV tablets have become available for the trial in early 2011, and all trial participants are now being prescribed ritonavir tablets, as specified a priori in protocol version 1.0.
No changes in dosage are allowed during the trial, except for an adaptation of dosage of tenofovir in case of GFR < 50 ml/min (see SmPC).

5.2.2 Darunavir/r + raltegravir arm

The study medications used in this arm are:

- darunavir 800 mg, i.e. 2 tablets of 400 mg (Prezista®) once daily (QD), orally
  and
- ritonavir 100 mg (Norvir®), 1 tablet once daily (QD), orally
  and
- raltegravir 400mg (Isentress®), 1 tablet twice daily (BID), orally

from W00 to the end of patient follow-up (when last patient reaches W96).

Specific data about the study medication can be found in the Summary of Product Characteristics (SmPC).
The first patients enrolled started the trial with RTV capsules. RTV tablets have become available for the trial in early 2011, and all trial participants are now being precribed ritonavir tablets, as specified a priori in protocol version 1.0.
No changes in dosage are allowed during the trial. Should data on raltegravir 800 mg once daily become available during the trial, appropriate decisions will be taken by the Trial Steering Committee.

5.3 Provision of the trial drugs

Truvada® is provided by Gilead Sciences.
Isentress® is provided by Merck.
Prezista® and Norvir® are provided by Janssen-Cilag.

5.4 Packaging and labelling

All medications in this study are prepared and packed by their manufacturers in accordance with Good Manufacturing Practice (GMP) and applicable local laws and regulations.

The medications are packed in bottles. A specific trial label is added to each bottle in compliance with GMP by a pharmaceutical service provider, subcontracted by the legal sponsor. The label attached to the bottles includes the following information (country-dependent):

- Name and contact details of the sponsor, CTU or investigator
- Route of administration
- Lot number and batch number
- Trial reference code
- Trial subject identification code
- Quantity of bottle content
- Dosage
- Expiry date
- Storage conditions
- 'For clinical trials only'
- 'Keep out of reach of children'

5.5 Handling and storage

The study medications are shipped to the study site under controlled conditions and are stored in a locked environment. The responsible pharmacist or investigator maintains a temperature control log and medication inventory.

Details on handling, shipment and storage of trial drugs are specified in a separate document (trial specific SOP).

5.6 Responsibilities

In compliance with ICH GCP, the responsibility for the study medication at the study site lies with the investigator and pharmacist and includes the following:

- Receipt of the study medication
- Documentation of deliveries
- Adequate storage of study medication
- Keeping of records including dates, quantities and the unique code numbers assigned to the investigational medication and trial subjects
- Documentation including the administration of the study medication to subjects as specified by the protocol
- Reconciliation of all investigational medications

The following guidelines apply:

- The investigator agrees not to supply any medication to any person except to the patients included in the study.
- The investigator/pharmacist keeps the medication in a locked secure storage facility, only accessible to authorised staff.
- A study medication inventory is maintained, including all materials received and dispensed to patients.
- At the conclusion or termination of the study, the investigator/pharmacist agrees to conduct a final drug supply inventory and to record this on a drug accountability form.

5.7 Accountability

Accountability includes documentation (on a study-specific accountability log) of the numbers of medications dispensed by the pharmacist to the investigator or investigator to the participant. Unused study drug should be returned to the study centre at the end of the trial in order to allow for drug accountability.

6. CONCOMITANT MEDICATION

Co-medications, including drugs used for the primary or secondary prophylaxis of opportunistic infections, may be continued for the duration of the study. All medications have to be documented.

6.1 Hepatitis C treatment

Chronic hepatitis C
Investigators are encouraged to perform liver fibrosis staging and to evaluate the indication for HCV therapy in HIV/HCV co-infected patients before enrolment into the trial. If HCV treatment is indicated, it should either be administered before enrolment into the trial, or the patient should not be enrolled in the trial.
Concomitant hepatitis C treatment is allowed after the first 12 months on study drug but should be restricted to patients with a clear indication for hepatitis C treatment.

Patients requiring hepatitis C treatment should be treated with pegylated interferon-alpha and weight-adapted dose of ribavirin, according to current guidelines [36]. For updates of guidelines refer to http://www.eacs.eu/guide/index.htm.

Other hepatitis C treatments, including investigational drugs or regimens, should not be used during the trial.

Acute hepatitis C
Treatment of acute HCV is discouraged during first year of the trial. There is no strong evidence that delaying treatment of acute Hep C for a few weeks will change response.
In the very rare situation that a patient develops acute HCV during first year of the trial, HCV treatment should only be initiated if there is a strong indication not to wait until week 48. The investigator should contact the regional CTU. Any decision to initiate HCV treatment during the first year of the trial should be taken only after discussion within the Trial Management Team.
Treatment should be based on current national or European guidelines. For updates, refer to http://www.eacs.eu/guide/index.htm.

6.2 Not permitted medications / Precautions

6.2.1 HIV medication

Prescription of HIV vaccines and investigational antiretroviral drugs, except study drugs, are not allowed during the trial.

6.2.2 Tenofovir/emtricitabine containing arm

Drugs interaction studies have been conducted with emtricitabine and tenofovir disoproxil fumarate, the components of Truvada®.

No drug interaction studies have been conducted using Truvada® tablets. Emtricitabine and tenofovir are primarily excreted by the kidneys by a combination of glomerular filtration and active tubular secretion. No drug-drug interactions due to competition for renal excretion have been observed; however, coadministration of Truvada® with drugs that are eliminated by active tubular secretion may increase concentrations of emtricitabine, tenofovir, and/or the coadministered drug. Some examples include, but are not limited to acyclovir, adefovir dipivoxil, cidofovir, ganciclivir, valacyclovir and valganciclovir. Drugs that decrease renal function may increase concentrations of emtricitabine and/or tenofovir (see SmPC of Truvada®).

6.2.3 Darunavir / ritonavir containing arms

Darunavir and ritonavir are both inhibitors of CYP3A. Co-administration of darunavir/ritonavir with drugs primarily metabolised by CYP3A may result in increased plasma concentration of such drugs, which could increase or prolong their hepatic effect and adverse events (see SmPC of Prezista® and Norvir®).

Drugs that should not be co-administered with darunavir/ritonavir:

- α_1-adrenoreceptor antagonist: alfuzosin

  Darunavir/ritonavir should not be used in combination with alfuzosin since this may cause increased plasma concentrations of alfuzosin which may lead to severe hypotension

- Analgesics: pethidine, piroxicam, propoxyphene

  Darunavir/ritonavir should not be used in combination with pethidine, piroxicam or propoxyphene, as this may cause increased plasma concentrations of these analgesics and thereby increases the risk of serious respiratory depression or haematologic abnormalities, or other serious adverse effects from these agents.

- Antiarrhythmics: amiodarone, bepridil, encainide, flecanide, propafenone, lidocaine (systemic), quinidine

  Concentrations of these agents may be increased when co-administered with darunavir/ritonavir, thereby, increasing the risk of arrhythmias or other serious adverse effects from these agents.

- Antibiotics: fusidic acid

  Co-administration with darunavir/ritonavir can result in increased plasma concentrations of fusidic acid and ritonavir.

- Anticonvulsant drugs: phenobarbital, phenytoïne

  Phenobarbital and phenytoïne are inducers of CYP450 enzymes. Darunavir/ritonavir should not be used in combination with phenobarbital or phenytoïne as coadministration may cause significant decreases in darunavir plasma concentrations. This may result in loss of therapeutic effects of darunavir.

- Antihistamines: astemizole, terfenadine.

  Contraindicated due to potential for serious and/or life-threatening reactions such as cardiac arrhythmias.

- Antimycobacterial: rifampicin.

Rifampicin is an inducer of CYP450 metabolism. Darunavir/ritonavir should not be used in combination with rifampicin, as this may cause significant decreases in darunavir plasma concentrations. This may result in loss of therapeutic effects of darunavir.

- Ergot derivatives: dihydroergotamine, ergonovine, ergotamine, methylergonovine

  Contraindicated due to potential for serious and/or life-threatening reactions such as acute ergot toxicity characterised by peripheral vasospasm and ischemia of the extremities and other tissues.

- Gastrointestinal motility agent: cisapride

  Contraindicated due to potential for serious and/or life-threatening reactions such as cardiac arrhythmias.

- HMG-CoA reductase inhibitors: simvastatine, lovastatine

  Potential for serious reactions such as risk of myopathy including rhabdomyolysis.

- Herbal products: St.-John's wort and Hypericum perforatum-containing products

  Coadministration with darunavir/ritonavir can cause significant decreases in darunavir/ritonavir concentrations. This may result in loss of therapeutic effect of darunavir.

- Neuroleptics/anti-psychotics: pimozide, clozapine, sertindole

  Contraindicated due to increased plasma concentrations of clozapine, pimozide and sertindole, and potential for serious and/or life-threatening reactions such as cardiac arrhythmias

- Oral contraceptives/oestrogen: ethynil estradiol, norethindrone.

  Plasma concentrations of ethynil estradiol may be decreased due to induction of its metabolism by ritonavir. Mechanical contraception + a second method other than an oral contraceptive should therefore be used during the trial. However, if there is a medical reason for a woman to use oral contraceptives during the trial in addition to mechanical contraception + a second method other than an oral contraceptive, this is not a formal contraindication.

- PDE-5 inhibitor: sildenafil

  Sildenafil is contraindicated only when used for the treatment of pulmonary arterial hypertension, given the increased risk for sildenafil-associated adverse events such as hypotension and syncope. In patients with erectile dysfunction, sildenafil should be used with caution (see below).

- Sedative/hypnotics: Clorazepate, diazepam, estazolam, flurazepam, oral midazolam, pethidine, and triazolam

  Contraindicated due to potential for serious and/or life-threatening reactions such as prolonged or increased sedation or respiratory depression.

Other potentially significant drug interactions:

Alterations in dose or regimen may be recommended.

- Analgesics: buprenorphine, fentanyl, methadone, morphine

  The clinical relevance of the increase in norbuprenorphine pharmacokinetic parameters has not been established. Dose adjustment for buprenorphine may not

be necessary when co-administered with darunavri/ritonavir but a careful clinical monitoring for signs of opiate toxicity is recommended.

Ritonavir is expected to increase the plasma concentrations of fentanyl. Careful monitoring of therapeutic and adverse effects is recommended when fentanyl is concomitantly administered with darunavir/ritonavir.

When methadone is co-administered with darunavir/ritonavir, patients should be monitored for opiate abstinence syndrome, as ritonavir is known to induce the metabolism of methadone, leading to a decrease in its plasma concentrations. Increased methadone dose may be necessary when concomitantly administered for a longer period of time.

Morphine levels may be decreased due to induction of glucuronidation by co-administered ritonavir.

- Anti-arrhythmic: digoxin, disopyramide, mexiletine

  Digoxin AUC is increased with darunavir/ritonavir. Initial recommended dosage of digoxin: as low as possible, then progressive increase

  Cardiac and neurologic events have been reported when ritonavir has been co-administered with disopyramide or mexiletine. The possibility of drug interaction cannot be excluded.

- Anti-asthmatic: theophylline

  An increased dose of theophyline may be required when coadministered with darunavir/ritonavir, due to induction of CYP1A2 by ritonavir.

- Antibiotics: clarithromycin, erythromycin

  Caution should be exercised when clarithromycin is combined with darunavir/ritonavir. No dose adjustment of darunavir and clarithromycin is required for patients with normal renal function. For patients with renal impairment the following adjustment should be considered: Creatinine clearance 30-60 ml/min, the dose of clarithromycin should be reduced by 50%. Creatinine clearance < 30 ml/min, the dose of clarithromycin should be reduced by 75 %. Clarithromycin doses greater than 1 g per day should not be coadministered with darunavir/ritonavir.

  Ritonavir is expected to increase the plasma concentrations of erythromycin. Careful monitoring of therapeutic and adverse effects is recommended when erythromycin is used concomitantly administered with darunavir/ritonavir.

- Anti-cancer agents: vincristine, vinblastine

  Serum concentrations may be increased when co-administered with darunavir/ritonavir resulting in the potential for increased incidence of adverse events.

- Anticoagulant: warfarin

  Warfarin concentrations may be affected when co-administered with darunavir/ritonavir. It is recommended that the INR be monitored when warfarin is combined with darunavir/ritonavir.

- Anticonvulsant drugs: carpamazepine, divaloprex, lamotrigine

  Ritonavir inhibits CYP3A4 and as a result is expected to increase the plasma concentrations of carbamazepine. If there is a need to combine darunavir/ritonavir and carbamazepine, patients should be monitored for potential carbamazepine-related adverse events. Carbamazepine concentrations should be monitored and its

dose should be titrated for adequate response. Based upon the findings, the carbamazepine dose may need to be reduced by 25% to 50%. No dose adjustment for darunavir/ritonavir is recommended.

Ritonavir is expected to decrease the plasma concentrations of divaloprex and lamotrigine. Careful monitoring of serum levels or therapeutic effects is recommended when these medicines are concomitantly administered with darunavir/ritonavir.

- Antidepressant: trazodone, nefazadone

Concomitant use of trazodone and darunavir/ritonavir may increase plasma concentrations of trazodone. Adverse events like nausea, dizziness, hypotension and syncope have been observed following co-administration of trazodone and ritonavir. The combination of trazodone and ritonavir should be used with caution and trazodone should be initiated at the lowest dosage.

Cardiac and neurologic events have been reported when ritonavir has been co-administered with nefazadone. The possibility of drug interaction cannot be excluded.

- Antifungals: clotrimazol, ketoconazole, itraconazole, voriconazole.

Caution is warranted and clinical monitoring is recommended, when co-administration of darunavir/ritonavir and clotrimazole is required.

Ketaconazole and itraconazole are potent inhibitors as well as substrates of CYP3A. Concomitant use with darunavir/ritonavir may increase plasma concentration of darunavir. When co-administration is required the daily dose of ketoconazole or itraconazole should not exceed 200 mg. Ritonavir is expected to increase the plasma concentrations of ketoconazole and itraconazole. Careful monitoring of therapeutic and adverse effects is recommended when itraconazole is used concomitantly administered with ritonavir. Due to an increased incidence of gastrointestinal and hepatic adverse events, a dose reduction of ketoconazole should be considered when coadministered with ritonavir.

The administration of voriconazole with ritonavir (100 mg twice daily) decreased the AUC of voriconazole by an average of 39 %. Voriconazole should not be administered to patients receiving darunavir/ritonavir unless an assessment of the benefit/risk ratio justifies the use of voriconazole.

- Antihistamines: fexofenadine, loratadine

Ritonavir is expected to increase the plasma concentrations of both agents. Careful monitoring of therapeutic and adverse effects is recommended when loratidine is concomitantly administered with darunavir/ritonavir.

- Antimalarial: atovaquone

Ritonavir induces glucuronidation and as a result is expected to decrease the plasma concentrations of atovaquone. Careful monitoring of serum levels or therapeutic effects is recommended when atovaquone is concomitantly administered with darunavir/ritonavir.

- Antimycobacterial: rifabutin

Rifabutin is an inducer and substrate of CYP450 enzymes. Concomitant use of rifabutin and darunavir in the presence of ritonavir is expected to increase rifabutin plasma concentrations and decrease darunavir plasma concentrations. When indicated, it is recommended to administer rifabutin at a dosage of 150 mg once every other day when coadministered with darunavir/ritonavir.

- β2-agonist: salmeterol

  Ritonavir inhibits CYP3A4 and as a result a pronounced increase in the plasma concentrations of salmeterol is expected. Therefore concomitant use is not recommended.

- Calcium channel blockers: felodipine, nifedipine, nicardipine

  Plasma concentration of calcium channel blockers may increase when darunavir/ritonavir is co-administered. Caution is warranted and clinical monitoring of patients is recommended.

- Corticosteroid: systemic dexamethasone, prednisolone, fluticasone propionate, budenoside

  Use with caution. Systemic dexamethasone induces CYP3A and can thereby decrease darunavir plasma concentrations. Ritonavir is expected to increase the plasma concentrations of dexamethasone and prednisolone. Careful monitoring of therapeutic and adverse effects is recommended when dexamethasone or prednisolone are concomitantly administered with darunavir/ritonavir.

  Concomitant use of inhaled fluticasone propionate or budenoside and darunavir/ritonavir may increase plasma concentrations of these corticosteroids. Alternatives should be considered, particularly for long-term use.

- H2-receptor antagonists and proton pump inhibitors: omeprazole, ranitidine.

  Darunavir/ritonavir can be administered with H2-receptor antagonists and proton pump inhibitors without any dose adjustments.

- HMG-CoA reductase inhibitors: atorvastatin, pravastatin, rosuvastatin

  When atorvastatin or rosuvastatin and darunavir/ritonavir are co-administered, it is recommended to start with the lowest possible dose of atorvastatin or rosuvastatin with careful monitoring. When pravastatin and darunavir/ritonavir are co-administered, the mean increase in pravastatin AUC is 81 %. However, pravastatin AUC increases by up to 5-fold in some subjects. The mechanism of this interaction is unknown. It is recommended to start with the lowest possible dose of pravastatin.

- Immunosuppressants: ciclosporin, everolimus, tacrolimus, sirolimus

  Plasma concentrations of ciclosporin, everolimus, tacrolimus or sirolimus may be increased when co-administered with darunavir/ritonavir. Therapeutic concentration monitoring of the immunosuppressive agent is recommended

- PDE-5 inhibitors: sildenafil, vardenafil, tadalafil

  Concomitant use of PDE-5 inhibitors for treatment of erectile dysfunction should be done with caution. If required, recommended dosage is:

  - sildenafil: ≤ 25 mg /48 hours
  - vardenafil: ≤ 2,5 mg /72 hours
  - tadalafil: ≤ 10 mg /72 hours

  Concomitant use of darunavir/ritonavir and sildenafil is contraindicated in pulmonary arterial hypertension.

- Sedative/hypnotics: alprazolam, buspirone, parenteral midazolam

  Alprazolam metabolism is inhibited following the introduction of ritonavir. After ritonavir use for 10 days, no inhibitory effect of ritonavir has been observed. Caution

is warranted during the first several days when alprazolam is co-administered with ritonavir, before induction of alprazolam metabolism develops.

Caution should be used with co-administration of darunavir/ritonavir and parenteral midazolam. Data from concomitant use of parenteral midazolam with other protease inhibitors suggest a possible 3 – 4 fold increase in midazolam plasma levels. If darunavir/ritonavir is co-administered with parenteral midazolam, it should be done in an intensive care unit (ICU) or similar setting which ensures close clinical monitoring and appropriate medical management in case of respiratory depression and/or prolonged sedation. Dosage adjustment for midazolam should be considered, especially if more than a single dose of midazolam is administered.

Ritonavir is expected to increase the plasma concentrations of buspirone. Careful monitoring of therapeutic and adverse effects is recommended when buspirone concomitantly administered with darunavir/ritonavir.

- Selective serotonin reuptake inhibitors (SSRIs): paroxetine, sertraline

  If sertraline or paroxetine are co-administered with darunavir/ritonavir, the recommended approach is a careful dose titration of the SSRI based on a clinical assessment of antidepressant response. In addition, patients on a stable dose of sertraline or paroxetine who start treatment with darunavir/ritonavir should be monitored for antidepressant response.

- Sleeping agent: zolpidem

  Zolpidem and ritonavir may be co-administered with careful monitoring for excessive sedative effects.

- Smoking cessation: bupropion

  Concurrent administration of bupropion with repeated doses of ritonavir is expected to decrease bupropion levels. These effects are thought to represent induction of bupropion metabolism. However, because ritonavir has also been shown to inhibit CYP2B6 in vitro, the recommended dose of bupropion should not be exceeded. In contrast to long-term administration of ritonavir, there was no significant interaction with bupropion after short-term administration of low doses of ritonavir (200 mg twice daily for 2 days), suggesting reductions in bupropion concentrations may have onset several days after initiation of ritonavir co-administration.

In addition to the interactions listed above, as ritonavir is highly protein bound, the possibility of increased therapeutic and toxic effects due to protein binding displacement of concomitant medications should be considered.

6.2.4 Raltegravir containing arm

Raltegravir does not inhibit CYP1A2, CYP2B6, CYP2C8, CYP2C9, CYP2C19, CYP2D6 nor CYP3A4 in vitro. Moreover, in vitro, raltegravir does not induce CYP3A4. A midazolam drug interaction study confirmed the low propensity of raltegravir to alter the pharmacokinetics of agents metabolised by CYP3A4 in vivo by demonstrating a lack of effect of raltegravir on the pharmacokinetics of midazolam, a sensitive CYP3A4 substrate. Similarly, raltegravir is not an inhibitor of the UDP-glucuronyltransferases (UGT) tested (UGT1A1, UGT2B7), and raltegravir does not inhibit P-glycoprotein-mediated transport. Based on these data, raltegravir is not expected to affect the pharmacokinetics of drugs that are substrates of these enzymes or of

P-glycoprotein (e.g., protease inhibitors, NNRTIs, methadone, opioid analgesics, statins, azole antifungals, proton pump inhibitors, oral contraceptives, and anti-erectile dysfunction agents). In drug interaction studies, raltegravir did not have clinically meaningful effects on the pharmacokinetics of lamivudine nor tenofovir.

Caution should be used when co-administering raltegravir with strong inducers of uridine diphosphate glucuronyltransferase (UGT)1A1 (e.g. rifampin) due to reduced plasma concentrations of raltegravir. The impact of other inducers of drug metabolising enzymes, such as phenytoin and phenobarbital, on UGT1A1 is unknown. Other less strong inducers (e.g., efavirenz, nevirapin, rifabutin, St-John’s wort) may be used with the recommended dose of raltegravir.
In healthy subjects, co-administration of raltegravir with omeprazole increases raltegravir plasma levels. As the effects of increasing gastric pH on the absorption of raltegravir in HIV-infected patients are uncertain, use raltegravir with medicinal products that increase gastric pH (e.g. proton pump inhibitors and H2 antagonists) only if unavoidable (see SmPC of Isentress®).

6.3 Data on concomitant medication

For all concomitant medications the following data need to be recorded:
commercial name of the product, formulation, dosage per drug unit, prescribed dosage, indication, start date and stop date.

7. STUDY CONDUCT

7.1 Signature of informed consent

Written informed consent must be obtained prior to the initiation of any study related intervention.
The investigating physician informs the patient of the objectives, type of constraints and foreseeable risks of the study. The investigator is responsible for ensuring that the subject fully understands the nature and purpose of the study. Information should be given in both oral and written form. No subject should be obliged to participate in the study. Subjects must be given ample opportunity to enquire about details of the study. The information must make clear that refusal to participate or withdrawal from the study at any stage is without any prejudice to the subject's subsequent care. Women should be informed about possible risks in case of pregnancy and advised not to become pregnant during the trial.
Subjects must be allowed sufficient time to decide whether or not they wish to participate.

The subject must be made aware of (and give consent to) the fact that monitors, auditors, and representatives of Independent Ethic Committees and regulatory authorities are granted direct access to the subjects medical records without violating subject confidentiality, and to the extent permitted by applicable regulations. The subject should be informed that by signing the informed consent form, the subject authorises such access.

The patient must be informed that biological samples taken within the framework of the study are anonymised and stored at a central location. Sample may be shipped from one country to another for storage and analysis. These samples are to be used for the scientific objectives described in this protocol or for future studies for which a separate information and consent form has to be signed off by the patients, excluding any commercial exploitation.

The patient signs and dates the informed consent. The informed consent is dated and countersigned by the investigator or a delegated person who explained the trial. The investigator or his delegate also inscribes his complete address and telephone number on the consent form. The number of informed consent signed depends on national recommandations. A copy of the signed informed consent is given to the patient.

Signed informed consents are retained by the investigator and made available (for review only) to the study monitor, auditor and inspector, upon request. An anonymised copy of the signed consent form is provided to the sponsor or its delegate.

7.2 Screening visit (W-6 to W-1)

A screening assessment for eligibility is performed between week -6 and week -1.
If patients with acute opportunistic infections are screened, the screening period should not exceed 2 weeks (screening visit between week -2 and week -1).

Signed and written informed consent for the trial must be obtained at latest at the screening visit and prior to the initiation of any study related intervention.

Screening procedures include:

- demographic information: date of birth, sex, ethnic group, country of birth
- history of HIV-associated conditions: mode of infection, CD4 nadir, date of first positive HIV-1 test, date of last HIV-1 negative test (if known), CDC stage
- documentation of historical genotype testing results, if available
- relevant medical history
- current medical conditions
- current medication
- recording of contraceptive methods in women
- assessment of inclusion and non-inclusion criteria
- complete physical examination including height and body weight (nude or light clothing only), heart rate and blood pressure
- blood sample for local laboratory assessments
  laboratory assessments:
  - plasma HIV-1 RNA
  - HIV-1 genotypic resistance profile (if not previously available. Original test results of previous genotypic resistance profile must be available)
  - HIV-1 subtype (if not previously available)
  - $CD4^+$(mandatory even if CD4 ≤ 500/mm^3 on a sample taken within 3 months before screening)
  - hepatitis serology: HBs Ag; HCV antibodies (if not previously available or if last assessment > 12 months before screening and HCV Ab negative, HBs Ab and HBc Ab negative)
  - haematology: complete blood count, plateletsclinical chemistry: creatinine, alkaline phosphatase, ALAT, ASAT, albumin
  - coagulation: prothrombin time (INR)
  - pregnancy test (serum beta-HCG) in all women of child bearing potential
- estimation of glomerular filtration rate (see Cockcroft & Gault equation in appendix 7)

7.3 Randomisation and treatment allocation

Staff from the clinical site sends a randomisation request to the responsible regional CTU when results of screening assessment are available and at the latest 7 days prior to W00.

Results of genotypic testing should be available before randomisation. However, if the patient presents with an acute opportunistic infection requiring urgent initiation of antiretroviral therapy, the patient may be enrolled and start trial treatment even if the results

of the genotype are still pending. In this case, conditions of one of the following two scenarios must be fulfilled, to enrol the patient in the trial:

- The patient has an acute opportunistic infection requiring urgent initiation of antiretroviral therapy (as judged by the investigator) and there is no evidence of any major IAS-USA mutation conferring resistance to one or more of reverse transcriptase or protease inhibitors on a historical genotype.
- The patient has an acute opportunistic infection requiring urgent initiation of antiretroviral therapy (as judged by the investigator), there is no historical genotype and screening genotyping is underway, but results are not yet available.

If urgent treatment initiation is required, the regional CTU should be contacted to arrange randomisation within due time.

The randomisation of patients is performed by the regional CTU after verification of the eligibility criteria. A centralised web-based computer program that guarantees process safety and traceability is used for randomisation. A written SOP describes the randomisation process.

Patients meeting the eligibility criteria are randomly allocated in a 1:1 ratio at trial entry to start one of the following open-label regimens over at least 96 weeks: darunavir/r + tenofovir/emtricitabine or darunavir/r + raltegravir.

Randomisation is centralised and stratified according to the following factors:

- Country
- participation in the “Viral and immunologic dynamics and inflammation sub-study”

The randomisation list is established by the study statistician prior to the start of the study.

Randomisation practicalities

The investigator enters the data collected at the screening visit onto the case record form and promptly sends the completed form and an anonymised copy of the signed consent form by fax to the regional designated CTU for eligibility check. The CTU then confirms eligibility and communicates the allocated trial regimen to the study site between three and one days before the scheduled baseline visit (W00).

RANDOMISATIONS

Tel: 0000000 Mon - Fri, 09:00 – 17:00)
Fax: 0000000

Randomisation and SAE notification numbers are specific to each regional CTU. Telephone and fax numbers to be inserted by regional CTU

7.4 Baseline visit (W00)

All patients participating in the trial should also be asked to participate in the pharmacogenomics study by signing a separate informed consent (see appendix 9). This should ideally be proposed at the baseline visit, but is also possible at any other visit during the trial.

At the baseline visit (W00), the following procedures are performed:

- medical history (new clinical and adverse events) since the last visit
- cardiovascular risk assessment: risk factors including smoking and alcohol history, family history
- medication history since the last visit
- recording of contraceptive methods in women
- complete physical examination (when clinically indicated), blood pressure + morphologic evaluation, including measurement of weight, hip and waist circumference
- electrocardiogram
- self-completed questionnaire on patients' reported outcomes (quality of life)
- in Italy only: recording of detailed pharmacoeconomic data
- blood sample for local laboratory assessments (recording of time and date of intake of the last meal and of blood draw)

  laboratory assessments:
  - plasma HIV-1 RNA
  - $CD4^+$ and $CD8^+$ T-cells
  - haematology: complete blood count, platelets
  - clinical chemistry (<u>patient has to be fasting for at least 10 hours prior to blood draw</u>): creatinine, sodium, potassium, phosphate, calcium, total and indirect bilirubin, alkaline phosphatase, GGT, ALAT, ASAT, lipase, CK, urea, albumin, bicarbonate, uric acid, glucose, triglycerides, cholesterol (total, HDL, LDL)
- blood samples for biobank: 3 EDTA vials (7 ml per vial)
  - preparation of 5 aliquots of 1 ml of plasma for storage
  - preparation of 4 aliquots of 1 ml full blood for storage
- blood sample for storage and centralised pharmacogenomic analysis <u>(only in patients having signed the separate informed consent)</u>: Sampling of one EDTA vial (1.5 ml) and preparation of 1 aliquot of 1.5 ml full blood for storage. (Handling and shipment are described in a specific SOP).
- urine sample:
  - dipstick assessment of glucosuria, proteinuria, leukocyturia, erythrocyturia and pH
  - protein/creatinine ratio, phosphate, uric acid, glucose

  - pregnancy test (urine beta-HCG) in all women of child bearing potential
  - preparation of 4 aliquots of 1 ml urine for biobank storage
- estimation of glomerular filtration rate (see Cockcroft & Gault equation in appendix 7)
- study drug dispensation
- scheduling of visit W02

Patients should start their trial treatment at latest on the day after the baseline visit.

The patient should be informed that he/ she should take all dispensed medication and that only empty containers (drug bottles) and expired study drug should be brought back during the trial. Unused study drug should only be returned to the study centre at the end of the trial in order to allow for drug accountability

Patients participating in the sub-studies may have additional blood and urine sampling or other assessments at this visit (see appendices 15-17).

Patients participating in the Viral and Immunologic Dynamics and Inflammation sub-study have additional blood sampling at Day 2-3 and at W01 (see appendix 15).

7.5 Follow-up visits

7.5.1 Visit W02

Visit W02 is performed between 12 and 16 days after the baseline visit with the main objective to assess the tolerance of the study drugs.

Procedures at visit W02 include:

- medical history and adverse events since the last visit
- medication history since the last visit
- recording of contraceptive methods in women
- complete physical examination (when clinically indicated) + measurement of weight and blood pressure
- blood sample for local laboratory assessments (recording of time and date of last meal, of last drug intake and of blood draw).

  laboratory assessments:
  - haematology: complete blood count, platelets
  - clinical chemistry (<u>patient has to be fasting for at least 10 hours prior to blood draw</u>): creatinine, phosphate, ALAT, ASAT, total and indirect bilirubin, CK, triglycerides, cholesterol (total, HDL, LDL)
- blood samples for biobank: 3 EDTA vials (7ml per vial)
  - preparation of 4 aliquots of 1 ml of plasma for storage

  - o preparation of 4 aliquots of 1 ml full blood for storage
- urine sample:
  - o dipstick assessment of glucosuria, proteinuria and erythrocyturia
- estimation of glomerular filtration rate (see Cockcroft & Gault equation in appendix 7)
- scheduling of visit W04

Patients participating in the Viral and Immunologic Dynamics and Inflammation sub-study have additional blood sampling at this visit and at W03 (see appendix 15).

7.5.2 Visit W04

Visit W04 is performed between 26 and 30 days after the baseline visit.
Patients should not take their trial drugs on the morning of the day of the visit (due to drug concentration assessment at W04; drug intake must be at least 5 hours prior to blood draw).

Procedures at visit W04 include:

- medical history and adverse events since the last visit
- medication history since the last visit
- recording of contraceptive methods in women
- recording of in-patient care resource utilisation (type and duration of hospital stays)
- complete physical examination (when clinically indicated), blood pressure and heart rate + morphologic evaluation, including measurement of weight, hip and waist circumference
- self-completed patients' reported outcomes questionnaire (quality of life, adherence)
- blood sample for local laboratory assessments (recording of time and date of intake of the last meal, of last drug intake and of blood draw)
  laboratory assessments:
  - o plasma HIV-1 RNA
  - o $CD4^+$ T-cells
  - o haematology: complete blood count, platelets
  - o clinical chemistry (patient has to be fasting for at least 10 hours prior to blood draw): creatinine, sodium, potassium, phosphate, calcium, total and indirect bilirubin, alkaline phosphatase, GGT, ALAT, ASAT, lipase, CK, urea, albumin, bicarbonate, uric acid, triglycerides, cholesterol (total, HDL, LDL)
- blood samples for biobank: 3 EDTA vials (7 ml per vial)
  - o preparation of 4 aliquots of 1 ml of plasma for storage
  - o preparation of 4 aliquots of 1 ml full blood for storage

- blood sample for centralised drug concentration analysis: Sampling of 10 ml in EDTA vials (one 10 ml vial or two 7 ml vials) and preparation of 4 aliquots of 1 ml of plasma for storage. Handling and shipment are described in a specific SOP.)
- urine sample:
  - dipstick assessment of glucosuria, proteinuria, leukocyturia, erythrocyturia and pH
  - protein/creatinine ratio, phosphate, uric acid, glucose
  - pregnancy test (urine beta-HCG) in all women of child bearing potential
  - preparation of 4 aliquots of 1 ml urine for biobank storage
- estimation of glomerular filtration rate (see Cockcroft & Gault equation in appendix 7)
- counting of returned drugs
- study drug dispensation
- scheduling of visit W08

Patients participating in the Viral and Immunologic Dynamics and Inflammation sub-study have additional blood sampling at this visit (see appendix 15).

7.5.3 Visit W08

Visit W08 is performed between 7 and 9 weeks after the baseline visit.

Procedures at visit W08 include:

- medical history and adverse events since the last visit
- medication history since the last visit
- recording of contraceptive methods in women
- recording of in-patient care resource utilisation (type and duration of hospital stays)
- complete physical examination (when clinically indicated), blood pressure + morphologic evaluation, including measurement of weight, hip and waist circumference
- blood sample for local laboratory assessments (recording of time and date of intake of the last meal, of last drug intake and of blood draw).
  laboratory assessments:
  - plasma HIV-1 RNA
  - $CD4^+$ T-cells
  - haematology: complete blood count, platelets
  - clinical chemistry (patient has to be fasting for at least 10 hours prior to blood draw): creatinine, sodium, phosphate, calcium, total and indirect bilirubin, alkaline phosphatase, GGT, ALAT, ASAT, CK, triglycerides, cholesterol (total, HDL, LDL)
- blood samples for biobank: 3 EDTA vials (7ml per vial)

   - preparation of 4 aliquots of 1 ml of plasma for storage
   - preparation of 4 aliquots of 1 ml full blood for storage
- urine sample:
   - dipstick assessment of glucosuria, proteinuria and erythrocyturia
   - pregnancy test (urine beta-HCG) in all women of child bearing potential
- estimation of glomerular filtration rate (see Cockcroft & Gault equation in appendix 7)
- counting of returned drugs
- study drug dispensation
- scheduling of visit W12

Patients participating in the Viral and Immunologic Dynamics and Inflammation sub-study have additional blood sampling at this visit (see appendix 15).

7.5.4 Visit W12

Visit W12 is performed between 11 and 13 weeks after the baseline visit.

Procedures at visit W12 include:

- medical history and adverse events since the last visit
- medication history since the last visit
- recording of contraceptive methods in women
- recording of in-patient care resource utilisation (type and duration of hospital stays)
- complete physical examination (when clinically indicated), blood pressure + morphologic evaluation, including measurement of weight, hip and waist circumference
- self-completed patients' reported outcomes questionnaire (quality of life, adherence)
- blood sample for local laboratory assessments (recording of time and date of intake of the last meal, of last drug intake and of blood draw).
  laboratory assessments:
   - plasma HIV-1 RNA
   - $CD4^+$ T-cells
   - haematology: complete blood count, platelets
   - clinical chemistry (<u>patient has to be fasting for at least 10 hours prior to blood draw</u>): creatinine, sodium, potassium, phosphate, calcium, total and indirect bilirubin, alkaline phosphatase, GGT, ALAT, ASAT, lipase, CK, urea, albumin, bicarbonate, uric acid, glucose, triglycerides, cholesterol (total, HDL, LDL)
- blood samples for biobank: 3 EDTA vials (7ml per vial)
   - preparation of 4 aliquots of 1 ml of plasma for storage

    - preparation of 4 aliquots of 1 ml full blood for storage
- urine sample:
    - dipstick assessment of glucosuria, proteinuria, leukocyturia, erythrocyturia and pH
    - protein/creatinine ratio, phosphate, uric acid, glucose
    - pregnancy test (urine beta-HCG) in all women of child bearing potential
    - preparation of 4 aliquots of 1 ml urine for biobank storage
- estimation of glomerular filtration rate (see Cockcroft & Gault equation in appendix 7)
- counting of returned drugs
- study drug dispensation
- scheduling of visit W18

Patients participating in the Viral and Immunologic Dynamics and Inflammation sub-study have additional blood sampling at this visit (see appendix 15).

7.5.5 Visit W18

Visit W18 is performed between 17 and 19 weeks after the baseline visit. At this visit, only blood sampling is performed.

Procedures at visit W18 include:

- recording of contraceptive methods in women
- blood sample for local laboratory assessments (recording of time and date of intake of the last meal, of last drug intake and of blood draw).
  laboratory assessments:
    - plasma HIV-1 RNA
    - $CD4^+$ T-cells
    - haematology: complete blood count, platelets
    - clinical chemistry (<u>patient has to be fasting for at least 10 hours prior to blood draw</u>): creatinine, total and indirect bilirubin, ALAT, ASAT
- blood samples for biobank: 3 EDTA vials (7ml per vial)
    - preparation of 4 aliquots of 1 ml of plasma for storage
    - preparation of 4 aliquots of 1 ml full blood for storage
- urine sample:
    - dipstick assessment of glucosuria, proteinuria and erythrocyturia
    - pregnancy test (urine beta-HCG) in all women of child bearing potential
- estimation of glomerular filtration rate (see Cockcroft & Gault equation in appendix 7)
- scheduling of visit W24

7.5.6 Visit W24

Visit W24 is performed between 23 and 25 weeks after the baseline visit.
Patients should not take their trial drugs on the morning of the day of the visit (due to drug concentration assessment at W24; drug intake must be at least 5 hours prior to blood draw).

All patients participating in the trial should also be proposed to participate in the pharmacogenomics study, by signing a separate informed consent. This is possible at any visit during the trial.

Procedures at visit W24 include:

- medical history and adverse events since the last visit
- cardiovascular risk assessment
- medication history since the last visit
- recording of contraceptive methods in women
- recording of in-patient care resource utilisation (type and duration of hospital stays)
- complete physical examination (when clinically indicated), blood pressure and heart rate + morphologic evaluation, including measurement of weight, hip and waist circumference
- self-completion of the patients' reported outcomes questionnaire (quality of life, adherence)
- in Italy only: recording of detailed pharmacoeconomic data
- blood sample for local laboratory assessments (recording of time and date of intake of the last meal, of last drug intake and of blood draw).
  laboratory assessments:
  - plasma HIV-1 RNA
  - $CD4^+$ and $CD8^+$ T-cells
  - haematology: complete blood count, platelets
  - clinical chemistry (patient has to be fasting for at least 10 hours prior to blood draw): creatinine, sodium, phosphate, calcium, total and indirect bilirubin, alkaline phosphatase, GGT, ALAT, ASAT, lipase, CK, glucose, triglycerides, cholesterol (total, HDL, LDL)
- blood samples for biobank: 3 EDTA vials (7ml per vial)
  - preparation of 4 aliquots of 1 ml of plasma for storage
  - preparation of 4 aliquots of 1 ml full blood for storage
- blood sample for centralised drug concentration analysis: Sampling of 10 ml in EDTA vials (one 10 ml vial or two 7 ml vials) and preparation of 4 aliquots of 1 ml of plasma for storage. Handling and shipment are described in a specific SOP.)

- urine sample:
  - dipstick assessment of glucosuria, proteinuria and erythrocyturia
  - pregnancy test (urine beta-HCG) in all women of child bearing potential
- estimation of glomerular filtration rate (see Cockcroft & Gault equation in appendix 7)
- counting of returned drugs
- study drug dispensation
- scheduling of visit W32

Patients participating in the Viral and Immunologic Dynamics and Inflammation sub-study have additional blood sampling at this visit (see appendix 15).

7.5.7 Visit W32

Visit W32 is performed between 31 and 33 weeks after the baseline visit.

Procedures at visit W32 include:

- medical history and adverse events since the last visit
- medication history since the last visit
- recording of contraceptive methods in women
- recording of in-patient care resource utilisation (type and duration of hospital stays)
- complete physical examination (when clinically indicated) + measurement of weight and blood pressure
- blood sample for local laboratory assessments (recording of time and date of intake of the last meal, of last drug intake and of blood draw).
  laboratory assessments:
  - plasma HIV-1 RNA
  - $CD4^+$ T-cells
  - haematology: complete blood count, platelets
  - clinical chemistry (<u>patient has to be fasting for at least 10 hours prior to blood draw</u>): creatinine, sodium, phosphate, calcium, total and indirect bilirubin, alkaline phosphatase, GGT, ALAT, ASAT, CK
- blood samples for biobank: 3 EDTA vials (7ml per vial)
  - preparation of 4 aliquots of 1 ml of plasma for storage
  - preparation of 4 aliquots of 1 ml full blood for storage
- urine sample:
  - dipstick assessment of glucosuria, proteinuria and erythrocyturia
  - pregnancy test (urine beta-HCG) in all women of child bearing potential

- estimation of glomerular filtration rate (see Cockcroft & Gault equation in appendix 7)
- counting of returned drugs
- study drug dispensation
- scheduling of visit W48

If HIV-1 RNA is ≥ 50 copies/ml at visit W32, a retest has to be performed as soon as possible, and in any case before week 36.

If HIV-1 RNA is ≥ 500 copies/ml at any time after visit W32, HIV-1 genotype is to be assessed in the local laboratory. In addition, 1 ml of plasma is to be stored and frozen at that timepoint (biobank).

If HIV-1 RNA assessment is missing at visit W32 for any reason, the patient should be asked to come for a blood draw to perform plasma HIV-1 RNA measurement as soon as possible and no later than W40.

7.5.8 Visit W48

All patients participating in the trial should also be proposed to participate in the pharmacogenomics study, by signing a separate informed consent (see appendix 3). This is possible at any visit during the trial.

Visit W48 is performed between 47 and 49 weeks after the baseline visit.

Procedures at visit W48 include:

- medical history and adverse events since the last visit
- cardiovascular risk assessment
- medication history since the last visit
- recording of contraceptive methods in women
- recording of in-patient care resource utilisation (type and duration of hospital stays)
- complete physical examination, blood pressure and heart rate + morphologic evaluation, including measurement of weight, hip and waist circumference
- electrocardiogram
- completion of the self-completed patients' reported outcomes questionnaires (quality of life, adherence)
- in Italy only: recording of detailed pharmacoeconomic data
- blood sample for local laboratory assessments (recording of time and date of intake of the last meal, of last drug intake and of blood draw).

  laboratory assessments:
  - plasma HIV-1 RNA
  - $CD4^+$ and $CD8^+$ T-cells

  - haematology: complete blood count, platelets
  - clinical chemistry (patient has to be fasting for at least 10 hours prior to blood draw): creatinine, sodium, potassium, phosphate, calcium, total and indirect bilirubin, alkaline phosphatase, GGT, ALAT, ASAT, lipase, CK, urea, albumin, bicarbonate, uric acid, glucose, triglycerides, cholesterol (total, HDL, LDL)
- blood samples for biobank: 3 EDTA vials (7ml per vial)
  - preparation of 4 aliquots of 1 ml of plasma for storage
  - preparation of 4 aliquots of 1 ml full blood for storage
- urine sample:
  - dipstick assessment of glucosuria, proteinuria, leukocyturia, erythrocyturia and pH
  - protein/creatinine ratio, phosphate, uric acid, glucose
  - pregnancy test (urine beta-HCG) in all women of child bearing potential
  - preparation of 4 aliquots of 1 ml urine for storage
- estimation of glomerular filtration rate (see Cockcroft & Gault equation in appendix 7)
- counting of returned drugs
- study drug dispensation
- scheduling of visit W64

Patients participating in the sub-studies may have additional blood sampling or other assessments at this visit (see appendices 15-17).

7.5.9 Visit W64

Visit W64 is performed between 62 and 66 weeks after the baseline visit.

Procedures at visit W64 include:

- medical history and adverse events since the last visit
- medication history since the last visit
- recording of contraceptive methods in women
- recording of in-patient care resource utilisation (type and duration of hospital stays)
- complete physical examination (when clinically indicated) + measurement of weight and blood pressure
- in Italy only: recording of detailed pharmacoeconomic data
- blood sample for local laboratory assessments (recording of time and date of intake of the last meal, of last drug intake and of blood draw).

  laboratory assessments:
  - plasma HIV-1 RNA

  - $CD4^+$ T-cells
  - haematology: complete blood count, platelets
  - clinical chemistry (patient has to be fasting for at least 10 hours prior to blood draw): creatinine, sodium, phosphate, calcium, total and indirect bilirubin, alkaline phosphatase, GGT, ALAT, ASAT, CK
- blood samples for biobank: 3 EDTA vials (7ml per vial)
  - preparation of 4 aliquots of 1 ml of plasma for storage
  - preparation of 4 aliquots of 1 ml full blood for storage
- urine sample:
  - dipstick assessment of glucosuria, proteinuria and erythrocyturia
  - pregnancy test (urine beta-HCG) in all women of child bearing potential
- estimation of glomerular filtration rate (see Cockcroft & Gault equation in appendix 7)
- counting of returned drugs
- study drug dispensation
- scheduling of visit W80

7.5.10 Visit W80

Visit W80 is performed between 78 and 82 weeks after the baseline visit.

Procedures at visit W80 include:

- medical history and adverse events since the last visit
- medication history since the last visit
- recording of contraceptive methods in women
- recording of in-patient care resource utilisation (type and duration of hospital stays)
- complete physical examination (when clinically indicated) + measurement of weight and blood pressure
- blood sample for local laboratory assessments (recording of time and date of intake of the last meal, of last drug intake and of blood draw).
  laboratory assessments:
  - plasma HIV-1 RNA
  - $CD4^+$ and $CD8^+$ T-cells
  - haematology: complete blood count, platelets
  - clinical chemistry (patient has to be fasting for at least 10 hours prior to blood draw): creatinine, sodium, phosphate, calcium, total and indirect bilirubin, alkaline phosphatase, GGT, ALAT, ASAT, CK

- blood samples for biobank: 3 EDTA vials (7ml per vial)
  - preparation of 4 aliquots of 1 ml of plasma for storage
  - preparation of 4 aliquots of 1 ml full blood for storage
- urine sample:
  - dipstick assessment of glucosuria, proteinuria and erythrocyturia
  - pregnancy test (urine beta-HCG) in all women of child bearing potential
- estimation of glomerular filtration rate (see Cockcroft & Gault equation in appendix 7)
- counting of returned drugs
- study drug dispensation
- scheduling of visit W96

7.5.11 Visit W96

All patients not having already consented to participate in the pharmacogenomics study should be reproposed to participate by signing a separate informed consent (appendix 3).

Visit W96 is performed between 94 and 98 weeks after the baseline visit.

Procedures at visit W96 include:

- medical history and adverse events since the last visit
- cardiovascular risk assessment
- medication history since the last visit
- recording of contraceptive methods in women
- recording of in-patient care resource utilisation (type and duration of hospital stays)
- complete physical examination, blood pressure + morphologic evaluation, including measurement of weight, hip and waist circumference
- patients' reported outcomes self-questionnaire (quality of life, adherence)
- in Italy only: recording of detailed pharmacoeconomic data
- electrocardiogram
- blood sample for local laboratory assessments (recording of time and date of intake of the last meal, of last drug intake and of blood draw).
  laboratory assessments:
  - plasma HIV-1 RNA
  - $CD4^+$ and $CD8^+$ T-cells
  - haematology: complete blood count, platelets
  - clinical chemistry (<u>patient has to be fasting for at least 10 hours prior to blood draw</u>): creatinine, sodium, potassium, phosphate, calcium, total and indirect bilirubin,

alkaline phosphatase, GGT, ALAT, ASAT, lipase, CK, urea, albumin, bicarbonate, uric acid, glucose, triglycerides, cholesterol (total, HDL, LDL)

- blood samples for biobank: 3 EDTA vials (7ml per vial)
    - preparation of 4 aliquots of 1 ml of plasma for storage
    - preparation of 4 aliquots of 1 ml full blood for storage
- urine sample:
    - dipstick assessment of glucosuria, proteinuria, leukocyturia, erythrocyturia and pH
    - protein/creatinine ratio, phosphate, uric acid, glucose
    - pregnancy test (urine beta-HCG) in all women of child bearing potential
    - preparation of 4 aliquots of 1 ml urine for storage
- estimation of glomerular filtration rate (see Cockcroft & Gault equation in appendix 7)
- counting of returned drugs
- study drug dispensation for those patients having not reached the end of the trial (defined as the last patient's W96 visit)
- scheduling of next visit for those patients having not reached the end of the trial (defined as the last patient's W96 visit)

Patients participating in the sub-studies may have additional blood sampling or other assessments at this visit (see appendices 15-17).

7.6 Visits after W96

All patients remain in the trial until the last patient enrolled has completed the W96 visit. Follow-up visits after W96 should be scheduled every three to four months and procedures performed according to standard of care. At every follow-up visit, the following assessments and procedures are performed:

- medical history and adverse events since the last visit
- medication history since the last visit
- recording of contraceptive methods in women
- blood sample for local laboratory assessments

  laboratory assessments:
    - plasma HIV-1 RNA
    - $CD4^+$ T-cells
    - complete blood count
    - Other laboratory assessments as standard of care
- study drug dispensation for those patients having not reached the end of the trial (defined as the last patient's W96 visit)

Data collected at follow-up visits after W96 need to be recorded on the case report forms. Visits after W96 are identified by months of follow-up (FU-M) and not weeks.

7.7 Assessment of safety

The following procedures aim at assessing safety during the trial (see paragraph 7.5 for schedule of assessments):

- Recording of current medical conditions, HIV-associated conditions, AEs, SAEs and SUSARs (see chapter 9 for reporting of AEs, SAEs, and SUSARs)
- Medication reporting
- Complete physical examination: vital signs, body weight, general appearance, eyes, ears/ nose/throat, cardiovascular, respiratory, gastrointestinal, neurological, musculoskeletal, skin, other. Weight is measured at each visit, the patient being nude or wearing underwear only). Systolic and diastolic blood pressure is also assessed at each visit. Physical examination is targeted according to presenting signs and symptoms.
- Measurement of hip and waist circumference
- Laboratory assessments (hematology, clinical chemistry, urine analysis, estimated glomerular filtration rate)

7.8 Assessment of efficacy

The following procedures aim at assessing efficacy during the trial (see paragraph 7.5 for schedule of assessments):

- Laboratory assessments:
    - o plasma HIV-1 RNA
    - o $CD4^+$ and $CD8^+$ T-cells
- Recording of AIDS-defining events, non-AIDS severe events, and death

All patients having achieved a plasma viral load < 50 copies/ml at any time and having a subsequent viral load ≥ 50 copies/ml should be retested from 10 days to 4 weeks.

7.9 Assessment of treatments

7.9.1 Antiretroviral treatment

At all study visits, the following details are recorded for any antiretroviral drugs, including the study drugs:

- commercial name of the product
- formulation
- dosage per unit
- prescribed dosage
- start date of this prescribed dosage
- stop date of this prescribed dosage
- reason for discontinuation

7.9.2 Concomitant treatment

At all study visits, the following details are recorded for all concomitant medications:

- commercial name of the product
- formulation
- dosage per unit
- prescribed dosage
- indication
- start date
- stop date

7.10 Assessment of compliance and adherence

Patient's compliance to treatment is assessed by the following procedures during the trial:

- self-questionnaire (see paragraph 7.5 for schedule of assessments)
- counting of returned drugs at the end of trial treatment

Patients' adherence to the trial is assessed by recording loss-to-follow-up, missed visits, and the reason for withdrawals. Effort should be made to contact patients who do not come to scheduled study visit and to obtain at least information on vital status.

7.11 Other assessments

The following ancillary assessments are performed in all patients:

- Blood sampling for Pharmacogenetics study (in consenting patients)
- Blood sampling for Population pharmacokinetics
- Blood sampling for Viral sub-type study
- Blood sampling for Renal function study
- Self-questionnaire for Quality of life study
- Recording of in-patient care resource utilisation for Pharmacoeconomics study

For the full assessment items, please refer to appendices 9-14.

7.12 Study regimen termination

Study regimen termination is defined by the definite discontinuation of any component of the initially allocated study regimen for any reason. The decision on regimen discontinuation should be taken based on clinical decision criteria and standard of care, and not on the protocol endpoints. The reason for regimen discontinuation and the date of discontinuation must be recorded in case record form.

The patient should receive appropriate 2nd line antiretroviral treatment after trial regimen discontinuation, based on current guidelines, patient's history and condition as well as co-medications.

Study regimen modifications should not lead to patient withdrawal from the trial: Patients discontinuing their study regimen have to be followed-up according to the scheduled trial visits until the end of the trial and all data should be collected (under the condition that informed consent has not been withdrawn).

If a patient wishes to discontinue trial treatment at any moment, investigators should explain the importance of remaining on trial follow-up, or - failing this - of allowing routine follow-up data to be used for trial purposes.

Regimen termination guidelines

Decision to discontinue a patient from the study regimen may be considered in the following circumstances:

- Insufficient virologic response:
    - HIV-1 RNA reduction < 1 log10 copies/ml at W04, W08, W12 or W18, or HIV-1 RNA ≥ 400 copies/ml at W24

      Any decision should only be taken after confirmation of the HIV-1 RNA level and after discussion with the regional CTU.
    - HIV-1 RNA ≥ 50 copies/ml by W48 (if suppression of HIV-1 RNA < 50 copies/ml never achieved).
- Confirmed rebound in HIV-1 RNA ≥ 50 copies/ml after initial suppression below 50 copies/ml.

  All patients having achieved a plasma viral load < 50 copies/ml at any time and having a subsequent viral load ≥ 50 copies/ml should be retested from 10 days to 4 weeks.
- Disease progression whilst on therapy.
- Withdrawal of patient consent.
- Unacceptable toxicity.
- Intercurrent illness which prevents further treatment, such as the need to start concomitant treatment with medication that is not compatible with the use of the study medications.
- Any change in the patient's condition, such as pregnancy, which justifies the discontinuation of treatment in the clinician's opinion.

7.12.1 Management in the case of insufficient virologic response

- Insufficient virologic response before W32
  Insufficient virologic response before W32 is defined as HIV-1 RNA reduction < 1 log10 copies/ml at W04, W08, W12 or W18, or HIV-1 RNA ≥ 400 copies/ml at W24.

  In that case, adherence problems should be addressed and HIV-1 RNA levels confirmed. Any decision to modify the initial randomised regimen should only be taken after discussion between the investigator and the regional CTU.

- Failure to achieve a viral load < 50 copies/ml by W48
  If by W48 the patient has never achieved HIV-1 RNA < 50 copies/ml, either because of high viral load at inclusion or because of adherence problems, which should have been addressed, regimen change is strongly recommended.
  In patients with HIV-1 RNA ≥ 50 copies/ml at W48, HIV-1 RNA level should be retested from 10 days to 4 weeks.

7.12.2 Management in the case of protocol-defined virologic failure

Protocol definition of confirmed virologic failure is occurrence of an HIV-1 RNA level ≥ 50 copies/ml in two consecutive measurements, the second one to be done from 10 days to 4 weeks at the latest, after virologic suppression (HIV-1 RNA < 50 copies/ml) at any time.

All patients having achieved a plasma viral load < 50 copies/ml at any time and having a subsequent viral load ≥ 50 copies/ml should be retested at the local laboratory from 10 days to 4 weeks.

The December 1 2009 version of guidelines from the U.S. DHHS Panel on Antiretroviral Guidelines for Adults and Adolescents [3] states that low level viremia between 50 and 200 copies/ml of HIV-1 RNA should not necessarily be considered as virologic failure or a reason to change treatment. A similar position is adopted by the European EACS guidelines, version 5, November 2009 [2]. Therefore, patients with viral loads between 50 and 200 copies/ml should continue their treatment regimen, unless there is a clear indication to change therapy, such as documented resistance or if the low-level viremia is prolonged. In such cases, investigators must contact their regional CTU to discuss any virological concern before changing/modifying regimens. It is recommended to change the regimen when the viral load is confirmed ≥ 200 copies/ml.

At first HIV-1 RNA ≥ 500 copies/ml at any time after visit W32, HIV-1 genotype is to be assessed in the local laboratory. In addition, 1 ml of plasma is to be stored and frozen at that timepoint (biobank). This sample may later be sent to the central laboratory for centralised genotypic analysis (see specific SOP on sample handling and shipment).

In any case, change of therapy is left at the discretion of the treating clinician after careful assessment of failure reasons and consequences (genotypic resistance testing). The decision on regimen discontinuation should be taken based on clinical decision criteria and standard of care, and not on the protocol endpoints. The patients should be offered an active antiretroviral combination (see 7.12.5) and follow-up be continued until completion of the trial.

7.12.3 Management in the case of disease progression

In case of disease progression, either AIDS-defining events or serious non-AIDS events, the treating physician decides whether treatment changes are indicated.

7.12.4 Management in the case of treatment limiting adverse events

The investigator should consider temporarily withholding therapy if the severity of the adverse experience is Grade 3 or above and/or if clinically indicated. Such decision to interrupt therapy should take into account the patient's baseline laboratory values and any concomitant medication or clinical condition that could be contributory. At the discretion of the investigator, therapy may generally be reinitiated when laboratory abnormalities or clinical adverse events return to near normal or to baseline values. If the adverse event is considered serious or if exposure to the study drug(s) poses additional potential significant risk to the patient, rechallenge is not recommended.
Whenever study drugs are permanently interrupted, i.e. for > 1 month, the regional CTU should be immediately notified.

A dosing interval adjustment of tenofovir/emtricitabine is recommended in all patients with creatinine clearance 30-49 ml/min (see SmPC). Tenofovir/emtricitabine should be permanently discontinued if creatinine clearance is < 30 ml/min.

- Management of rashes
In case of rash grade 3-4 possibly related to study drugs, therapy should be immediately and permanently discontinued.
Rash grade 3:

- diffuse macular, maculopapular, or morbiliform rash with vesicles or limited number of bullae OR
- cutaneous rash/reaction with superficial ulcerations of mucous membrane limited to one site OR
- cutaneous rash/reaction with at least one of the following: elevation of AST and/or ALT>2 x the baseline value and at least >5 x ULN; fever (>38°C); eosinophils >1000/mm^3; serum sickness like reaction.

Rash grade 4:

- Extensive or generalised bullous lesions OR
- Stevens-Johnson syndrome (SJS) OR
- Ulceration of mucous membrane involving 2 or more distinct mucosal sites OR
- Toxic epidermal necrolysis (TEN)
- Cutaneous rash/reaction with eosinophilia and systemic symptoms (DRESS)

Grades 2 to 4 rashes are regularly validated by the Endpont Review Committee and reviewed by the Independent Data Monitoring Committee and the Trial Steering Committee.

All relevant details to categorise treatment-limiting adverse events including nature, type, severity, relation to study drugs, evolution, etc are recorded in the CRF.
Follow-up after drug discontinuation follows planned study visits, with an additional visit 4 weeks after drug discontinuation, with the main objective to assess evolution of the event that led to study drug termination.

7.12.5 Second line antiretroviral regimens

If the treatment allocated by randomisation is discontinued for virologic failure, disease progression or treatment-limiting adverse events, the patient should be offered a new antiretroviral regimen based on current guidelines after assessment of cause of discontinuation, and genotypic testing results in case of virologic failure.

7.13 Management in the case of pregnancy

In case of suspicion of pregnancy during the trial, a pregnancy test should be performed immediately. Pregnancy must be notified to the regional CTU.
If a female participant becomes pregnant, all study drugs that are not recommended during pregnancy based on the latest SmPC must be discontinued. Women should receive regimens that are authorised during pregnancy. For female patients requiring a change in study medication due to pregnancy, this should be done after discussion with the Trial Management Team (TMT). All patients should resume the initial randomised treatment within 6 weeks after delivery.
Follow-up of all patients continues until the end of the trial. All pregnancies are followed to the final outcome.

7.14 Management in the case of suspicion of Immune Reconstitution Syndrome

If Immune Reconstitution Syndrome (IRS) is suspected, a detailed assessment of its etiology should be conducted [37]. All data relevant to the IRS event need to be recorded in the CRF. All cases of IRS are reviewed by the Endpoint Review Committee.

7.15 Patient transfers

For patients moving from the area, every effort should be made for the patient to be followed-up at another participating trial centre and for this trial centre to take over responsibility for the patient. Access to the patient CRF must be provided to the new site. The patient may have to sign a new consent form at the new site, and until this occurs, the patient remains under the responsibility of the original centre.

7.16 Discontinuation and withdrawal

In consenting to the trial, patients are consenting to trial treatment, trial follow-up and data collection.
Particular situations, which may result in deviations from this stipulation, are detailed in the following chapters.

7.16.1 Premature discontinuation from the study

A patient prematurely discontinues from the study if:

- the patient withdraws informed consent (cf paragraph 7.16.2).
- the patient permanently refuses to come back to study visits
- the patient is lost to follow up (cf paragraph 7.16.3).
- the patient dies.

In case the patient discontinues the study prematurely, the end-of-study page of the CRF is completed.

7.16.2 Withdrawal of consent

Patients may voluntarily withdraw from the study for any reason at any time. When a patient withdraws his/her consent to participate in the study, no new information must be collected and recorded in the database after the date of withdrawal. Similarly, no samples must be collected after that date in the context of the research study.
Withdrawals of consent to participate in the study must be reported to the regional CTU as soon as possible (by fax and by letter). The investigator must document the date, reason and any answers given in response to the patient, in the patient's medical records.
If a patient explicitly states his/her wish not to contribute data to the study, the regional CTU should be informed in writing of the patient’s decision.
Patients withdrawing from the trial without stating this wish have previously consented to follow-up in the trial, and data up to this time can be included in the trial if it is anonymised.

7.16.3 Loss to follow-up

For patients who are lost to follow-up (i.e., those patients whose status is unclear because they fail to appear for study visits without stating an intention to withdraw), the investigator should show "due diligence" by documenting in the source documents steps taken to contact the patient, e.g. dates of telephone calls, registered letters, etc. If no notice about the patient’s status can be obtained the patient is considered lost to follow up.
The regional CTU should be informed as soon as possible (by fax and by letter) that a patient is lost to follow up.

7.17 Data collection

Staff at the clinical centres is responsible for:

- recording all relevant information in the clinical notes (see sections 7.2 – 7.16), and holding a record for each participant which includes the CRFs with any changes made signed and dated
- accurately completing the case record forms
- returning the completed forms in due time to the regional CTU
- notifying SAEs to the regional CTU promptly after becoming aware of the event

Copies of laboratory reports containing the results of routine haematology, chemical pathology and immunology must be kept in the clinical notes. A clinician of the clinical trial team must review, sign and date the laboratory report. In the event of an abnormality, an indication should be given whether or not action was taken.

CRFs are supplied by the regional CTU, on paper or electronic format.

The site investigator is responsible for overseeing that all data on the case report forms is recorded accurately and in a timely fashion.
If a paper CRF is used, corrections to entries are to be crossed out with a single line and the correct value written above. The corrected entry is then initialed and dated by the person authorised to make corrections in the case report forms, including changes made before the form is returned to the regional CTU.

Copies of CRFs and clinical notes should be kept in a secure location for at least 10-15 years after the clinical trial has ended (as per local regulations).

7.18 Trial close out

The trial is considered closed after one year after last patient's last visit and after formal notification by the sponsor.

8. SAFETY REPORTING

The definitions of the EU Directive 2001/20/EC Article 2 based on ICH GCP apply in this trial protocol.

Figure 2: Safety Reporting Flowchart

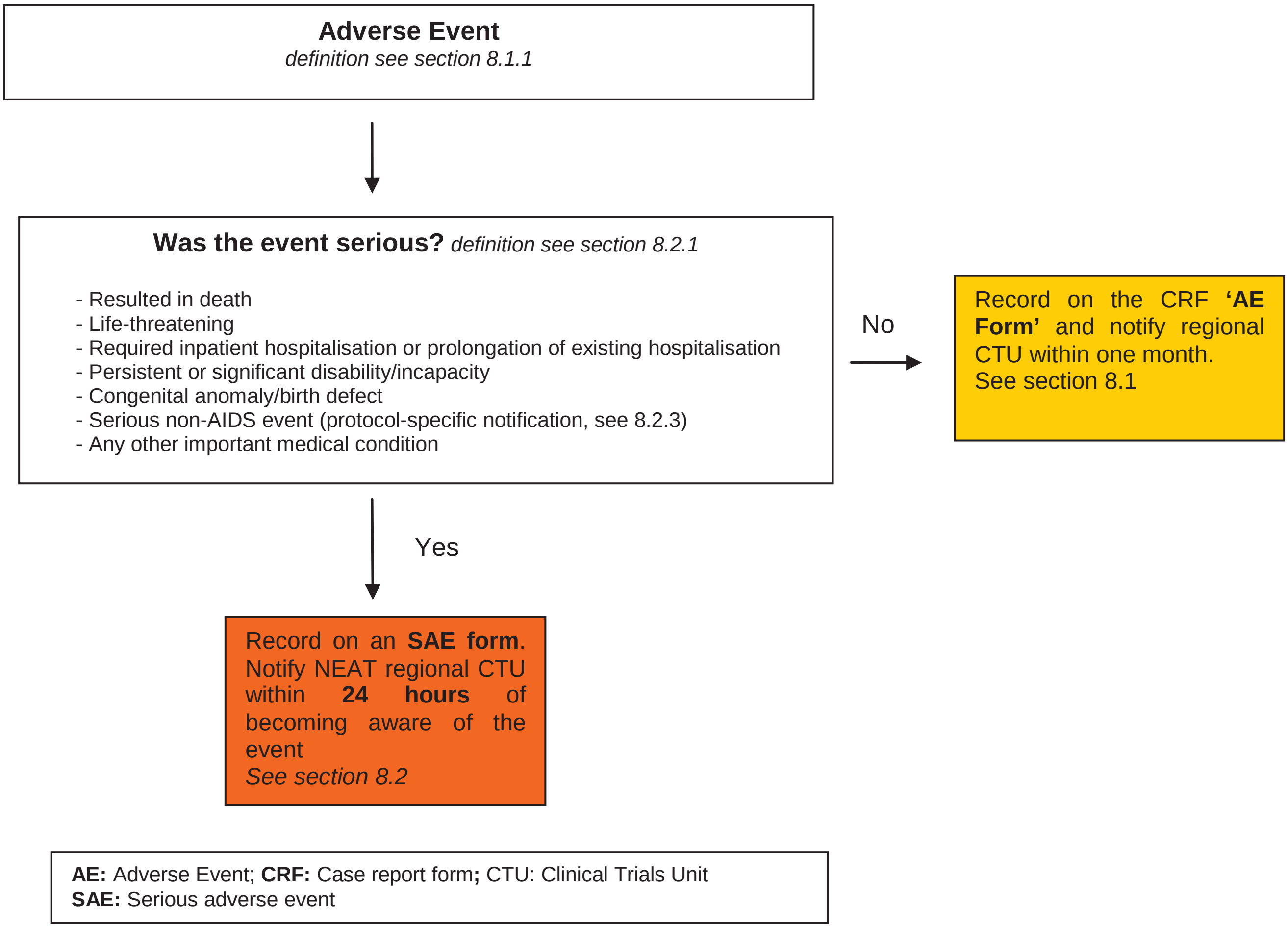

8.1 Adverse Events

8.1.1 Definitions

Table 2: Definitions of Adverse Events

**Term**	Definition
**Adverse Event (AE)**	Any untoward medical occurrence in a patient or clinical trial subject to whom a medicinal product has been administered including occurrences which are not necessarily caused by or related to that product.
**Adverse Reaction (AR)**	Any untoward and unintended response to an investigational medicinal product related to any dose administered.
**Unexpected Adverse Reaction (UAR)**	An adverse reaction, the nature or severity of which is not consistent with the information about the medicinal product in question set out in the summary of product characteristics (or Investigator brochure) for that product.

Adverse events are classified according to the ANRS scale to grade the severity of adverse events in adults, Version n° 1.0, 4 November 2008 (see Appendix 6).

8.1.2 Clarifications and Exceptions

Followings events are considered as AEs that should be documented on the Adverse Event form of the CRF:

- Pre-existing events, which increase in frequency or severity, or change in nature during or as a consequence of use of a drug in human clinical trials
- Clinically significant laboratory abnormalities are considered as adverse events.
- Any overdose whether or not associated with an adverse experience. An overdose is defined as any dose exceeding the maximum recommended dose according to the prescribing information.

ADVERSE EVENTS DO NOT INCLUDE:

- Medical or surgical procedures (e.g., surgery, endoscopy, tooth extraction, transfusion). The condition that leads to the procedure is an AE.
- Pre-existing diseases or conditions present or detected prior to start of study drug administration that do not worsen
- Situations where an untoward medical occurrence has not occurred (e.g. hospitalisation for elective surgery if known prior to study start, social and/or convenience admissions)

8.2 Serious Adverse Events

8.2.1 Definitions

Table 3: Definition of Serious Adverse Events

Term	Definition
**Serious Adverse Event (SAE) or Serious Adverse Reaction (SAR) or Suspected Unexpected Serious Adverse Reaction (SUSAR)**	Respectively any adverse event, adverse reaction or unexpected adverse reaction that:<br>• results in death (1)<br>• is life-threatening (patient is at immediate risk of death) (2)<br>• requires hospitalisation or prolongation of existing hospitalisation (excluding those for study therapy) (3)<br>• results in persistent or significant disability or incapacity<br>• consists of a congenital anomaly or birth defect<br>• other: emergency admissions and other medically significant events that may not result in death, be immediately life-threatening, or require hospitalisation, may be considered an SAE when, based upon appropriate medical judgment, they may jeopardise the patient and may require medical or surgical intervention to prevent one of the outcomes listed in this definition. (4)

An SAE has to be reported if it occurs from the date of consent form signature to 30 days after the last follow-up visit, and indefinitely in case of SAR or SUSAR (see 8.3.1).
"Occurring at any dose" does not imply that the patient is receiving study drug at the time of the event. Dosing may have been given as treatment cycles or interrupted temporarily prior to the onset of the SAE, but may have contributed to the event.

(1) Deaths must be reported for patients on study as outcome of an adverse event, and not as an adverse event itself if the cause is known. If the cause is unknown, the death should be reported as "unknown cause of death". In reports of death due to "Disease Progression", where no other information is provided, the death is assumed to have resulted from progression of the disease being treated with the study drug(s).

(2) "Life-threatening," means that the patient was **at immediate risk** of death from the event as it occurred.

(3) "Inpatient hospitalisation" means that the patient has been formally admitted to a hospital for medical reasons, for any length of time. This may or may not be overnight. It does not include presentation and care within an emergency department, unless the event is judged as an "other important medical event" as per the definition of serious described above.
Complications that occur during hospitalisations are AEs but are not SAEs per se unless they meet one or more of the criteria for being a serious adverse event.

(4) Medical judgement should be exercised in deciding whether an AE is serious in other situations. Important AE/ARs that are not immediately life-threatening or do not result in death or hospitalisation but may jeopardise the subject or may require intervention to prevent one of the other outcomes listed in the definition above, should also be considered serious.

8.2.2 Suspected unexpected serious adverse reactions (SUSAR)

This section is only applicable to studies with an investigational medicinal product which are subject to the EU clinical trial directive 2001/20/EU.

The investigator assesses whether SAE is expected or not. The final decision is made by the legal sponsor who reports pharmacovigilance to all relevant authorities and institutions.

8.2.3 Trial Specific Events requiring Expedited SAE Notification and Reporting

All serious non-AIDS events as defined in appendix 5 and all AIDS events as defined in appendix 4 must be reported within 24h to the regional CTU.

8.3 Investigator responsibilities

All non-serious AEs, whether expected or not, should be recorded on the AE Form and sent to the NEAT regional CTU within one month of the form being due.

SAEs/SARs should be notified to the NEAT regional CTU as described below.

The severity (i.e. intensity) of all AEs (serious and non-serious) in this trial should be graded using the ANRS scale to grade the severity of adverse events in adults, Version n° 1.0, 4 November 2008 (see appendix 6).
.

8.3.1 Severity and causality of adverse events

(a) Seriousness

The severity of each AE should be graded by the investigator using the ANRS scale to grade the severity of adverse events in adults, Version n° 1.0, 4 November 2008 (see appendix 6). Clinically relevant laboratory adverse events are reported as adverse events on the adverse events pages of the CRF.

(b) Causality

The investigator must assess the causality of all serious events/reactions in relation to the trial therapy using the definitions in Table 4*.* There are 5 categories: unrelated, unlikely, possible, probable and definitely related. If the causality assessment is unrelated or unlikely to be related the event is classified as a SAE. If the causality is assessed as possible, probable or definitely related then the event is classified as a SAR.
The investigator should also assess the causality of the SAE to the research.

Table 4: Definitions of causality

Relationship	Description	Event Type
**Unrelated**	The patient/subject **did not receive** the study medication or placebo.<br>**OR**<br>The temporal sequence of the AE onset **relative** to **administration** of the medication is **not reasonable**.<br>**OR**<br>There is another obvious cause of the AE	SAE
**Unlikely**	There is little evidence to suggest there is a causal relationship (e.g. the event did not occur within a reasonable time after administration of the trial medication). There is another reasonable explanation for the event (e.g. the patient's clinical condition, other concomitant treatment).	SAE
**Possible**	There is some evidence to suggest a causal relationship (e.g. because the event occurs within a reasonable time after administration of the trial medication). However, the influence of other factors may have contributed to the event (e.g. the patient's clinical condition, other concomitant treatments).	SAR
**Probable**	There is **evidence of exposure** to the study medication or placebo.<br>The temporal sequence of the AE onset **relative** to **administration** of the medication is **reasonable.**<br>The AE is **more likely explained** by the medication than by another cause.	SAR
**Definitely**	There is **evidence of exposure** to the study medication or placebo.<br>The temporal sequence of the AE onset **relative** to **administration** of the medication is reasonable.<br>The AE is **more likely explained** by the medication than by another cause.<br>Dechallenge (if performed) is **positive.**<br>Rechallenge (if feasible) is **positive.**<br>The AE shows a **pattern consistent** with **previous knowledge** of the medication or medication class	SAR

(c) Expectedness

If the event is a SAR, the investigator must assess the expectedness of the event. An adverse reaction not listed in the investigator's brochure or in the products characteristics is considered unexpected.

(d) Notification

The NEAT regional CTU should be notified within 24 hours of the investigator becoming aware of an event that requires expedited reporting. Investigators should notify the

NEAT regional CTU of all SAEs occurring from the time of consent form signature until 30 days after the last follow-up visit. SARs and SUSARs must be notified to the NEAT regional CTU indefinitely (i.e. no matter when they occur after consent form signature).

SAE NOTIFICATION PROCEDURE

1. The SAE form must be completed by the investigator (consultant named on the signature list and delegation of responsibilities log who is responsible for the patient's care), with due care being paid to the grading, causality and expectedness of the event as outlined above. In the absence of the responsible investigator the form should be completed and signed by a member of the site trial team. The responsible investigator should subsequently check the SAE form, make changes as appropriate, sign and then re-send to the NEAT regional CTU as soon as possible. The initial report shall be followed by detailed, written reports as appropriate.

2. Send the SAE form to the NEAT regional CTU **within 24 hours** of becoming aware of the event

3. Follow-up: Patients must be followed-up until clinical recovery is complete and laboratory results have returned to normal or baseline, or until the event has stabilised. Follow-up should continue after completion of protocol treatment if necessary. Follow-up information should be noted on a further SAE form by ticking the box marked 'follow-up' and sending it to the NEAT regional CTU as information becomes available. Extra, annotated information and/or copies of test results may be provided separately. The patient must be identified by trial and patient number and date of birth only. The patient's name should not be used on any correspondence.

4. For notification of the event to local ethics committees country-specific regulations apply.

SAE NOTIFICATION

Within 24h of becoming aware of an SAE, please send a completed SAE form to the NEAT regional CTU.:

Fax: 0000000

Randomisation and SAE notification numbers are specific to each regional CTU. Telephone and fax numbers to be inserted by regional CTU

8.3.2 Pregnancy notifications

The pregnancy notification form has to be filled out and needs to be sent to the regional CTU.

8.4 Sponsor's responsibilities

Like the investigator, the legal sponsor (Inserm - ANRS) is responsible of the assessment of the causality of the SAE.
The causality assessment given by the investigator should not be downgraded by the sponsor. If the sponsor disagrees with the investigator' causality assessment, both the opinion of the investigator and the sponsor should be provided with the report.
The expectedness of the SAR shall be determined by the sponsor or its delegate. Adverse reaction should be considered as unexpected if the nature, seriousness, severity or outcome of the reaction is not consistent with the reference information for the investigational medicinal products (IMPs).
The sponsor is also responsible for the prompt notification of SUSARs to Eudravigilance, the Ethics Committees and National Competent Authority of each concerned Member state and to the investigators.
In addition to the expedited reporting, the sponsor or its delegate shall submit once a year throughout the clinical trial a safety report to the National Competent Authority and Ethics Committees of the concerned Member states. The annual safety report may be combined with the annual progress report.

The pharmaceutical companies Gilead Sciences, Merck and Janssen-Cilag may also be notified of all reportable (serious and unexpected and drug related/unknown relationship) events. The legal sponsor may also provide any companies with a copy of the annual safety report (line-listing of SARs and SUSARs).

9. STATISTICAL CONSIDERATIONS

9.1 Sample size

The primary analysis will assess whether the upper bound of the two-sided 95% confidence interval of absolute difference between the proportions of participants with clinical or virological failure between the two arms (DRV/r+RAL arm minus DRV/r + TDF/FTC arm) is less than the predefined non-inferiority margin of 9%, as clarified in protocol amendment 5.0.

Since the trial has completed enrolment at the time of the amendment 5.0, the initial sample size calculation is not modified. For the sample size calculation a non-inferiority margin based on the two-sided 95% confidence interval of hazard ratio (HR), for DRV/r+RAL relative to TDF/FTC + DRV/r, had been chosen as 1.53 so that if non-inferiority is established, the absolute failure rate for DRV/r+RAL at W96 is no more than 9% higher than the 20% rate assumed for DRV/r + TDF/FTC. The sample size calculation was thus based on testing the null hypothesis that HR ≥ 1.53 against the alternative hypothesis that HR < 1.53. The required number of events (failures) was detemined to achieve power = 0.8 if in fact HR = 1, and was calculated using standard methods for comparative trials based on the logrank test [38,39]. Sample size was estimated as 800 patients with an average follow-up of 120 weeks. The assumptions made in determining the sample size to address the primary

objective of the trial for the composite primary endpoint of virological or clinical failure are outlined below:

1. The cumulative probablity of failure in the DRV/r + TDF/FTC arm at week 96 was assumed to be 20% composed of 10% of early virological failure (2% of insufficient virological response at week 18 and 8% of HIV RNA > 50 copies/ml at week 32), 5% of rebound to > 50 copies after week 32 and 5% of clinical failure. This is based on data in the DRV/r + TDF/FTC arm of the ARTEMIS study [9].

2. The total duration of the study was assumed to be 144 weeks. Participants were to be enrolled over a 48-week period and followed for a minimum of 96 weeks; average follow-up was assumed to be 120 weeks and range from 96 to 144 weeks.

3. The cumulative probability of being lost to follow-up (primary endpoint status unkown at the end of the trial) at week 144 was assumed to be 0.10

4. Non-inferiority margin: the absolute difference in failure rate between DRV/r+RAL and DRV/r + TDF/FTC at W96 is no more than 9% representing an increase from 20% to 29%. This is equivalent to a hazard ratio (HR) for DRV/r+RAL relative to DRV/r + TDF/FTC = 1.53.

5. Two-sided $\alpha = 0.05$ for the null hypothesis that HR ≥ 1.53.

6. Power 80% at the alternative hypothesis that HR =1.0

Under the above assumptions, sample size was calculated as 800 patients (400 per arm) with 88 primary events expected in the control arm of DRV/r + TDF/FTC. Under the same assumptions and sample size, the upper bound of the two-sided 95 % confidence interval for the absolute difference in the cumulative failure probability by week 96 would be less than 9% with 85% power.

9.2 Analysis of the primary endpoint

The primary outcome is time to clinical or virological failure as defined in section 3.2.
The primary outcome is analysed using the Kaplan Meier method to calculate the absolute difference in proportions and 95% confidence interval at 96 weeks.
If the upper limit of this confidence interval is less than 9% then this is taken as evidence that darunavir/r + raltegravir is non-inferior to darunavir/r + tenofovir/emtricitabine according to the non-inferiority margin specified in the design. If this upper confidence bound is less than 0% then this is considered as evidence of the superiority of darunavir/r + raltegravir relative to darunavir/r + tenofovir/emtricitabine. The results are summarised with the number and percentage of patients with at least one component of the primary endpoint and the number and percentage for each component. As a secondary analysis, the results are also summarised by the rate of the composite primary endpoint per 100 person years by treatment group, a treatment hazard ratio (HR: darunavir/r + raltegravir relative to darunavir/r + tenofovir/emtricitabine) estimated by a Cox proportional hazards model and a two-sided 95% confidence interval for the hazard ratio.

The primary analysis is by intention to treat and includes all randomised participants. Each participant's follow-up is calculated from randomisation until the first occurrence of any component of the primary endpoint, to a common closing date of the trial (estimated as 96

weeks after the last randomisation) for patients who do not experience any component of the primary endpoint, the date endpoint status was last known, or date of withdrawal of consent for participants who are lost to follow-up for the primary endpoint. Thus censoring because of loss to follow-up will be treated as being independent of the outcome (independent censoring).

Treatment of intermittently missing HIV-1 RNA assessments.
The key visits in the definition of the virological components of the primary endpoints are weeks 18, 24 and 32. If week 18 or week 24 visit was missed then the assessment as to whether insufficient response at this visit had occurred will be based on whether the regimen was changed at the next attended visit (week 24 or week 32). If all three visits (W18, W24 and W32) were missed and the primary endpoint was not reached at the last attended visit before W18, then the follow-up will be censored at W18 regardless of whether the patient had attended after W32. If only W32 visit was missed and the primary endpoint had not been reached before W32, then HIV-1 RNA measurement is to be performed as soon as possible and no later than W40. In that case virological failure at W32 will be assessed according to the level of HIV-1 RNA at the extra measurement (W40). If HIV-1 RNA level was missing at both W32 and W40 then follow-up will be censored at W32.

Sensitivity analyses for the primary endpoint
A sensitivity analysis will be done with the per protocol population, censoring events after a patient stops the initial randomised trial treatment.
Moreover, the primary analysis will be repeated using a value of 200 copies/ml instead of 50 copies to determine virological failue at W32 and viral rebound after week 32.

9.3 Interim monitoring and analyses

Interim analyses are provided for the Independent Data Monitoring Committee (IDMC), but not made public. Only IDMC members and the trial statistician are aware of interim results. Safety and efficacy data are reviewed at regular intervals (at least annually) by the IDMC (see also section 14.4).
A predefined full interim analysis is to be performed when all patients have reached 48 weeks of follow-up. In order to avoid interim results to impact in any ways on the conduct of this open label trial and on the final results, interim results may be publicly released only when the last patient included has completed the week 96 visit or when the trial has been stopped for other reasons, which ever occurs first.

Recommendations to modify or discontinue the study in all patients or in selected subgroups are to be made only if the results are likely to convince a broad range of clinicians including participants in the trial and the general clinical community. The statistical guidelines for consideration of such a recommendation will be based on the Haybittle-Peto criterion of at least 3 standard deviations in the primary outcome.
If a decision is made to continue, the IDMC advises on the frequency of future reviews of the data on the basis of accrual and event rates. The IDMC makes recommendations to the Trial Management Team and the Trial Steering Committee as to the continuation of the trial.
During IDMC review, particular attention should be paid to the safety and efficacy aspects specified in section 14.4. A charter for the IDMC is established in a separate document.

The Trial Steering Committee (TSC) monitors the total number of endpoint events without distinction between treatment arms. Should a lower than expected number of endpoint events be observed, the TSC is asked to give advice on whether the accumulated data from

the trial, together with results from other relevant trials, justifies continuing recruitment of further patients or continuation of the trial beyond the planned timelines.

9.4 Analyses of other data

Before the database is frozen, a more detailed statistical analysis plan needs to be defined in a separate document which is to be used for the analysis. Any deviations of the statistical plan are to be reported in the final study report.

10. DATA MANAGEMENT

10.1 Data collection

Source data must be available to document the existence of the study patients and should substantiate integrity of study data collected. Source data must include the original documents relating to the study, the medical treatment and medical history of the patient.
The following information should be included in the source medical records:

- Demographic and socio-economic data (date of birth, sex, ethnic group, education, working status)
- Study name and protocol number in which the patient participates
- Date of signing informed consent form
- Details related to the inclusion criteria
- Medical history and physical examination details
- Adverse events and concomitant treatment
- Results of relevant examinations
- Laboratory print-outs
- Visit dates and administration of study medications
- Any other relevant information relating to the trial or to patient care

Data collected for each study patient are recorded in a case report form (CRF) or in questionnaires self-completed by the patients. Questionnaires are used to report data on patients' reported outcomes (adherence, quality of life) and some economic data, like loss of productivity.
The investigator is responsible for ensuring that all sections in the CRF are completed correctly and that entries can be verified against source data. If data have been entered incorrectly in a paper CRF, a single line should be drawn through them and the correct data should be entered together with the initials, date and a short reason for correction (if necessary). Paper CRFs should be completed using a black or blue ball-point.

10.2 Data not to be recorded in the CRF

The following data are not recorded in the CRF:

- Results of assessments performed in a central laboratory
- Results assessed by a central reader

However, it needs to be recorded in the CRF that the procedures for these measurements have been performed (e.g. blood samples taken; samples or images sent to central laboratory/central reader).

10.3 Data quality control

10.3.1 Data monitoring at the regional CTUs

Data stored on the NEAT trial-specific database is checked for missing or unusual values (range checks) and checked for consistency within participants over time. If any such problems are identified, the local site is contacted by writing and asked for confirmation or correction, as appropriate – any data which are changed should be traceable. The amended version should be returned to the regional CTU and the site's copy should also be amended. The regional CTU sends reminders for any overdue and missing data.

10.3.2 On site monitoring

A monitoring plan, describing quality control of collected data in comparison to on-site source data, has been developed as a separate document.

10.3.3 Direct access to source data

Participating investigators agree to allow trial-related monitoring, including audits, ethics committee review and regulatory inspections by providing direct access to source data/documents as required. Source data are monitored with respect of professional confidentiality. Patients' consent for this access to source data is obtained as part of the consent process.

10.4 Further data management

The coordinating CTU is responsible for the data management of the trial. The CRF data are entered in a data management system, which is fully validated and compliant to 21 CFR, part 11. Data entry is done either on site (electronic CRF) or by the regional CTU (paper CRFs). In case of paper CRFs, all completed CRFs are transferred from the study site to the regional CTUs at regular intervals (by post or during site visits).
Data validation checks are performed at regular intervals and may result in data queries. The monitor discusses the queries with the investigator. The resolved queries are to be signed for confirmation by the investigator. The completed queries are returned and the database is updated subsequently.

When all data have been received, all data problems are solved and all data checks and quality control have been performed, a data review meeting has been held, the study database is considered clean and can be locked.
Details regarding data management will be described in specific SOPs.

11. ETHICAL CONSIDERATIONS

Patients consenting to participate in this trial are not able to choose their own initial antiretroviral treatment, the treatment allocation being randomised. Each patient's consent to participate in the trial should be obtained after a full explanation has been given of the treatment options, including the conventional and generally accepted methods of treatment. The right of the patient to refuse to participate in the trial without giving reasons must be respected. After the patient has entered the trial, the clinician must remain free to give alternative treatment to that specified in the protocol, at any stage, if he/she feels it to be in the best interest of the patient. However, the reason for doing so should be recorded and the patient should remain within the trial for the purpose of follow-up and data analysis according to the treatment option to which they have been allocated. Similarly, the patient must remain free to withdraw at any time from the protocol treatment and trial follow-up without giving reasons and without prejudicing his/her further treatment.

11.1 Ethical approval

Before the initiation of a study regulatory approval by the National Competent Authorities and local Ethical Committees or Institutional Review Boards is obtained for conduct of the study.

The trial only starts after written approval from an Independent Ethics Committee (IEC) or Institutional Review Board (IRB) that operates according to ICH GCP guidelines. The written IEC/IRB approval and the names and qualifications of members of the IEC/IRB must be made available to the legal sponsor before the trial can start. The sponsor or its delegate is, together with the investigator, responsible for submission to and communication with the IEC/IRB as well as for obtaining approval of all subsequent major changes, in compliance with local law.

The investigator should conduct the study in accordance with this protocol, the declaration of Helsinki (version 2008) and the ICH GCP guidelines (CPMP/ICH/135/95). The investigator and the sponsor sign the study agreement to confirm this.

11.2 Subject confidentiality

Individual subject medical information, obtained as result of this study is considered confidential and disclosure to third parties is prohibited. Such medical information may be given to the subject's physician or to other appropriate medical personnel responsible for the subject's well being.

Data generated as a result of this study are anonymised and are to be available for inspection on request by the participating physicians, the sponsor's monitor, the IEC/IRB and the regulatory health authorities, including external site audits and inspections.

11.3 Amendments

Amendments are changes made to the research protocol after a favourable opinion by the accredited IEC/IRB has been given. All amendments are to be notified to the IEC/IRB that gave a favourable opinion on the initial protocol.

A 'substantial amendment' is defined as an amendment to the terms of the IEC/IRB application, or to the protocol or any other supporting documentation, that is likely to affect to a significant degree:
- the safety or physical or mental integrity of the subjects of the trial,
- the scientific value of the trial,
- the conduct or management of the trial, or
- the quality or safety of any intervention used in the trial.

All substantial amendments are to be presented to the IEC/IRB for approval and after approval be recorded and filed by the sponsor. All amendments are notified to competent authorities.

Non-substantial amendments are only notified to the accredited IEC/IRB and competent authority, and are recorded and filed by the sponsor. Examples of non-substantial amendments are typing errors and administrative changes like changes in names, telephone numbers and other contact details of involved persons mentioned in the submitted study documentation.

The investigator does not implement any amendment of the protocol without written agreement by the sponsor, except when necessary to eliminate immediate hazard to study subjects.

11.4 Annual progress report (country specific)

The sponsor/investigator or its delegate submits a summary of the progress of the trial to the accredited IEC/IRB and comptent authority once a year. Information is provided on the date of inclusion of the first subject, numbers of subjects included and numbers of subjects that have completed the trial, serious adverse events/ serious adverse reactions, other problems, and amendments.

11.5 End of study report

The sponsor/investigator or its delegate notifies the accredited IEC/IRB and the competent authority of the trial close out within a period of 90 days. The trial is considered closed one year after last patient's last visit, and after formal notification to the centres by the sponsor.

In case the trial is ended prematurely, the investigator/sponsor notifies the accredited IEC/IRB and the competent authority within 15 days, including the reasons for the premature termination.

Within one year after last patient's last visit, the investigator/sponsor submits a summary of the clinical trial report to the Competent Authorities in accordance with regulatory requirements.

11.6 Quality assurance

11.6.1 Definitions

Quality assurance includes all the planned and systematic actions established to ensure the trial is performed and data generated, documented/recorded and reported in compliance with GCP and applicable regulatory requirements.
Quality control includes the operational techniques and activities done within the Quality assurance system to verify that the requirements for quality of the trial-related activities are fulfilled.

11.6.2 Audits

Audits of the study may be arranged by reviewing the data obtained as well as the procedural aspects. This may include on-site audits and source data checks. Direct access to source documents is required for the purpose of these audits.

12. ADMINISTRATIVE MATTERS

12.1 Financing and insurance

The NEAT 001/ANRS 143 trial is financed by NEAT and ANRS, with financial support from Merck and Janssen-Cilag. Legal sponsor of the trial is Inserm - ANRS.

Trial drugs are provided by Gilead Sciences, Merck and Janssen-Cilag.

Clinical Trial Agreement between all parties involved in the study need to be in place prior the start of the study.

The sponsor arranges adequate patient insurance for the period of the study (see Appendix 20).

12.2 Investigator indemnity

If adhered to the protocol by the investigator, the sponsor agrees to, and does hereby, indemnify, defend and hold the investigator harmless from and against all claims, demands, actions and proceedings, which may be brought or asserted against the investigator to recover damages and losses for or attributable to bodily injury, sickness, disease, or death, arising from or alleged to arise from or be reasonable attributable to the above study.

Further details of the indemnity are specified in the agreement between the investigator and the sponsor or its delegate.

12.3 Publication policy

The results of the study (positive or negative) must be made publicly available. The Chair of the Trial Management Team is responsible for publication of the study results. All publications shall include a list of participants, and if there are named authors, these should include the main persons responsible for the trial and its results. If there are no named authors (i.e. group authorship) then a writing committee is identified that would usually include these people. The principles of generally accepted specifications for authorship shall be followed in the appointing of authors and co-authors. All contributors to a specific publication (abstract, original report) have a true opportunity for full evaluation of the final text before submission to a scientific meeting or an editor.

The results from different centres are analysed together and published as soon as possible. Individual clinicians must not publish data concerning their patients that are directly relevant to questions posed by the study until the report of the overall results of the trial has been published.

The trial identifier that has been allocated to this trial should be attached to any publications resulting from this trial.
The members of the TSC and IDMC are to be listed with their affiliations in the Acknowledgements/Appendix of the main publication.

Any publication has to be reviewed by the NEAT 001/ANRS 143 Trial Steering Committee (TSC).

12.4 Study documentation and record keeping

The study documentation includes the following documents:

- Study protocol (and amendments)
- Investigational Medicinal Product Dossier
- Subject Information documents (including Informed Consent)
- CRFs and SAE report forms
- Diaries and/or questionnaires
- Investigator’s File
- Study medication documentation
- Study contract.

In order to start the trial, the investigator is required to have the following documentation available:

- Signed confidentiality agreement
- Signed investigator statement for this protocol
- Signed study contract
- IEC approval letter, stating the sponsor’s name, study number and investigational drug, as well as all documents reviewed and should include a list of members present at the meeting.

- Recent CVs of investigators and sub investigators (signed and dated)
- Signature sheet, documenting delegation of tasks and names and initials of the investigator team.
- If applicable: Import licences
- Regulatory approval
- Laboratory normal ranges and quality certificates and/or accreditations.

During the trial, the regional CTUs collect all completed CRFs.

The investigator archives all study data (subject ID code list, source data, CRF copies and Investigator File) and relevant correspondence. These documents are to be kept on file for at least 15 years after completion of the study or another time period as specified by local laws. The sponsor archives and retains all documents pertaining to the study for the lifetime of the product.

12.5 Sample handling

Blood and urine samples are collected at the study site. The study site is responsible for cytopreservation, separation and storage of all samples collected at the study site as prescribed by the protocol.

The samples which have to be transported to central laboratories or to a central biobank, are stored at the study site until shipment. The study site provides yearly reports of the use and fate of the blood samples to the regional CTU. The handling and shipment of these samples is detailed in a separate document (trial specific SOP).

All laboratories involved shall use the human materials sampled in the study only in accordance with the agreed protocol, the international Good Clinical Practice principles as laid down by the ICH Harmonised Tripartite Guidelines for Good Clinical Practice (ICH GCP), the EU Clinical Trial Directive and relevant other legislation or regulatory requirements as may be required in the Territory.

The regional CTU shall supply study sites with guidance on handling and storage of the materials, and other related relevant information.

All proposals for additional laboratory assessments that are not specified in this protocol must be submitted to the Trial Steering Committee (TSC) and approved prior to their implementation.

13. TRIAL COMMITTEES

13.1 Trial Development Team

The Trial Development Team (TDT) is the team that designs the trial, develops the protocol, sources for the appropriate funding and drug supply, and makes strategic decisions about

the centres and countries that are encouraged to participate in the trial. The TDT is appointed by the NEAT steering committee.

The TDT is lead by the TDT Chair and comprises the following persons:

François Raffi, Nantes, France (Chair)
Anton Pozniak, London, UK (Co-Chair)
Stefano Vella, Rome, Italy (Vice chair)
Clotilde Allavena, Nantes, France (Clinical Coordinator)
Laura Richert, Bordeaux, France (Project Coordinator)
Andrea Antinori, Rome, Italy (Senior Trial Clinician)
José Arribas, Madrid, Spain (Senior Trial Clinician; Coordinator toxicity working group)
Abdel Babiker, London, UK (Trial Statistician)
Geneviève Chêne, Bordeaux, France (Head of Coordinating CTU)
Nikos Dedes, EATG (Community Representative)
Andrzej Horban, Warsaw, Poland (East/Central Europe coordinator)
Deenan Pillay, London, UK (Coordinator virology working group)
Brigitte Autran, Paris, France (Coordinator immunology working group)
Marta Boffito, London, UK (Coordinator pharmacology & adherence working group)
Rita Murri, Rome, (Coordinator Quality of life working group)

TDT members are nominated by the NEAT Steering Committee. The TDT meets in person or by teleconference, with meeting frequency determined by the Chair titrated to the required pace of trial development and the need to address urgent issues that arise. The TDT meetings are coordinated by the designated coordinating CTU for the trial. Much of the work in trial design and protocol development is also done by e-mail communication and review of documents, and this is also facilitated by the coordinating CTU.

As the trial preparation matures, the work of the TDT is gradually be handed to the Trial Steering Committee and the formal responsibility for the trial ends with the first meeting of the Trial Steering Committee (see below).

13.2 Trial Steering Committee

The Trial Steering Committee (TSC) is the group that provides overall supervision for the trial on behalf of the sponsor and NEAT. It substitutes the TDT before the trial starts.

The TSC is lead by a Chairman who is independent from the trial and includes the following people:

Eric Sandström, Stockholm, Sweden (independent member, chair)
Hans-Jürgen Stellbrink, Hamburg, Germany (independent member)
Markus Flepp, Zürich, Switzerland (independent member)
Siegfried Schwarz, EATG (Community Representative, independent member)
Jean-Michel Molina, Paris, France (Sponsor representative, ANRS)
José Gatell, Barcelona, Spain (NEAT Steering Committee Representative)
Christiane Moecklinghoff (Janssen-Cilag representative)
Gillian Pearce (Gilead representative)
Randi Leavitt (Merck representative)
François Raffi, Nantes, France (Trial Management Team Chair)
Anton Pozniak, London, UK (Trial Management Team Co-Chair)

Stefano Vella, Rome, Italy (Trial Management Team Vice Chair)
Abdel Babiker, London, UK (Trial Statistician)
Laura Richert, Bordeaux, France (Project Coordinator)

The TSC is appointed by the NEAT Steering Committee and the legal sponsor.

A charter describes the functioning of the TSC. The frequency and method of meetings are determined by the TSC and may vary with the nature, risks and complexity of the trial, but usually comprise meetings (in person or by teleconference) every 6 months throughout the trial. Representatives form regional CTUs are invited to TSC meetings as non-voting participants.

The TSC Chairman reports to the NEAT Steering Committee every 6 months, and reports to the Trial Sponsor at the required frequency.

13.3 Trial Management Team

The Trial Management Team (TMT) is the operational team that undertakes the day-to-day management of the trial.

The TMT consists of the following members:

François Raffi, Nantes, France (Chair)
Anton Pozniak, London, UK (Co-Chair)
Stefano Vella, Rome, Italy (Vice Chair)
Clotilde Allavena, Nantes, France (Clinical Coordinator)
Laura Richert, Bordeaux, France (Project Coordinator)
Abdel Babiker, London, UK (Trial Statistician)
Geneviève Chêne, Bordeaux, France (Head of Coordinating CTU)
Juliette Saillard, Paris, France (ANRS)
Lucia Palmisano, Rome, Italy (NEAT management representative)
Two representatives per regional CTU

The TMT meets by teleconferences, which are usually held at monthly intervals although may be needed more frequently in the initial phases.

The TMT (represented by the Project Coordinator or the TMT chair) formally reports to the TSC on the progress of the trial periodically (at least every 6 months). This takes place at the regular meetings of the TSC, but any interim urgent issues will be communicated to the Chairman of the TSC as they arise, and the Chairman ensures appropriate communication to the TSC with ad-hoc meetings held if needed.

13.4 Independent Data Monitoring Committee

The Independent Data Monitoring Committee (IDMC) is the group that monitors the main safety and efficacy outcome measures and the overall conduct of the trial, with the aim of protecting the safety and the interests of the trial participants. The IDMC is lead by a Chairman and comprises the following members:

Tim Peto, Oxford, UK (chair)

Frank Goebel, Munich, Germany (clinician)
Veronica Miller, Washington DC, USA (methodologist/epidemiologist)
Peter Sasieni, London, UK (statistician)
Simone Marcotullio, Rome, Italy (community nominated person)

The IDMC is appointed by the TSC and approved by the legal sponsor.

All IDMC members must be free from any direct involvement with the trial. Any competing interests, both real and potential, must be declared.

A charter describes the functioning of the IDMC. The frequency and method of meetings are determined by the IDMC, and may vary with the nature, risks and complexity of the trial, but usually comprise meetings (in person or by teleconference) every 6 months throughout the trial. Additional meetings can be summoned by the chair of the IDMC, the chair of the TMT or by the legal sponsor.
The TMT prepares a report for each IDMC meeting (outcome data by treatment group is only prepared by the trial statistician).

At each meeting, the IDMC should perform an interim review of the trial's progress including updated figures on recruitment, data quality, and main outcomes and safety data.

More specifically, the following aspects should be addressed:

- verification that a sufficient number of patient with a CD4 count < 200/mm^3 is enrolled in the trial (aim: 20-25 %)
- verification that a representative proportion of women is enrolled in the trial
- assessment of the viral load decay in each treatment arm
- assessment of the rate of clinical and virologic primary endpoint components in each treatment arm
- assessment of the rate and reason of trial regimen discontinuation for toxicity or intolerance in each treatment arm
- assessment of efficacy and safety in patients with a low CD4 count at inclusion
- monitoring of serious adverse events
- assessment of cancer incidence
- assessment of incidence of rash

Although failure to achieve virologic response by W32 is a component of the combined primary endpoint, it is recommended that decisions on the continuation of the trial should not be automatically taken solely on this criterion, as information on clinical outcome and tolerability after 2 years of follow-up is a very important information of this strategic trial.
The IDMC should balance not only virologic outcomes (lack of virologic response, virologic failure, resistance at failure and its consequences), but also clinical events, tolerability and toxicity, drug discontinuation and immunologic outcome.

The IDMC can recommend continuation without change, continuation with change, or premature closure of the trial to the TMT and to the TSC.
After each meeting, the IDMC provides a brief formal written report and report its recommendations within 15 days in writing to the Chair of the TMT and to the TSC.

13.5 Endpoint Review Committee

An Endpoint Review Committee is appointed for this trial. It is composed of 7-8 experienced clinicians, who rotate for the different reviews sessions, and one permanent chair.

The ERC reviews the following events:
- AIDS defining events
- serious non-AIDS defining events
- grade 4 adverse clinical events
- grade 2 to 4 rashes
- deaths
- immune reconstitution syndrome events

The members of the ERC are unaware of the treatment assignment of the patient (blinded review). ERC members are appointed by the TSC.

13.6 Protocol Team (the NEAT 001/ANRS 143 Agorà)

The Protocol Team assembles the TMT members, former TDT members, sub-study working groups (see appendix 18) and national coordinating investigators (see appendix 19) to discuss scientific issues during the trial. The Protocol Team may make suggestions to the TSC regarding the scientific conduct of the trial. Final decisions lie with the TSC. The Protocol Team is chaired by François Raffi and co-chaired by Anton Pozniak.

14. REFERENCES

1. Hirsch MS. Initiating therapy: when to start, what to use. J Infect Dis 2008;197 Suppl 3:S252-260.
2. European AIDS Clinical Society. Clinical management and treatment of HIV infected adults in Europe. EACS Guidelines Version 5; November 2009.
3. Panel on Antiretroviral Guidelines for Adults and Adolescents. Guidelines for the use of antiretroviral agents in HIV-1-infected adults and adolescents. Volume 2009: Department of Health and Human Services; December 1, 2009. p 1-161.
4. Lewden C, Chene G, Morlat P, Raffi F, Dupon M, Dellamonica P, Pellegrin JL, Katlama C, Dabis F, Leport C. HIV-infected adults with a CD4 cell count greater than 500 cells/mm3 on long-term combination antiretroviral therapy reach same mortality rates as the general population. J Acquir Immune Defic Syndr 2007;46(1):72-77.
5. Haubrich RH, Riddler SA, DiRienzo AG, Komarow L, Powderly WG, Klingman K, Garren KW, Butcher DL, Rooney JF, Haas DW, Mellors JW, Havlir DV. Metabolic outcomes in a randomized trial of nucleoside, nonnucleoside and protease inhibitor-sparing regimens for initial HIV treatment. AIDS 2009;23(9):1109-1118.
6. Hirsch MS, Gunthard HF, Schapiro JM, Brun-Vezinet F, Clotet B, Hammer SM, Johnson VA, Kuritzkes DR, Mellors JW, Pillay D, Yeni PG, Jacobsen DM, Richman DD. Antiretroviral drug resistance testing in adult HIV-1 infection: 2008 recommendations of an International AIDS Society-USA panel. Clin Infect Dis 2008;47(2):266-285.
7. Hammer SM, Eron JJ, Jr., Reiss P, Schooley RT, Thompson MA, Walmsley S, Cahn P, Fischl MA, Gatell JM, Hirsch MS, Jacobsen DM, Montaner JS, Richman DD, Yeni PG, Volberding PA. Antiretroviral treatment of adult HIV infection: 2008 recommendations of the International AIDS Society-USA panel. JAMA 2008;300(5):555-570.
8. Ortiz R, Dejesus E, Khanlou H, Voronin E, van Lunzen J, Andrade-Villanueva J, Fourie J, De Meyer S, De Pauw M, Lefebvre E, Vangeneugden T, Spinosa-Guzman S. Efficacy and safety of once-daily darunavir/ritonavir versus lopinavir/ritonavir in treatment-naive HIV-1-infected patients at week 48. AIDS 2008;22(12):1389-1397.
9. Mills AM, Nelson M, Jayaweera D, Ruxrungtham K, Cassetti I, Girard PM, Workman C, Dierynck I, Sekar V, Abeele CV, Lavreys L. Once-daily darunavir/ritonavir vs. lopinavir/ritonavir in treatment-naive, HIV-1-infected patients: 96-week analysis. AIDS 2009.
10. Nelson M, Yeni P, Sension M, Ive P, Chen L, Hill A, Demasi R, S S-G. Factors Affecting Virologic Response to Darunavir/Ritonavir and Lopinavir/Ritonavir in Treatment-naïve HIV Patients: ARTEMIS at 96 Weeks. 16th Conference on Retroviruses and Opportunistic Infections. Montreal, Canada; 2009.
11. Boffito M, Collot-Teixeira S, De Lorenzo F, Waters L, Fletcher C, Back D, Mandalia S, Pozniak A, McGregor J, B G. Plasma exposure of 100 mg once (OD) and twice daily (bid) decreases HDL and CD36 expression but only bid dosing increases triglycerides: potential impact of ritonavir on cardiovascular disease. 15th Conference on Retroviruses and Opportunistic Infections. Boston, USA; 2008.
12. Center for Drug Evaluation and Research, U.S. Food and Drug Administration. Information for Healthcare Professionals Darunavir Ethalolate (marketed as Prezista) Volume 2008; 2008.
13. Hazuda DJ, Felock P, Witmer M, Wolfe A, Stillmock K, Grobler JA, Espeseth A, Gabryelski L, Schleif W, Blau C, Miller MD. Inhibitors of strand transfer that prevent integration and inhibit HIV-1 replication in cells. Science 2000;287(5453):646-650.
14. Murray JM, Emery S, Kelleher AD, Law M, Chen J, Hazuda DJ, Nguyen BY, Teppler H, Cooper DA. Antiretroviral therapy with the integrase inhibitor raltegravir alters decay

kinetics of HIV, significantly reducing the second phase. AIDS 2007;21(17):2315-2321.
15. Markowitz M, Morales-Ramirez JO, Nguyen BY, Kovacs CM, Steigbigel RT, Cooper DA, Liporace R, Schwartz R, Isaacs R, Gilde LR, Wenning L, Zhao J, Teppler H. Antiretroviral activity, pharmacokinetics, and tolerability of MK-0518, a novel inhibitor of HIV-1 integrase, dosed as monotherapy for 10 days in treatment-naive HIV-1-infected individuals. J Acquir Immune Defic Syndr 2006;43(5):509-515.
16. Markowitz M, Nguyen BY, Gotuzzo E, Mendo F, Ratanasuwan W, Kovacs C, Wan H, Campbell H, Isaacs R, H T. Sustained antiretroviral efficacy of raltegravir as part of combination ART in treatment-naive HIV-1 infected patients: 96-week data. 17th International AIDS Conference. Mexico City, Mexico; 2008.
17. Markowitz M, Nguyen BY, Gotuzzo E, Mendo F, Ratanasuwan W, Kovacs C, Prada G, Morales-Ramirez JO, Crumpacker CS, Isaacs RD, Gilde LR, Wan H, Miller MD, Wenning LA, Teppler H. Rapid and durable antiretroviral effect of the HIV-1 Integrase inhibitor raltegravir as part of combination therapy in treatment-naive patients with HIV-1 infection: results of a 48-week controlled study. J Acquir Immune Defic Syndr 2007;46(2):125-133.
18. Cooper D, Gatell J, Rockstroh J, Katlama C, Yeni P, Lazzarin A, Xu X, Isaacs R, Teppler H, Nguyen BY, Group tB-S. 48-Week results from BENCHMRK-1, a phase III study of raltegravir in patients failing ART with triple-class resistant HIV-1. 15th Conference on Retroviruses and Opportunistic Infections. Boston, USA; 2008.
19. Grinsztejn B, Nguyen BY, Katlama C, Gatell JM, Lazzarin A, Vittecoq D, Gonzalez CJ, Chen J, Harvey CM, Isaacs RD. Safety and efficacy of the HIV-1 integrase inhibitor raltegravir (MK-0518) in treatment-experienced patients with multidrug-resistant virus: a phase II randomised controlled trial. Lancet 2007;369(9569):1261-1269.
20. Steigbigel R, Kumar P, Eron J, Schechter M, Markowitz M, Loutfy M, Zhao J, Isaacs R, Nguyen BY, Teppler H, Group tB-S. 48-Week results from BENCHMRK-2, a phase III study of raltegravir in patients failing ART with triple-class resistant HIV. 15th Conference on Retroviruses and Opportunistic Infections. Boston, USA; 2008.
21. Steigbigel RT, Cooper DA, Kumar PN, Eron JE, Schechter M, Markowitz M, Loutfy MR, Lennox JL, Gatell JM, Rockstroh JK, Katlama C, Yeni P, Lazzarin A, Clotet B, Zhao J, Chen J, Ryan DM, Rhodes RR, Killar JA, Gilde LR, Strohmaier KM, Meibohm AR, Miller MD, Hazuda DJ, Nessly ML, DiNubile MJ, Isaacs RD, Nguyen BY, Teppler H. Raltegravir with optimized background therapy for resistant HIV-1 infection. N Engl J Med 2008;359(4):339-354.
22. Lennox JL, DeJesus E, Lazzarin A, Pollard RB, Madruga JVR, Berger DS, Zhao J, Xu X, Williams-Diaz A, Rodgers AJ, Barnard RJO, Miller MD, DiNubile MJ, Nguyen B-Y, Leavitt R, Sklar P. Safety and efficacy of raltegravir-based versus efavirenz-based combination therapy in treatment-naive patients with HIV-1 infection: a multicentre, double-blind randomised controlled trial. The Lancet 2009;374(9692):796-806.
23. Lennox J, Dejesus E, Lazzarin A, Pollard R, Madruga J, J Z. Subgroup Analyses from STARTMRK, a Phase III Study of Raltegravir-based vs Efavirenz-based Combination Therapy in Treatment-naïve HIV-infected Patients. 16th Conference on Retroviruses and Opportunistic Infections. Montreal, Canada; 2009.
24. Lennox J, DeJesus E, Lazzarin A, Berger D, Pollard R, Madruga J, Zhao J, Gilbert C, Rodgers A, Teppler H, Nguyen BY, Leavitt R, Sklar P. Raltegravir demonstrates durable efficacy through 96 weeks (wk): results from STARTMRK, a phase III study of raltegravir (RAL)-based vs efavirenz (EFV)-based therapy in treatment-naive HIV+ patients (pts). 49th Interscience Conference on Antimicrobial Agents and Chemotherapy. San Francisco, California; 2009.

25. Cooper D, Steigbigel R, Lennox J, Grinsztejn B, Markowitz M, Sklar P, Leavitt R, BY N, Xu X, H T. Review of Cancer Incidence in Raltegravir Clinical Trials. 16th Conference on Retroviruses and Opportunistic Infections. Montreal, Canada; 2009.
26. Allavena C, Ferre V, Brunet-Francois C, Delfraissy JF, Lafeuillade A, Valantin MA, Bentata M, Michelet C, Poizot-Martin I, Dailly E, Launay O, Raffi F. Efficacy and tolerability of a nucleoside reverse transcriptase inhibitor-sparing combination of lopinavir/ritonavir and efavirenz in HIV-1-infected patients. J Acquir Immune Defic Syndr 2005;39(3):300-306.
27. Hirschel B, Calmy A. Initial treatment for HIV infection--an embarrassment of riches. N Engl J Med 2008;358(20):2170-2172.
28. Anderson MS, Sekar V, Tomaka F, Mabalot J, Mack R, Lionti L, Zajic S, Wenning L, Vanden Abeele C, Zinny M, Lunde NM, Jin B, Wagner JA, Iwamoto M. Pharmacokinetic (PK) Evaluation of Darunavir/Ritonavir (DRV/r) and Raltegravir (RAL) in Healthy Subjects. 48th Annual ICAAC/IDSA 46th Annual Meeting. Washington, DC; 2008.
29. Long K, Soulié C, Schneider L, Ghosn J, Piketty C, Boué F, Reynes J., Raffi F., Calvez V., Katlama C., G. P. Therapeutic Drug Monitoring of Raltegravir (MK-0518) in Experienced HIV-infected Patients. 11th European AIDS conference. Madrid, Spain; 2007.
30. Podsadecki T, Tian M, Fredrick L, Lawal A, Bernstein B. Lopinavir/Ritonavir (LPV/r) combined with raltegravir (RAL) provides more rapid viral decline than LPV/r combined with tenofovir disoproxil fumarate/emtricitabine (TDF/FTC) in treatment-naïve HIV-1 infected subjects. 15th Annual Conference of the British HIV Association (BHIVA). Liverpool, UK; 2009.
31. Gallant JE, Staszewski S, Pozniak AL, DeJesus E, Suleiman JM, Miller MD, Coakley DF, Lu B, Toole JJ, Cheng AK. Efficacy and safety of tenofovir DF vs stavudine in combination therapy in antiretroviral-naive patients: a 3-year randomized trial. JAMA 2004;292(2):191-201.
32. Mills A, Nelson M, Jayaweera D, Ruxrungtham K, Cassetti I, Girard P, Workman C, Dierynck I, Sekar V, Vanden Abeele C, L L. Efficacy and safety of darunavir/ritonavir 800/100 mg once-daily versus lopinavir/ritonavir in treatment-naive, HIV-1-infected patients at 96 weeks: ARTEMIS (TMC114-C211). 48th Annual ICAAC/IDSA 46th Annual Meeting Washington, DC; 2008.
33. EMEA. Isentress, Summary of Product Characteristics - 09/09/2009 Isentress-H-C-860-II-10. Volume 2009; 2008.
34. Haubrich RH, Riddler S, DiRienzo G, Komarow L, Powderly W, Karren G, George T, Rooney JF, Mellors JW, Havlir D, Team tACTGS. Metabolic outcomes of ACTG 5142: A prospective, randomized, phase III trial of NRTI-, PI-, and NNRTI-sparing regimens for initial treatment of HIV-1 infection. 14th Conference on Retroviruses and Opportunistic Infections. Los Angeles, USA; 2007.
35. Zolopa A, Andersen J, Komarow L, Sanchez A, Suckow C, Sanne I, Hogg E, Powderly W, Team AAS. Immediate vs Deferred ART in the Setting of Acute AIDS-related Opportunistic Infection: Final Results of a Randomized Strategy Trial, ACTG A5164. 15th Conference on Retroviruses and Opportunistic Infections. Boston, USA; 2008.
36. Rockstroh JK, Bhagani S, Benhamou Y, Bruno R, Mauss S, Peters L, Puoti M, Soriano V, Tural C. European AIDS Clinical Society (EACS) guidelines for the clinical management and treatment of chronic hepatitis B and C coinfection in HIV-infected adults. HIV Med 2008;9(2):82-88.
37. French MA, Price P, Stone SF. Immune restoration disease after antiretroviral therapy. AIDS 2004;18(12):1615-1627.

38. Royston P. A menu-driven facility for complex sample size calculation in randomized controlled trials with a survival or a binary outcome. Stata Journal 2002;2(2):151-163.
39. Barthel FMS, Babiker A, Royston P, Parmar MKB. Paper Celebrating the 25th Anniversary of Statistics in Medicine. Evaluation of sample size and power for multi-arm survival trials allowing for non-uniform accrual, non-proportional hazards, loss to follow-up and cross-over. Statistics in Medicine 2006;25(15):2521-2542.

15. APPENDICES

Appendix 1: Sample patient information sheet
Appendix 2: Sample consent form
Appendix 3: Sample pharmacogenomics consent
Appendix 4: CDC classification system for HIV infection (revision 1993)
Appendix 5: Serious non-AIDS events
Appendix 6: Table for grading adverse experiences
Appendix 7: Estimation of creatinine clearance (Cockcroft and Gault equation)
Appendix 8: Biobank procedures and assessments
Appendix 9: Pharmacogenetics and population pharmacokinetics
Appendix 10: Viral sub-type analysis
Appendix 11: Renal function study
Appendix 12: Quality of life assessments
Appendix 13: Adherence study
Appendix 14: Pharmacoeconomics study
Appendix 15: Viral and immunologic dynamics and inflammation sub-study
Appendix 16: Bone sub-study
Appendix 17: Neurocognitive function sub-study
Appendix 18: Working groups
Appendix 19: Trial management
Appendix 20: Insurance policy

APPENDIX 1: SAMPLE PATIENT INFORMATION SHEET

(To be translated into local languages and presented on local headed paper; content of the patient information sheet is country-dependent). The patient information sheet must be dated and given a version number.

PATIENT INFORMATION SHEET

Centre Number (if applicable): xxx

V3.0, November 19, 2010
[Replace by local date and version]

Title of the trial: NEAT 001/ANRS 143 trial
Clinicaltrials.gov identifier: <insert>

1. Study title

An open-label randomised comparative trial comparing first-line regimens in HIV-infected antiretroviral naïve subjects: darunavir/r + tenofovir/emtricitabine vs. darunavir/r + raltegravir

2. Invitation paragraph

You are being invited to take part in a research study. Before you decide it is important for you to understand why the research is being done and what it will involve. Please take time to read the following information carefully and discuss it with others if you wish. This consent form may contain words that you do not understand. Please ask a Study Doctor or a member of the study staff to explain any words that you do not know, or any information that is unclear or confusing. Ask the research doctor if there is anything that is not clear or if you would like more information. Take time to decide whether or not you wish to take part. Once you understand the study, you will be asked if you wish to participate; if so, you will be asked to sign a consent form. A copy of this patient information and consent form will be provided to you for future reference.
Thank you for reading this.

3. What is the purpose of the study?

Since 1996, antiretroviral therapies have led to a decrease in HIV-related mortality. Antiretroviral treatment in HIV-infected patients requires a long-term administration. Generally, three drugs are combined for treatment. Although the efficacy of most antiretroviral regimens today is excellent, long-term side effects of these treatments remain a major concern.
To select initial treatment, it is important to identify regimens which combine both optimal effectiveness, long-term safety, ease of dosing and low pill burden. Novel combinations of antiretroviral drugs may have the advantage of an excellent efficacy combined with little long-term side effects and with convenient treatment modalities.

The aim of this study is to compare over at least 2 years the efficacy and safety of two different combinations of antiretroviral drugs in adults infected with HIV-1 who have never taken drugs to treat their HIV infection before starting this study. The two treatment strategies that are being compared are:

Group 1: darunavir/ritonavir + tenofovir/emtricitabine

This is a treatment strategy combining three different drugs: two nucleoside reverse transcriptase inhibitors (NRTIs) and one protease inhibitor (PI) boosted with a small dose of

ritonavir. The efficacy and tolerance of this combination have been studied before. Darunavir and tenofovir/emtricitabine are recommended for use in HIV-infected patients.

Group 2: darunavir/r + raltegravir

This is a new treatment strategy combining two drugs: a protease inhibitor (darunavir) boosted with a small dose of ritonavir, and a new class of drugs (integrase inhibitor raltegravir). The combination of these two drugs is only recently being tested, and only very few data are available in HIV infected treatment experienced patients. Both individual drugs have already been studied for HIV treatment in combination with other drugs, and are recommended for use in HIV-infected patients.

Because we do not know which way of treating patients is best, we need to make comparisons. Once you have agreed to enter the study, you will be allocated one of the two treatments. Chance will decide which of the treatment you will receive (like throwing a dice). This principle is called randomisation. The chances to receive each treatment are equal. Allocating treatment this way helps to insure that the groups of people getting each treatment should be similar. If there are any differences between how the groups do, it must be due to the treatment. You will know which drugs you are taking because no placebos (inactive dummy pills) are used in the study.

4. Why have I been chosen?

You are infected with HIV, the virus that causes AIDS, and your research doctor recommends that you start an antiretroviral treatment.
To participate to the study, you need to:

- Be 18 years or older, and be infected with HIV-1
- Have signed the written informed consent
- Have never received previous treatment with any antiretroviral drugs
- Have a HIV-1 viral load over1000 copies/milliliter
- Have an indication to start an antiretroviral treatment

Some medical history or abnormal values on blood test do not allow you to participate. Your research doctor will assess that you fulfill all requirements for participation. Women who are pregnant or breastfeeding or expect to conceive within the duration of the study cannot participate. All women participating in the study need to use an effective contraception method. Even if you are receving oral contraceptives, you should, during the whole trial period, use an alternative contraceptive methods, combining both mechanical method and a second method other than an oral contraceptive, to prevent pregnancy.
800 patients from 15 European countries will participate in this study. Your participation in the trial will last between 2 and 3 years. Your last study visit will take place 2 years after the last of the 800 patients has been included in the study. Since all participants do not start the study exactly on the same date, this study end date will only be known after the study has started. Your research doctor will inform you as soon as the study end date is known.

5. Do I have to take part?

It is up to you to decide whether or not to take part. If you decide to take part you will be given this information sheet to keep and be asked to sign a consent form. If you decide to take part you are still free to withdraw at any time and without giving a reason. If you decide to not take part of to withdraw from the study, this will not affect the standard of care you receive.
While you are taking part in this study, you may be asked to take part in some additional related research studies (sub-studies). You may refuse to take part in these sub-studies and still be in this main study.

6. What will happen to me if I take part?

You will be asked to come to the clinic for study visits at regular intervals (at least 13 visits over two years). The first visit (screening visit) is done to check whether you fulfill all criteria to participate in the study. At this visit, your doctor will ask questions about your health, your HIV infection and the medicine you are actually taking and will give you a medical examination. Blood will be drawn and used to measure the amount of HIV in your blood and make a test showing what drugs your HIV virus might not respond to (“resistance testing”). A panel of standard blood and urine tests will be done (immunologic tests, liver and kidney tests). If you are a woman who can get pregnant, you will have a pregnancy test done.

At the second visit these results will be shared with you. Your doctor will tell you if you can participate in the study and if so, which treatment you are allocated to. No matter which group you are assigned to, you will return to the clinic at approximately 2 weeks, 1, 2, 3, 4.5, 6, 8 and 12 months after you started the treatment until the end of the first year and every 4 months after that until the end of the study. All these visits aim at assessing the efficacy of the treatment and your safety. At the visits, your research doctor will do a physical examination and ask you about your general health, what medications you are taking, and whether you have experience any side effects from your treatment. Approximately 30 to 70 ml of blood (3 to 7 soupspoons) will be drawn at each visit.

The blood will be used to measure the amount of HIV in your blood, your CD4 cell count, to check the biological safety of the treatment, along with testing the amount of sugar and the type of lipids (fats) in your blood. The blood tests will require that you have not had anything to eat and only water to drink for at least 10 hours before the visit. You will also be asked for a urine sample, which will be checked for parameters that could indicate that you have a kidney problem. If you are a woman who can get pregnant, a urinary pregnancy test will be done. These results will be shared with you at the next study visit or when they become available.

Some of the blood and urine will be stored at each visit and may be used for future studies of the HIV virus, the immune system and complications of treatments. These samples may be shipped from your country to another for the purpose of centralised storage and analysis. If other studies than those mentioned are planned with the stored blood, you will be asked again for your consent.

At the second visit, and then once a year you will also have an electrocardiogram, a routine test that allows the doctor to look at the rhythm of your heart.

At some visits you will be asked to complete a questionnaire about how you feel physically and emotionally (“quality of life” survey) and about the amount of trial drugs you are taking.

Please be aware that some visits, especially yearly visits, may take more time than a standard visit outside a trial. Your research doctor will ask you to reserve approximately half a day for these “long” visits.

**Week**	**-6 to -1**	**0**	**2**	**4**	**8**	**12**	**18**	**24**	**32**	**48**	**64**	**80**	**96**
Informed consent	x												
Consultation	x	x	x	x	x	x		x	x	x	x	x	x
Do not eat in the morning of the visit (fasting blood sample)	x	x	x	x	x	x	x	x	x	x	x	x	x
Do not take medication in the morning of the visit				x				x					
Pregnancy test (for women)	x	x		x	x	x	x	x	x	x	x	x	x
Sample for biobank		x	x	x	x	x	x	x	x	x	x	x	x
Questionnaires		x		x		x		x		x			x

It is very important to let your doctor know right away if you are sick or injured or in the hospital. This is important for your safety, and also for the study to learn more about the clinical course of patients who are treated for their HIV infection.
Your doctor may decide to see you more often than required for the study based on your needs.

7. What do I have to do?

If you participate in this study, you agree to take the study drugs according to the instructions provided by your research doctor and to come to the clinic for the scheduled study visits. For your own safety, you have to inform your research doctor about your health status and about all medicines, including prescription, over-the-counter (non-prescription), and herbal or alternative medicines you are taking. This is because there may be serious side effects when other medicines are taken with antiretroviral drugs.

8. What is the drug or procedure that is being tested?

You will be assigned to one of the 2 treatment groups. The treatments are as follows:

Group 1:

If you are allocated to this group, you will take the following drugs:

- Prezista®, (darunavir 400mg), 2 tablets once daily, orally and
- Norvir® (ritonavir 100mg), 1 tablet once daily, orally and
- Truvada® (tenofovir/emtricitabine 245/200mg, fixed dose combination), 1 tablet once daily, orally.

Group 2:

If you are allocated to this group, you will take the following drugs:

- Prezista®, (darunavir 400mg), 2 tablets once daily, orally and
- Norvir® (ritonavir 100mg), 1 tablet once daily, orally and
- Isentress® (raltegravir 400mg), 1 tablet twice daily, orally.

9. What are the alternatives for treatment?

If you decide not to take part in the trial, your research doctor can prescribe a therapy for HIV-1 treatment according to the standard of care in your country. He will discuss with you the available treatment options.

10. What are the side-effects of any treatment received when taking part?

The most common side effects of **Truvada®** are diarrhea, nausea, fatigue, headache, dizziness, depression, insomnia, abnormal dreams, and rash. In some cases, renal impairment, including cases of acute renal failure, has been reported with the use of Truvada®. Therefore, your renal function will be monitored during the study.

Main side effects of **Prezista® associated to Norvir®** are diarrhea, nausea, headache, and inflammation of the nose and throat. Some patients on this combination may experience skin rashes; most of which are mild to moderate. Some people may experience large increases in their lipid levels (triglycerides and cholesterol) while being treated with protease inhibitors. However, the side effects of Prezista® associated with Norvir® seem mild to moderate.

The most common side effects of **Isentress®** are nausea, headache and fever. Abnormalities of liver, pancreas or muscle enzymes have also been observed.

For any drug, there may be unknown side effects. It is very important to let your doctor know right away if you are sick or if you have problems that occurred after you started the study treatment. This is important for your safety, and also for the study to learn more about side effects of the drugs.

11. What are the possible disadvantages and risks of taking part?

The study treatment should allow for a control of the HIV virus in your blood without major side effects. However, treatment failure might occur, either due to a lack in virus control, disease progression or side effects. Your research doctor will carefully assess your health status at each visit in order to detect any problem as soon as possible. If necessary, the research doctor may decide to discontinue the treatment allocated by randomisation. In that case, you will be prescribed another antiretroviral combination treatment based on assessment of cause of discontinuation and on the best available other treatment options. Even if you change treatment you will be asked to come to the scheduled visits until the end of the study.
If the treatment is given to a pregnant woman, it is possible that it will harm the unborn child. Pregnant women must therefore not take part in this study, neither should women who plan to become pregnant during the study. Any woman who finds that she has become pregnant while taking part in the study should immediately tell her research doctor. In case you become pregnant, all study drugs that are not recommended during pregnancy will be discontinued, and your research doctor will prescribe a treatment that is authorized during pregnancy. You will be asked to continue your follow-up visits. When the pregnancy is over, the treatment that was initially allocated in the study can be restarted.

Each way of treating HIV disease in this study may be associated with possible benefits and risks. It is not known in the long run which of these treatments will be less risky.

12. What are the possible benefits of taking part?

We hope that all the treatments offered during the study will help you; we hope that the treatments involved will be at least as good as current standard or even better, overall. However, this cannot be guaranteed. It is also possible that you may receive no benefit from being in this study.
By participating in this study, you will play a part in HIV research and help to increase knowledge about HIV treatment. What we can learn from this study may help to improve the treatment of other people who are infected with HIV.

13. Sub-studies : Additional research

Sub-studies treat additional research questions that are in relation with the main trial that has been described above. Your research doctor may propose to you to participate in one or several sub-studies of the trial. You can refuse to participate in a sub-study and still participate in the main trial. There is absolutely no obligation to participate in sub-studies. If you accept to participate in a sub-study, please indicate your decision on the consent form.

a) Viral and immunologic dynamics and inflammation sub-study (“Dynamics sub-study”)
The objective of this sub-study is to study the relationships between the amount of HIV virus in your blood, the immune system (your defenses) and signs of inflammation in your laboratory tests. Markers allowing for the analysis of these relationships can be measured in blood samples. If you participate in this sub-study, the Trial Physician will ask you to come to 3 additional visits: 2-3 days, 1 week, and 3 weeks after treatment start. At these visits and at 7 of the visits scheduled for the main trial, additional blood will be drawn (equivalent to 8 soupspoons of blood per visit). 80 patients will participate in this sub-study.

b) Bone sub-study
The objective of the bone sub-study is to evaluate the effects of the trial drugs on your bones. If you participate in the sub-study, additional assessment will be scheduled at week 0, week 48 and week 96. The density of body tissues, in particular bone and fat, will be measured by an X-ray imaging technique called DXA. This technique does not cause pain and irradiation is weak. In addition, your Trial Physician will ask you about your risk factors for bone fractures,

and additional blood will be drawn at these three visits (1-2 soupspoons of blood) and urine stored. The assessments will allow for estimating your fracture risk. 142 patients will participate in this sub-study.

c) Neurocognitive function sub-study
The objective of this sub-study is to investigate the efficacy of each of the two treatment strategies on cognitive, professional and functional activity measures. If you participate in this sub-study, a trained study nurse or a psychologist will perform neuropsychological tests performed at week 00 and week 96, assessing memory, attention, language and reaction time. The testing procedure takes about 50 minutes at these two visits. 400 patients will participate in this sub-study.

In addition to these three sub-studies, a pharmacogenetic study is proposed to all patients participating in the main trial. Information regarding the pharmacogenetic study will be given to you on a separate document. You will be asked to sign a separate consent form if you agree to participate in this pharmacogenetic study.

14. What if new information becomes available?
Sometimes during the course of a research project, new information becomes available about the treatments that are being studied. If this happens, your research doctor will tell you about it and discuss with you whether you want to continue in the study. If you decide to withdraw, your research doctor will make arrangements for your care to continue. If you decide to continue in the study, you will be asked to sign an updated consent form.

Also, on receiving new information your research doctor might consider it to be in your best interests to withdraw you from the study treatment. He/she will explain the reasons and arrange for your care to continue.

15. What happens when the research study stops?
At the end of the study, your doctor will propose the most appropriate HIV treatment to you. He/she will discuss the different available treatment options with you.

16. What if something goes wrong?
Your research doctor will assess your personal health status regularly in order to detect and address any health problem as soon as possible.

An Independent Committee will assess the overall safety of the trial (i.e. the safety of all participants, not your personal health status) at regular intervals. In case that an important overall safety problem is detected, one trial group or the whole trial may be stopped to ensure your safety.

17. What if I have a complaint?
Your hospital has a duty to care for you within the hospital, whether or not you are taking part in a study.

18. Will my taking part in this study be kept confidential?
All information which is collected about you during the course of the research will be kept strictly confidential. In addition to the clinic/hospital staff, authorized medical and scientific staff responsible for the organisation of the study and staff from Health Authorities may have access to your original patient file to ensure the adequate conduct of the study. Any information about you which leaves the hospital/clinic will have your name and address removed so that you cannot be recognised from it. Your data will be identified only by a code. Only anonymised data relevant for the study will be recorded in a database. This may also include data on treatment costs and resource utilisation, relevant for pharmacoeconomic analyses.

19. What will happen to the results of the research study?

The results of the study (positive or negative) will be made publicly available as soon as possible after the end of the study. The results may be reported at conferences and in medical journals. Published data will always be anonymised, and you will never be identifiable by name or any other way in any publication of this study. You will be informed about the overall results of the study by your research doctor about 1-2 years after the end of the trial.

20. Who is organising and funding the research?

This study is organized by NEAT, a European AIDS treatment network that is funded by the European Union. The legal sponsor responsible for this study is Inserm - ANRS, (French national institute for Health and medical research - French National Agency for AIDS and Hepatitis Research). Inserm - ANRS has signed an insurance policy for liability claims.
The study is publicly funded by the European Union, national governments, and ANRS.
The study drugs and additional financial support are provided by Gilead Sciences, Merck and Janssen-Cilag.
The research team taking care of you at the clinic does not receive any financial incentive to enrol patients in the study.

21. Who has reviewed the study?

This study has been reviewed by an Ethics Committee. If you are interested in knowing more about the review process and the responsible Ethics Committee, your research doctor can give you more information.

22. Contact for Further Information

You should give the patient a contact point(s) for further information. This can be a doctor and/or nurse involved in the study locally.

23. What is the next step?

Take your time to consider your participation in this study and to discuss all aspects with the research doctor, nurses or other persons. Please inform the research team about your final decision.
If you decide to participate, please date and sign the informed consent form with your research doctor.

Thank you very much for reading this.

APPENDIX 2: SAMPLE CONSENT FORM

(To be adapted to national requirements and to be presented on local headed paper)

Consent Form

Centre Number: xxx
Trial Number: xxx

V3.0, November 19, 2010
[Replace by local date and version]

Acronym and title of study: NEAT 001/ANRS 143 trial
Clinical trials identifier:

1. I confirm that I have read and understand the information sheet dated .... (version ....) for the above study and have had the opportunity to ask questions.

2. I understand that my participation is voluntary and that I am free to withdraw at any time, without giving any reason, without my medical care or legal rights being affected.

3. I understand that sections of any of my medical notes may be looked at by responsible individuals involved in the running of the trial or from regulatory authorities where it is relevant to my taking part in research. I give permission for these individuals to have access to my records.

4. I agree that blood sample which will be drawn during the trial may be frozen and stored. These anonymised samples may be shipped from one country to another and used for further study of the HIV virus, the immune system and side effects of treatments. I understand that I have the right to demand the destruction of these samples if I wish so.

5. I accept that data collected for this study will be anonymised and recorded in an informatic database

6. I agree to take part in the NEAT 001/ANRS 143 study (“main trial”). □ yes □ no

7. I agree to take part in the “Dynamics” sub-study. □ yes □ no

8. I agree to take part in the Bone sub-study. □ yes □ no

9. I agree to take part in Neurocognitive function sub-study. □ yes □ no

Name of Patient	Date	Signature
Trial Physician:<br>Name, complete address and telephone number	Date	Signature

APPENDIX 3: SAMPLE PHARMACOGENETICS CONSENT

PHARMACOGENETIC BLOOD DNA SAMPLE
PATIENT INFORMATION AND CONSENT

Centre Number: xxx
Trial Number: xxx

V3.0, November 19, 2010
[Replace by local date and version]

Purpose The purpose of this information and consent form is to give you information so that you can decide whether you want to provide an additional blood sample and health information for pharmacogenetic and other related research.
Your participation in this pharmacogenetic research is voluntary. If you decide that you do not want to participate in the pharmacogenetic research you may still participate in the main clinical trial NEAT 001/ANRS 143. In connection with the pharmacogenetic research, you will also be asked to sign this separate form authorizing the use and disclosure of your identifiable health information for this additional study.

Introduction: Cells in the human body contain genes composed of deoxyribonucleic acid (DNA). The genes contain key instructions for cell function and help determine the characteristics of each individual. Pharmacogenetic research uses DNA samples from healthy and ill individuals to do the following:

- study the causes of human diseases
- help understand how different individuals respond to drugs
- obtain information to help develop new methods to diagnose and treat diseases

About This Research:
This research will help to understand how individuals with HIV respond to drug treatments and why they develop adverse events. The research may lead to the identification of additional genes associated with these medical conditions, which may eventually lead to new treatments.

Procedure: If you agree to participate, one blood sample (of approximately two teaspoons), will be drawn from your arm by study personnel. This sample is in addition to any blood samples that will be drawn for the purpose of your medical care or the main clinical trial.
The blood sample will be anonymised and stored. The anonymised sample may be shipped from one country to another for storage and analysis.

The DNA information obtained from your blood sample will be used along with the other information collected from the main clinical trial in which you are a participant, to study the differences in drug behavior that is generally seen in people with HIV infection.

The pharmacogenetic element to this study will be confined to researching the elements of this proposal. **You will not be contacted in connection with the research or any information about the results of genetic tests performed on the sample that you donate for this research.** It is possible that your participation may contribute to the knowledge of the causes of medical conditions, or may help in developing new drugs or methods to detect or treat disease.

By signing this consent form, you give NEAT 001/ANRS 143 study investigators permission to use your health information, your blood sample, and the DNA obtained from your sample for research.

Withdrawal of Consent and Destruction of Samples:
You may withdraw this consent and discontinue your participation in the pharmacogenetic research described above without affecting your participation in the main NEAT 001/ANRS 143 trial.

Confidentiality:
We are sensitive to the privacy risks associated with genetic research and we have procedures in place to safeguard your privacy and confidentiality. Confidentiality will be respected as described in the patient information for the main NEAT 001/ANRS trial (*insert version , date*).
Our pharmacogenetic research is not intended to provide you with clinical information. No genetic information will be returned to you or your health care provider. Information resulting from the research will not be entered into your medical records.
At some point in the future, information about the results of the studies may be published; however, you will not be identified in any such publication.

Development for Commercial Gain: The DNA obtained from your blood sample may be used for the development of new therapies, diagnostic methods, medicines, treatments for disease, information, materials and other developments which may be patented or otherwise have commercial value to the NEAT 001/ANRS 143 study investigators or other third parties. By consenting to participate in this research, you authorize the use of your sample for the research described above, and you acknowledge that there are no plans to provide financial benefits or compensation to you should this occur.

Questions/Information: If you have any questions regarding this sample collection or pharmacogenetic research or if you experience an injury caused by the sample collection procedure, you should contact your Trial Physician.

Consent:
I have read the preceding information describing this sample collection and all my questions regarding collection of my blood sample and medical/health information for pharmacogenetic and related research have been answered to my satisfaction. I understand that after agreeing to participate in this research, I may later decide to revoke this consent at any time.
I agree to provide a blood sample and to permit my medical and health information to be used and disclosed for pharmacogenetic and related research as described above. I understand that I will receive a signed copy of this consent form.

______________________	_______________	___________________
Name of Patient	Date	Signature
______________________	_______________	___________________
Trial Physician:<br>Name, complete address and telephone number	Date	Signature

APPENDIX 4: CDC CLASSIFICATION SYSTEM FOR HIV INFECTION (REVISION 1993)

Reference: 1993 revised classification system for HIV infection and expanded surveillance case definition for AIDS among adolescents and adults.
MMWR 1992; 41 (No. RR-17): 1-19.

The clinical categories of HIV infection are defined as follows:

Category A
Category A consists of one or more of the conditions listed below in an adolescent or adult (≥ 13 years) with documented HIV infection. Conditions listed in Categories B and C must not have occurred.

- Asymptomatic HIV infection
- Persistent generalised lymphadenopathy
- Acute (primary) HIV infection with accompanying illness or history of acute HIV infection

Category B **(Symptomatic non-AIDS conditions)**
Category B consists of symptomatic conditions in an HIV-infected adolescent or adult that are not included among conditions listed in clinical Category C and that meet at least one of the following criteria: a) the conditions are attributed to HIV infection or are indicative of a defect in cell-mediated immunity; or b) the conditions are considered by physicians to have a clinical course or to require management that is complicated by HIV infection. **Examples** of conditions in clinical category B include, **but are not limited to:**

- Bacillary angiomatosis
- Candidiasis, oropharyngeal (thrush)
- Candidiasis, vulvovaginal; persistent, frequent, or poorly responsive to therapy
- Cervical dysplasia (moderate or severe)/cervical carcinoma in situ
- Constitutional symptoms, such as fever (38.5 C) or diarrhoea lasting > 1 month
- Hairy leukoplakia, oral
- Herpes zoster (shingles), involving at least 2 distinct episodes or more than one dermatome
- Idiopathic thrombocytopenic purpura
- Listeriosis
- Pelvic inflammatory disease, particularly if complicated by tubo-ovarian abscess
- Peripheral neuropathy

For classification purposes, Category B conditions take precedence over those in Category A. For example, someone previously treated for oral or persistent vaginal candidiasis (and who has not developed a Category C disease) but who is now asymptomatic should be classified in clinical Category B.

Category C **(AIDS indicator conditions as defined by diagnostic or presumptive** measures)
Category C includes the clinical conditions listed in the AIDS surveillance case definition. For classification purposes, once a Category C condition has occurred, the person will remain in Category C.

Conditions in Category C include:
- Candidiasis of bronchi, trachea, or lungs
- Candidiasis, oesophageal
- Cervical cancer, invasive
- Coccidiomycosis, disseminated or extrapulmonary
- Cryptococcosis, extrapulmonary
- Cryptosporidiosis, chronic intestinal (> 1 month's duration)
- Cytomegalovirus disease (other than liver, spleen or nodes)
- Cytomegalovirus retinitis (with loss of vision)
- Encephalopathy, HIV-related
- Herpes simplex: chronic ulcer(s) (> 1 month's duration); or bronchitis, pneumonitis, or oesophagitis
- Histoplasmosis, disseminated or extrapulmonary
- Isosporiasis, chronic intestinal (> 1 month's duration)
- Kaposi's sarcoma
- Lymphoma, Burkitt's (or equivalent term)
- Lymphoma, immunoblastic (or equivalent term)
- Lymphoma, primary, or brain
- Mycobacterium avium complex or M. kansaii, disseminated or extrapulmonary
- Mycobacterium tuberculosis, any site (pulmonary or extrapulmonary)
- Mycobacterium, other species or unidentified species, disseminated or extrapulmonary
- Pneumocystis carinii pneumonia
- Pneumonia, recurrent
- Progressive multifocal leukoencephalopathy
- Salmonella septicaemia, recurrent
- Toxoplasmosis of brain
- Wasting syndrome due to HIV

The following list of other non-CDC HIV-associated conditions was compiled, as the CDC notes that Category B is not limited to events listed in the CDC 1993 definition.

- Aspergillosis
- Leishmaniasis

- Microsporidiosis
- Molluscum contagiosum
- Nocardiasis
- Thrombotic microangiopathy (hemolytic uremic syndrome [HUS]/ thrombotic thrombocytopenia purpura [TTP]

APPENDIX 5: SERIOUS NON-AIDS EVENTS

This appendix defines serious non-AIDS events to be captured in the primary endpoint.

Serious non-AIDS defining events captured in the primary endpoint

- Severe infections defined by one of the following conditions:

1. sepsis with bacteremia requiring overnight hospitalisation
2. pneumonia requiring ventilation or being associated with a low oxygen partial pressure (defined as an oxygenation below 85% measured by pulse oximenty)
3. Bacterial meningitis with either presence of bacteria on Gram strain and/or culture of CSF, either presence of > 500 leucocytes/mm3 in the CSF
4. Soft tissue or cutaneous infection, of presumed or proved bacterial etiology (treated with antibiotics), requiring overnight hospitalization.

- Liver cirrhosis Child-Pugh B or C
- Serious chronic renal disease: defined either by confirmed creatinine clearance < 15 ml/min, or initiation of dialysis, or chronic kidney disease requiring renal transplantation
- Serious cardiovascular events: myocardial infarction, coronary revascularisation, stroke, requiring or not hospitalisation.
- Any non-AIDS malignancies, except for basal cell carcinomas

Only new events are included in the analysis.
For patients with liver cirrhosis Child-Pugh B at inclusion, worsening to stage C qualifies for the endpoint.

Any serious non-AIDS event defined above should be notified to the regional CTU within 24 hours of becoming aware to the event (cf. protocol sections 8.2.3 and 8.3). To qualify for the primary endpoint, serious non-AIDS events need to be validated by the Endpoint Review Committee (ERC). The members of the ERC are unaware of the treatment assignment of the patient (blinded review).

APPENDIX 6: TABLE FOR GRADING ADVERSE EXPERIENCES

A.N.R.S

ANRS scale to grade the severity of adverse events in adults

Version n° 1.0   4 November 2008

(Translation of the French version n°6 9 September 2003*)*

This severity scale is a working guide intended to harmonise evaluation and grading practices for symptomatology in ANRS biomedical research protocols.

In practice, the items evaluated are grouped according to the system taking the form of a non-exhaustive symptomatic table (and not a classification of pathologies). Our choices focus on the most frequently observed clinical and biological signs or those whose monitoring is essential to ensure the protection of the subjects participating in the research.

For abnormalities NOT found elsewhere on the Table, refer to the scale below to estimate grade of severity:

**GRADE 1**	***Mild***	Mild or transient discomfort, without limitation of normal daily activities; no medical intervention or corrective treatment required.
**GRADE 2**	***Moderate***	Mild to moderate limitation of normal daily activities; minimal medical intervention or corrective treatment required.
**GRADE 3**	***Severe***	Marked limitation of normal daily activities; medical intervention and corrective treatment required, possible hospitalisation.
**GRADE 4**	***Life-threatening***	Severe limitation of normal daily activities; medical intervention and corrective treatment required, almost always in a hospital setting.

Abbreviations used in the table:

**ULN**	:	Upper Limit of Normal
**RBC**	:	Red Blood Cells
**FEV1**	:	Forced Expiratory Volume in one second
**EMG**	:	Electromyogram
**Prothrombin Time (%)**	:	Corresponds to Quick time (sec)
**aPTT**	:	activated Partial Thromboplastin Time

This document is the exclusive property of the ANRS and must not be modified or published without the authors' prior consent. Any use is subject to prior consent from the ANRS. Any questions should be addressed to pharmacovigilance@anrs.fr.

Please note that this scale was devised for use in HIV, HCV or HBV related pathologies.

ANRS scale to grade the severity of adverse events in adults (version no 1.0 4 November 2008)

	GRADES	GRADE 1<br>Mild	GRADE 2<br>Moderate	GRADE 3<br>Severe	GRADE 4<br>Life-threatening
			**HAEMATOLOGY**		
1	Haemoglobin (g/dl)	8.0 – 9.4	7.0 – 7.99	6.5 – 6.99	< 6.5
2	Leucocytes (/mm^3)	3 000 – 3 900	2 000 – 2 999	1 000 – 1 999	< 1 000
3	Neutrophils (/mm^3)	1 000 – 1 500	750 – 999	500 – 749	< 500
4	Platelets (/mm^3)	75 000 – 99 000	50 000 – 74 999	20 000 – 49 999	<20 000 or generalized petechiae
5	Prothrombin Time (%)	/	45 – ≤ 70	20 – < 45	< 20
6	aPTT	1.0 – 1.66 x ULN	> 1.66 – 2.33 x ULN	> 2.33 – 3.0 x ULN	> 3.0 x ULN
			**BIOCHEMISTRY**		
			Hepatic and pancreatic biochemistry		
7	AST (SGOT) (UI/l)	1.25 – 2.50 x ULN	> 2.50 – 5.0 x ULN	> 5.00 – 10.0 x ULN	> 10.0 x ULN
8	ALT (SGPT) (UI/l)	1.25 – 2.50 x ULN	> 2.50 – 5.0 x ULN	> 5.00 – 10.0 x ULN	> 10.0 x ULN
9	GAMMA GT (UI/l)	1.25 – 2.50 x ULN	> 2.50 – 5.0 x ULN	> 5.00 – 10.0 x ULN	> 10.0 x ULN
10	Alkaline phosphatase (UI/l)	1.25 – 2.50 x ULN	> 2.50 – 5.0 x ULN	> 5.00 – 10.0 x ULN	> 10.0 x ULN
11	Hyperbilirubinaemia (µmol/l)	1.25 – 2.50 x ULN	> 2.50 – 5.0 x ULN	> 5.00 – 10.0 x ULN	> 10.0x ULN
12	Amylasaemia (UI/l) / Lipasaemia (UI/l)/ Pancreatitis	≥1.25 – 2.50 x ULN	> 2.50 – 5.0 x ULN	> 3.0 x ULN with acute abdominal pain and/or imaging indicating acute pancreatitis.	> 3.0 x ULN with abdominal pain and signs of shock.
13	CPK (UI/l)	1.25 – 2.50 x ULN	> 2.50 – 5.0 x ULN	> 5.00 – 10.0 x ULN	> 10.0 x ULN
			Lipid status		
14	Hypertriglyceridaemia (mmol/l)	/	4.50 – 8.59	8.60 – 13.70	> 13.70
15	Hypercholesterolaemia (mmol/l)	>ULN –7.75	>7.75 – 10.34	>10.34 – 12.92	>12.92

ANRS scale to grade the severity of adverse events in adults (version no 1.0 4 November 2008)

	GRADES	GRADE 1<br>Mild	GRADE 2<br>Moderate	GRADE 3<br>Severe	GRADE 4<br>Life-threatening
			Electrolytes / Evaluation of renal function / Metabolism		
16	Hyponatraemia (mEq/l)	130 – 135	123 – 129	116 – 122	<116
17	Hypernatraemia (mEq/l)	146 – 150	151 – 157	158 – 165	>165
18	Hypokalaemia (mEq/l)	3.2 – 3.4	2.8 – 3.1	2.5 – 2.7	<2.5
19	Hyperkalaemia (mEq/l)	5.6 – 6.0	6.1 – 6.5	6.6 – 7.0	>7.0
20	Bicarbonate (mEq/l or mmol/l)	20.0 – 24.0	15.0 – 19.99	10.0 – 14.99	< 10.0
21	Creatininaemia (µmol/l)	1.0 – 1.50 x ULN	> 1.50 – 3.0 x ULN	> 3.0 – 6.0 x ULN	6.0 x ULN or dialysis required
22	Blood Urea Nitrogen (UI/l)	1.25 – 2.5 x ULN	2.6 – 5.0 x ULN	5.1 – 10 x ULN	> 10 x ULN
23	Hypocalcaemia (mmol/l)	1.95 – 2.10	1.75 – 1.94	1.50 – 1.74	< 1.50
24	Hypercalcaemia (mmol/l)	2.65 – 2.87	2.88 – 3.13	3.14 – 3.38	> 3.38
25	Hypophosphataemia (mg/dl)	2.0 – 2.4	1.5 – 1.9	1.0 – 1.4	<1.0
26	Hyperuricaemia (µmol/l)	1.25 – 2.0 x ULN	> 2.0 – 5.0 x ULN	> 5.0 – 10.0 x ULN	> 10.0 x ULN
27	Hypoglycaemia (mmol/l)	3.1 – 3.6	2.2 – 3.0	1.7 – 2.1	< 1.7
28	Hyperglycaemia (mmol/l)	6.1 – 7.0	> 7.0 – 16.5	> 16.5 without ketosis.	See diabetes<br>Item no. 52 (grade 4)
29	Hyperlactataemia (mmol/l)<br>(venous blood sample)	2.0 – 2.99*	3.0 – 3.99**	4.0 – 4.99**	≥ 5.0***
			Urinalysis		
30	Proteinuria (dipstick)	+	++	≥ +++	Nephrotic syndrome
31	Haematuria.	≥ 80 RBC/µl<br>(dipstick).	≥ 200 RBC/µl<br>(dipstick).	Macroscopic with or without clots.	Obstructive or requiring a blood transfusion.

* *Lactataemia – GRADE 1: a confirmatory test is necessary within 8 to 10 days*
** *Lactataemia – GRADE 2, 3: a confirmatory test is necessary within 24 hours.*
*** *Lactataemia – GRADE 4: a confirmation test is necessary immediately.*

ANRS scale to grade the severity of adverse events in adults (version no 1.0 4 November 2008)

GRADES		GRADE 1<br>Mild	GRADE 2<br>Moderate	GRADE 3<br>Severe	GRADE 4<br>Life-threatening
			Gastro-intestinal/hepatic/pancreatic abnormalities		
32	Nausea.	Transient,<br>normal diet.	Restricted diet for less than 3 days.	Restricted diet for more than 3 days.	Liquid only diet.<br>Hospitalization required.
33	Vomiting.	Transient:<br>2 – 3 episodes / day or duration ≤ 1 week.	Repeated:<br>4 – 5 episodes / day or duration > 1 week.	Solid/liquid vomiting for 24 h.<br>Orthostatic hypotension.<br>Perfusion required.	Hospitalization for hypovolemic shock.
34	Diarrhoea.	Transient,<br>3 – 4 stools / day,<br>diarrhoea ≤ 1 week.	Persistent,<br>5-7 stools / day,<br>diarrhoea > 1 week.	> 7 stools/day or requiring perfusion.<br>Bloody stools.	Hospitalization,<br>Hypovolemic shock,<br>perfusion.
35	Constipation.	/	Moderate abdominal pain,<br>78 h without stools.<br>Treatment required.	Meteorism.<br>Requiring disimpaction or hospital treatment.	Meteorism with vomiting or occlusion.
36	Dysphagia.	Mild discomfort when swallowing.	Difficulty in swallowing but food intake possible.	Inability to swallow solids.	Inability to swallow liquids, perfusion required.
37	Oesophagitis.	Pyrosis occurring less than once a week	Pyrosis occurring at least once a week but relieved by PPIs*	Pyrosis occurring at least once a week but not relieved by PPIs*	Food intolerance and vomiting

**PPIs: proton pump inhibitors*

ANRS scale to grade the severity of adverse events in adults (version no 1.0 4 November 2008)

	GRADES	GRADE 1<br>Mild	GRADE 2<br>Moderate	GRADE 3<br>Severe	GRADE 4<br>Life-threatening
			Respiratory abnormalities		
38	Bronchospasm.	Transient,<br>no treatment,<br>FEV1 70 % - < 80 %.	Permanent,<br>Improvement under bronchodilation<br>FEV1 50 % - < 70 %.	Persistent under bronchodilation.<br>FEV1 25 % - < 50 %.	Cyanosis,<br>FEV1 < 25 %<br>intubation.
39	Dyspnoea	Dyspnoea upon exertion.	Dyspnoea during normal daily activities.	Dyspnoea at rest.	Dyspnoea requiring respiratory assistance.
			Muscular abnormalities		
40	Myalgia<br>(excluding injection site).	Mild myalgia for less than 4 weeks. Not requiring analgesic treatment.	*Presence of one of the following symptoms:*<br>1 – Mild to moderate myalgia for more than 4 weeks and/or which may require treatment with level 1* analgesics.<br>2 – Predominance of difficulties upon exertion (difficulty in climbing stairs or rising from a sitting position). Can walk without assistance.<br>Optional confirmation through the identification of biological (CPK), electromyographical (EMG) or histological (muscular biopsy) abnormalities.	*Presence of one of the following symptoms*:<br>1 – Moderate to severe myalgia for more than 4 weeks requiring treatment with level I/II* analgesics.<br>2 – Assistance required for walking and normal daily activities.<br>Paraclinical confirmation recommended (CPK, EMG and/or muscular biopsy).	*Presence of one of the following symptoms*:<br>1 – Severe myalgia not related to exertion requiring treatment with level II/III* analgesics.<br>2 – Muscular weakness making walking impossible without assistance.<br>3 – Acute rhabdomyolysis with muscular necrosis and oedema.<br>4 – Acute rhabdomyolysis with electrolytic disturbances and renal insufficiency.<br>Paraclinical confirmation required<br>(biology, EMG and/or muscular biopsy).

** Level I analgesics : Peripheral analgesics (paracetamol and/or salicylics or non-steroid anti-inflammatory drugs) ;*
** Level II analgesics : Weak opiates (codeine, dextropropoxyphene), morphinic agonists-antagonists (buprenorphine, nalbuphine) ;*
** Level III analgesics : Morphine.*

ANRS scale to grade the severity of adverse events in adults (version no 1.0 4 November 2008)

	GRADES	GRADE 1<br>Mild	GRADE 2<br>Moderate	GRADE 3<br>Severe	GRADE 4<br>Life-threatening
			Cardiovascular abnormalities		
41	Arterial hypertension.	Transient or permanent.<br>Increased blood pressure ≤ 20 mmHg and systolic BP 140-159 or diastolic BP 90-99.	Permanent.<br>Increased blood pressure > 20 mmHg and systolic BP 160-179 or diastolic BP 100-109.	Permanent.<br>Systolic BP ≥ 180 or diastolic BP > 110	Malignant or accelerated arterial hypertension.
42	Orthostatic hypotension.	Decreased systolic blood pressure ≤ 20 mmHg in orthostatic position. No treatment.	Decreased systolic blood pressure > 20 mmHg, durable but corrected with liquid intake per os.	Perfusion required.	Hypovolemic shock requiring hospitalization.
43	Ventricular cardiac rhythm disorders.	/	Isolated ventricular extrasystoles, no treatment, symptomatic or asymptomatic.	Recurrent, persistent or symptomatic cardiac rhythm disorders. Treatment required.	Dysrhythmia requiring hospitalization.
44	Prolongation of the QT interval.	/	Man: >450 and < 500 ms<br>Woman: >470 and <500 ms	>500ms	>500 ms with clinical symptoms (ventricular rhythm disorders, syncope, torsade de pointes)
45	Cardiac ischaemia.	/	Atypical pain under exploration.	Appearance of angina upon exertion, controlled with treatment.	Myocardial infarction, unstable angina, preinfarction syndrome.
46	Pericarditis.	Chance discovery of a small effusion during ultrasound scan	Moderate effusion with few symptoms. No treatment or intervention deemed necessary for the time being.	Moderate or significant symptomatic effusion but without tamponade. Treatment required and hospitalization to be considered.	Tamponade.<br>Hospitalization and intervention required.
47	Stroke.	/	/	Transient Ischemic Attack<br>(regressive focal neurological syndrome within 24 h).	Cerebrovascular accident non-regressive within 24 h.
48	Peripheral arterial embolism.	/	/	/	Peripheral arterial embolism.<br>Hospitalization.<br>Adapted treatment.
49	Deep vein thrombosis and/or pulmonary embolism.	/	/	Deep vein thrombosis.<br>Anticoagulant treatment.<br>Hospitalization to be considered.	Pulmonary embolism.<br>Adequate hospitalization and treatment.

ANRS scale to grade the severity of adverse events in adults (version no 1.0 4 November 2008)

	GRADES	GRADE 1<br>Mild	GRADE 2<br>Moderate	GRADE 3<br>Severe	GRADE 4<br>Life-threatening
	***Endocrine* abnormalities**				
50	Hyperthyroidism.	Infraclinical hyperthyroidism. Low TSH. Normal free T3 and T4.	Moderate, non-complicated thyrotoxicosis.<br>Treatment required.	Malignant exophtalmia.<br>Cardiac arrhythmia.<br>Myopathy.	Thyrotoxic crisis and/or cardiac insufficiency.
51	Hypothyroidism.	Infraclinical hypothyroidism. Increased TSH but <12 mU/l. Normal free T4.	Simple hypothyroidism without complications. Treatment required.	Severe hypothyroidism with multiple clinical symptoms.<br>Urgent treatment.<br>Hospitalization to be considered.	Myxoedematous coma.
52	Diabetes/hyperglycaemia.	Moderate fasting hyperglycaemia between 6.1 and 7 mmol/l. No immediate treatment required.	Fasting glycaemia: > 7 mmol/l. Special diet required, possibly supplemented with oral antidiabetics.	Fasting glycaemia:>16.5 mmol/l on an empty stomach, with or without clinical symptoms. Insulin therapy required.	Ketoacidosis or hyperosmolarity (>27.8 mmol/l without acidosis).
	***Cutaneous* abnormalities**				
53	Cutaneous and/or mucosal eruptions.	Erythaema,<br>Moderate pruritis.	Extended maculopapular eruption, with or without pruritis.	Extended papulovesicular or oozing eruption.<br>Palpable purpura (suggestive of vasculitis).<br>Polymorphous erythaema.<br>Small-size cutaneous or mucous ulcerations.	Any blistering cutaneous and/or mucosal lesions (Lyell or Stevens-Johnson).<br>Febrile erythrodermia, whether or not associated with other signs indicative of hypersensitivity.<br>Cutaneous necrosis requiring surgical excision.
54	Symptoms of immediate hypersensitivity, with or without cutaneous symptoms.	/	Acute localised urticaria.	Giant urticaria,<br>Quincke's oedema.	Anaphylactic shock.

ANRS scale to grade the severity of adverse events in adults *(version no 1.0 4 November 2008)*

GRADES		GRADE 1 Mild	GRADE 2 Moderate	GRADE 3 Severe	GRADE 4 Life-threatening
Neurological abnormalities					
55	Wakefulness / sleep disorders.	Minor attention and concentration impairment.	Diurnal somnolence and/or difficulty falling asleep and/or night time awakening, mental activity decreased, obnubilation.	Sleep-wake cycle modification or insomnia requiring treatment or change in dream content. Obvious confusional syndrome with temporal disorientation.	Sleep-wake cycle disorganisation not responding to treatment. Dreamlike confusional syndrome, coma and/or convulsion.
56	Psychiatric disorders.	Minor anxiety.	Anxiety requiring treatment or moderate depression.	Major anxiety or confirmed depressive episode requiring treatment.	Acute psychosis requiring hospitalization, including suicidal ideation, manic state, hallucinatory delusion.
57	Cephalalgia.	Intermittent, no treatment.	Requiring level I* analgesics.	Requiring at least level II* analgesics.	Not responsive to level III* analgesics.
58	Paraesthesia.	Paraesthesia, mild pain, no treatment.	Paresthesia, permanent pain of moderate intensity, requiring level I* analgesics.	Paraesthesia, permanent pain of severe intensity, requiring at least level II* analgesics.	Unbearable pain resulting in disability, restricted activity despite administration of level III* analgesics.
59	Motor deficiency.	Subjective feeling of weakness without objective impairment, no reflex changes.	Distal motor deficiency, moderate functional impairment or reflex changes.	Marked motor deficiency interfering with normal daily activities.	Confined to bed or a wheelchair because of motor deficiency.
60	Difficulty controlling movement.	Occasional clumsiness, mild coordination difficulties.	Tremor or dyskinaesia or dysmetria, or dysarthria, moderate limitation of normal daily activities.	Upper or lower limbs ataxia or abnormal movements, limitation of normal daily activities.	Inability to stand up. Total dependence.
61	Sensory loss.	Mild sensory loss, regardless of mode and distribution (focal or symmetric).	Moderate sensory loss.	Severe sensory loss.	Extensive sensory loss involving the trunk and four limbs.

** Level I analgesics : Peripheral analgesics (paracetamol and/or salicylics or non-steroid anti-inflammatory drugs) ;*
** Level II analgesics : Weak opiates (codeine, dextropropoxyphene), morphinic agonists-antagonists (buprenorphine, nalbuphine) ;*
** Level III analgesics : Morphine.*

ANRS scale to grade the severity of adverse events in adults (version no 1.0 4 November 2008)

GRADES		GRADE 1<br>Mild	GRADE 2<br>Moderate	GRADE 3<br>Severe	GRADE 4<br>Life-threatening
Miscellaneous					
62	Fever (oral temperature, °C) for more than 12 h.	37.7 – 38.9	39 – 39.5	39.6 – 40.5	> 40.5
63	Renal colic.	Spontaneous regression of symptoms.<br>Pain not requiring treatment.	Colic requiring medical treatment.	Obstructive syndrome, does not disappear spontaneously.	/
64	Fatigue.	Normal daily activities reduced by less than 25% for less than 48 h.	Normal daily activities reduced by 25 – 50 % for more than 48 h.	Normal daily activities reduced by more than 50%, cannot work for more than 48 h.	Unable to care for self. Assistance required for normal daily activities.
65	Arthritis / Arthralgia.	Arthralgia.	Arthralgia, with or without articular effusion or with moderate functional impairment.	Marked arthritis with or without effusion or with severe functional impairment.	/
66	Ocular disorders.	Conjunctival hyperaemia.	Moderate pain.<br>Conjunctivitis.	Decreased visual acuity.<br>Uveitis.<br>Severe pain. Glaucoma.	/

APPENDIX 7: ESTIMATION OF CREATININE CLEARANCE (COCKCROFT AND GAULT EQUATION)

In men:

Creatinine clearance (ml/min) = 1.23 x (140-age) x weight / creatininemia

In women:

Creatinine clearance (ml/min) = 1.04 x (140-age) x weight / creatininemia

With: **age** in years, **weight** in kg, **creatininemia** in µmol/L.

Cockcroft D W & Gault M H. Prediction of creatinine clearance from serum creatinine. Nephron 1976 ;16:31-41

APPENDIX 8: BIOBANK PROCEDURES AND ASSESSMENTS

Biobank sampling in all patients

Plasma and full blood samples for storage are drawn at all study visits, except the screening visit. Urine samples are stored at visits W00, W04, W12, W48 and W96.

The amount of blood to be drawn and the measurements scheduled on the biobank samples in all patients are listed in the table “biobank 1” on the next page. Other immunologic or virologic measurements, e.g. additional genotypes, proviral DNA and circular DNA or other, may be performed.

The handling and centralisation of biobank samples are described in a separate document (trial specific SOP).

Biobank sampling for sub-studies

Additional samples for storage are scheduled for patients participating in the following sub-studies: Bone sub-study; Viral and Immunologic Dynamics and Inflammation sub-study (see table “biobank 2”). Please refer to the sub-study protocols for details.

Biobank Table 1: Biobank sampling and planned assessments in all patients

Visit	W-6	W00	W02	W04	W08	W12	W18	W24	W32	W48	W64	W80	W96	FU-M
**Week**	**-6 to -1**	**0**	**2**	**4**	**8**	**12**	**18**	**24**	**32**	**48**	**64**	**80**	**96**	**Every 3 to 4 months**
**Day**	-42 to -7	0	14±2	28±2	56±7	84±7	126± 7	168± 7	224± 7	336± 7	448± 15	560± 15	672± 15	
**Biobank blood samples to be drawn:**														
- EDTA tubes (7 ml)		3	3	5	3	3	3	5	3	3	3	3	3	X[2]
- EDTA tubes (1.5 ml)[1]		1												
**Biobank urine sample**		x		x		x				x			x	
**To be stored as:**														
- Plasma aliquots (1 ml)		5	4	8	4	4	4	8	4[2]	4[2]	4[2]	4[2]	4[2]	X[2]
- Full blood aliquots (1 ml)		4	4	4	4	4	4	4	4	4	4	4	4	
- Full blood aliquot (1.5 ml)[1]		1												
- Urine aliquots (1 ml)		4		4		4				4			4	
**Planned measurements**														
- Plasmatic ARV concentrations				X				X						
- Drug disposition genes[1]		X												
- Genotype		X												
- Kidney function markers		X		X		X				X			X	

1 Only in patients having signed the separate pharmacogenomics informed consent. If not performed at W00, this can be performed at any point during the trial.

2 One additional aliquot to be stored In case of HIV-1 RNA ≥ 500 copies/ml after 32 weeks.

Biobank Table 2: Overview of additional sampling for storage and planned assessments in sub-studies

Visit	W-6	W00	W01	W02	W03	W04	W08	W12	W18	W24	W32	W48	W64	W80	W96
**Week**	**-6 to -1**	0	1	2	3	4	8	12	18	24	32	48	64	80	96
**Day**	-42 to -7	0	7±2	14±2	21±2	28±2	56±7	84±7	126± 7	168± 7	224± 7	336± 7	448± 15	560± 15	672± 15
**Additional sampling in sub-study participants**[1]															
Bone sub-study:<br>- Serum biobank<br>- Urine biobank		5 ml<br>5 ml										5 ml<br>5 ml			5 ml<br>5 ml
Viral and Immunologic Dynamics and Inflammation sub-study:<br>- Full blood (ACD or heparin tube)[2]		50 ml	5 ml	5 ml	5 ml	30 ml	30 ml	30 ml		50 ml		50 ml			50 ml

1 Please refer to sub-study protocols for details
2 Samples to be shipped immediately at room temperature to closest core immunology lab (see appendix 15)

APPENDIX 9: PHARMACOGENETICS AND POPULATION PHARMACOKINETICS

TITLE

Relationship between plasma drug concentrations (pharmacokinetics, PK), virological response (pharmacodynamics, PD), and drug disposition genes (pharmacogenetics, PGx) - Protocol summary

AUTHORS

Marta Boffito

DOCUMENT VERSION AND DATE

V1.0, October 21, 2009

RATIONALE

Many factors influence the effects of medications, including age, organ function, concomitant therapy, drug interactions, and the nature of the disease. Moreover, there are now numerous examples of cases in which inter individual differences in drug response are due to sequence variants in genes encoding drug-metabolising enzymes, drug transporters, or drug targets. It is well recognised that different patients respond in different ways to the same medication.

Despite huge advances in terms of the impact of antiretroviral therapy on HIV-related morbidity and mortality patients continue to fail therapy.

Most first-line regimens for the treatment of HIV are now similar in potency and, assuming susceptibility to the selected regimen determined by genotypic resistance testing, tolerability and toxicity are the key issues differentiating currently available agents.

Importantly, drug concentrations and drug disposition gene patterns may explain virological failure or development of toxicity in HIV-infected patients. Previous studies have shown that approximately 30% of patients have low drug concentrations and these have shown to be associated to virological failure [Alexander et al. Antiretroviral concentrations in untimed plasma samples predict therapy outcome in a population with advanced disease. J Infect Dis 2003;188:541-8].

This ancillary study allows for identifying what subgroup of patients fails therapy, or develops toxicity, for pharmacological reasons.

The relation between drug concentrations and adherence is investigated in order to assess the percentage of patients failing therapy, or developing toxicity, because of drug plasma exposure.

OBJECTIVES

- To investigate the relationship between drug plasma exposure (PK) and drug disposition genes (PGx)
- To investigate the relationship between drug plasma exposure (PK) and virological response (PD, achieving and maintaining of undetectable viral load)

ENDPOINTS

- PK/PGx relationship for raltegravir, darunavir/r, and tenofovir
- PK/PD (in terms of efficacy and toxicity) relationship for raltegravir, darunavir/r, and tenofovir

SUB-STUDY COMPONENTS AND PROCEDURES

- PGx written consent form: PGx written consent from is to be signed at baseline (W00) or at any later visit during the study if more convenient for the Investigator or the study participant.
- The following blood samples are scheduled for the (1) PGx and (2) PK/PD substudy:
    1. One EDTA (1.5 ml) bottle for storage at any time during the trial (after signature of pharmacogenomics consent). This sample is to be frozen in one aliquot of 1.5 ml full blood and shipped at -80°C to a centralised laboratory for the determination of drug disposition genes (PGx).
    2. 10 ml in EDTA bottles and preparation of 4 aliquots of 1 ml plasma for storage at W04 and W24. These samples are to be stored at -80°C and may be used for the later determination of drug plasma concentrations.

       Important:

       A) 2. must be spun and plasma isolated and frozen at -80°C until shipment (4 aliquots of 1 ml of plasma).

       B) Blood samples for the determination of plasma concentrations must be taken ≥ 5 hours post-dose in order to be able to project trough concentrations

       C) Time of previous dose and blood sampling must be reported

- All blood samples aimed at PGx investigations must be anonymised.

Analysis is coupled with adherence assessments

SAMPLE SIZE:
whole 800 patient population (PGx analysis must not be performed when PGx separate written consent is not obtained)

PARTICIPATING CENTRES: All

APPENDIX 10: VIRAL SUB-TYPE ANALYSIS

TITLE
HIV-1 subtype determinants of outcome

AUTHORS
Deenan Pillay, Carlo Federico Perno, Vincent Calvez, Charles Boucher

DOCUMENT VERSION AND DATE
V2.0, November 19, 2010

RATIONALE
HIV-1 group M is divided into multiple genetic forms, termed subtypes, or clades. Through founder effects, these subtypes have become established in geographically defined areas of the world, and within specific risk groups. The established North American and European epidemics have been characterized by subtype B virus. Thus, the vast bulk of data on responses to therapy and emergence of resistance within randomised studies is from subtype B virus. One major advantage of the NEAT European network is to allow questions to be asked on comparative ART data from an array of subtypes. Through inclusion of Eastern European countries, and infected individuals who have migrated to Europe from Africa and Asia, we are uniquely positioned to address viral subtype issues.

There are a number of reasons why NEAT 001/ANRS 143 is appropriate to subtype analysis

a) the natural variation of HIV-1 integrase between subtypes identifies some minor resistance mutations (identified in subtype B) which are present as normal polymorphisms in other subtypes. It remains unclear whether these differences impact on clinical response to raltegravir.

b) Some workers have suggested that the K65R mutation in HIV-1 reverse transcriptase more commonly arises in subtype C than other subtypes. It follows that the genetic barrier may be lower for emergence of resistance in this subtype. Since this is best studied in treatment lacking ZDV (which antagonizes the emergence of K65R), it is particularly relevant to the TDF containing arms in NEAT 001/ANRS 143.

c) The current darunavir resistance algorithm includes a number of mutations (albeit minor mutations) which are present as polymorphisms in non- subtype B viruses. Whether their presence in drug naïve individuals indicates a lower threshold for viral rebound and emergence of additional resistance mutations is unknown.

d) It is recognized that gag mutations may impact on PI susceptibility, with or without corresponding protease mutations. The interaction of gag and protease is inextricably linked, and subtype-specific polymorphisms in gag may alter the genetic path to PI resistance

These questions are important for establishing an optimal evidence base to guide management of the heterogeneous patient population within Europe.

OBJECTIVES
- To compare responses to each arm of the study, across subtypes
- To assess whether subtype is an independent variable in determining response to therapy (in view of the overlap between subtype and risk group/ethnicity, input from the data analysis group is required for this)
- To determine whether subtype specific patterns of emerging resistance to each of the two arms can be discerned, by comparison with baseline HIV-1 sequence

- To identify baseline mutational associations with failure of specific regimens (e.g. integrase for RAL, protease for DRV and RT for tenofovir)
- To verify the phenotype of novel mutations/sets of mutations observed within the trial, by mutagenesis of subtype specific HIV-1 vector systems (in contrast to the standard use of subtype B viruses for commercial phenotypic investigations). This includes exploring gag-protease interrelationships for those failing PI therapy.

ENDPOINTS

- Determination of time to treatment failure, overall, as well as by arm, by subtype of virus
- Description of mutational changes associated with resistance
- Identification of mutations predictors of resistance. For instance, integrase mutations in case of RAL treated individuals

STUDY COMPONENTS AND PROCEDURES

Samples

All patients recruited to this sub study.

1. Screening: Genotype is assessed in the local laboratory (if not previously available).
2. W00 sample – 10 ml plasma for HIV-1 RNA. An aliquot of at least 1 ml is stored frozen and stored (biobank). It may later be sent to a central laboratory responsible for additional genotype testing or if no FASTA file is available in the case of historical genotype testing.
3. At first HIV-1 RNA ≥ 500 copies/ml after 32 weeks: HIV-1 genotype is assessed in the local laboratory. In addition, 1 ml of plasma is stored and frozen (biobank). It may later be sent to a central laboratory responsible for additional genotype testing.

Sequencing

Reverse transcriptase and protease genes are sequenced in the local laboratories at screening and time of virological rebound. Additional sequencing (e.g. integrase, complete gag gene) may be performed by centralised laboratories using biobank samples. Nucleotide sequences are entered into the central NEAT Virology Group database. Interpretation is undertaken centrally using Virology Sub group algorithm.

Phenotype

In selected cases, where a unique set of resistance mutations are observed:

a) to insert specific mutations emerging in those failing therapy into subtype specific HIV-1 vectors, to assess the drug susceptibility implications
b) to identify potential gag mutations, within individuals subtypes

Analysis

Subtype is determined on all screening sequences by use of the on line Rega algorithm (http://jose.med.kuleuven.be/subtypetool/html/index.html)

APPENDIX 11: RENAL FUNCTION STUDY

TITLE
Renal function study

AUTHORS
Jose R Arribas, Hansjakob Furrer, Christoph Fux, Peter Reiss, Jose Ignacio Bernardino, Stephan de Wit, Christine Katlama

DOCUMENT VERSION AND DATE
V1.0, October 21, 2009

RATIONALE
Multiple reports have indicated that the antiretroviral drug tenofovir has renal toxicity. Secondary analyses from clinical trials comparing antiretroviral regimens suggest that the risk of tenofovir toxicity is low (1%-2%). In contrast, in the Johns Hopkins cohort, use of tenofovir was associated with a greater decline in renal function than was use of alternative nucleoside analogue. The relative decline in creatinine clearance (CL_{Cr}) associated with the use of tenofovir, compared with that associated with the use of alternative nucleosides, was 4%.
In addition, tenofovir administration has been associated with Fanconi's syndrome, which is characterised by hypophosphatemia, hyperphosphaturia, glycosuria, and aminoaciduria; skeletal complications of this syndrome include osteomalacia.
The long-term impact of tenofovir on renal function is still unknown.

Two regimens are to be compared in NEAT 001/ANRS 143:
- **darunavir/ritonavir + tenofovir/ emtricitabine**
- **darunavir/ritonavir + raltegravir**

NEAT 001/ANRS 143 thus offers a good opportunity to compare the nephrotoxicity risk of a tenofovir containing regimen with a tenofovir-sparing regimen.

Calculated creatinine clearance is a useful, and probably the only feasible, measurement to assess glomerular function.
Relevant proximal tubular dysfunction is possible without a relevant reduction in glomerular filtration.
There are no established diagnostic criteria for renal tubular function, and even the Fanconi Syndrome lacks a quantitative definition. Moreover, tubular dysfunction is a continuum of mild to severe losses of function.
Measuring tubular function with isolated serum and urine markers is insufficient, because these markers have a very low specificity. By contrast, fractional excretion equations for phosphate and uric acid, as well as normoglycemic glucosuria, are considered specific for proximal tubular function and can be easily and cheaply measured. As a clearcut definition of proximal tubular dysfunction is not available, fasting urine and serum samples are stored at the important timepoints to be able to measure more expensive markers in selected patients.

OBJECTIVES
- To compare changes in renal function between the two treatments strategies
- To compare proximal renal tubular function between the two treatment strategies

ENDPOINTS

- Proportion of patients with reduced glomerular function at W48 and W96, defined as at least 25% reduction in calculated GFR (sustained in two measurements at least one month apart)
- Proportion of patients with pathological Proximal Renal Tubule Dysfunction (PRTD) at W48 and W96, defined as one of the following:
  a) fractional excretion of phosphate > 20% (>10% in patients with concurrent hypophosphatemia) or
  b) a score of at least 3 out of the 4 following:
    - pathological fractional excretion of uric acid
    - euglycemic glucosuria
    - pathological protein/creatinine ratio in urine
    - low serum bicarbonate

- Change from baseline in glomerular filtration rate as measured by Cockroft-Gault formula at W48 and W96
- Change from baseline in glomerular filtration rate as measured by MDRD formula at W48 and W96
- Cystatin-C serum levels at W48 and W96
- Protein:Creatinine ratios (Urine) at W48 and W96

STUDY COMPONENTS AND PROCEDURES

Planned assessments:

- **Serum**: Potassium, phosphate, creatinine, alkaline phosphatase, gamma-glutamyltransferase and alanine aminotransferase, uric acid, bicarbonate, sample for freezing.
- **Urine spot**: Creatinine, protein, phosphate, uric acid, glucose; dip stick test for leukocytes, erythrocytes, proteinuria and glucosuria, pH; sample for freezing.

Timepoints:

W00, W04, W12, W48, W96 in fasting state

SAMPLE SIZE:

whole 800 patient population

PARTICIPATING CENTRES: All

APPENDIX 12: QUALITY OF LIFE ASSESSMENTS

TITLE
Health-related quality of life in HIV-infected people

AUTHORS
Rita Murri, Raffaella Bucciardini, Pythia Nieuwkerk, Bruno Spire, Antonio Morselli, Damiano Abeni, Vincenzo Fragola, Francesca Borghetti, Marco Lauriola, Maurizio Massella, Nikos Dedes, Alain Volny-Anne, Brian West

DOCUMENT VERSION AND DATE
V1.0, October 21, 2009

RATIONALE
Guidelines recommendations for treatment in HIV infection are generally based on the information obtained in clinical trials. Traditionally, this has been mostly represented by immunological and virological response and by a safety profile defined through the adverse events occurring during the study. Such measures, however, provide only an incomplete assessment of the composite effects of treatment, and lack information that comes directly from the patient. Therefore, patient reported outcomes (PRO) now play an important role both in research and clinical practice in the evaluation of patients' health status. Health-related quality of life (HRQoL), a complex PRO referring to all the aspects of a person's well-being, has recently been recognised by guidelines as a main therapeutic objective and is therefore increasingly used in the comparison of anti-HIV drugs and as an additional or independent measure of drug evaluation.

To date, suppression of HIV is possible in the vast majority of patients with a wide variety of regimens. However, each of these effective HAART regimens may differ in their associated limitations, such as differences in side effect profiles and differences in convenience of regimens. This leads to questions such as how regimens with comparable efficacy but different side effect profiles should be weighed against each other, and how potency should be weighed against tolerability and convenience. Therefore, it is investigated whether specific benefits and limitations associated with each of the treatments arms of the NEAT 001/ANRS 143 trial translate into differences in patients' health-related quality of life.

OBJECTIVE
- To compare during follow up the effects of the two different regimens on health related quality of life (HRQoL), self-reported symptoms, treatment satisfaction, and depression.

ENDPOINTS
To compare the two different regimens in terms of:
- change of quality of life from baseline (even considering strata according to different levels of CD4, HIV RNA, symptom score)
- time to change of quality of life score (EQ-5D and list of patients reported outcomes)
- changes in QoL measures are compared with efficacy data (responses in terms of CD4 and HIV-RNA, clinical progression – AIDS and non-AIDS related events and death -), assessing possible correlations.
- changes in QoL measures are compared with safety data (toxicity, grade 3-4 or any grade), assessing possible correlations.
- changes in symptom patient-reported score are compared with safety data (toxicity, grade 3-4 or any grade), assessing possible correlations.

- changes in QoL measures are compared with adherence data
- differences in QoL changes between patient with HIV/HCV coinfection and patient with HIV infection
- differences in QoL changes between patient with lipodystrophy and patient without lipodystrophy

SUB-STUDY COMPONENTS AND PROCEDURES

Instruments of the study are:
a) A generic HRQol questionnaire: EQ-5D (it is a brief measure of generic HRQL with established reliability and validity. The EQ-5D is available in many languages and can also be used to calculate quality adjusted life years).
b) HIV specific symptom scale (22 among the most experienced symptoms are listed with 5 possible options ranging from not at all to very much; a total score is calculated);
c) CES-D for the assessment of depression (a short version of 10 items is used);
d) HIVTSQs for the treatment satisfaction assessment (The trial treatment arms differ with respect to the number of daily doses, with one treatment arm having once daily dosing and the other treatment arm having twice daily dosing. Therefore, it is relevant to supplement the HRQL assessment with a measure of regimen convenience and treatment satisfaction).

The QoL assessment are done on the whole population (n=800) at W00, W04, W12, W24, W48, and W96.

No blood sampling is planned.

Analysis is centralised.
Data on HRQoL is matched with pharmacoeconomic data as well as with data on adherence.

APPENDIX 13: ADHERENCE STUDY

SUB-STUDY TITLE

AUTHORS
Andrea Antinori

DOCUMENT VERSION AND DATE
V2.0, November 19, 2010

RATIONALE
The inclusion of sub-studies concerning the issues of pharmacology and medication adherence into the NEAT 001/ANRS 143 trial is highly advisable, mainly because of the potential impact of both these variables on the primary study end-point of treatment efficacy. Many studies have investigated the role of adherence to antiretrovirals as well as of plasma drug concentrations on virological response, however no data is available for more recent antiretroviral drugs and new classes, such as those investigated in the NEAT 001/ANRS 143 trial. Further, because regimen complexity may represent a crucial barrier to optimal adherence, the possibility to control for medication adherence in the statistical analyses might be important, especially in the case of treatment arms dissimilar in terms of daily doses or pill burden.
Another focus of interest is that, at virological failure, adherence-resistance relationships vary according to the different antiretroviral classes and to drug-boosting. This is the result of the complex interplay between the capacity of resistant virus to replicate at different levels of drug exposure and its genetic barrier. In this context, the study of adherence and pharmacokinetic/pharmacodynamic characteristics of new drugs, such as RAL and DRV, would be valuable to describe treatment-specific adherence-resistance relationships.
Finally, the assessment of adherence to innovative treatment regimen could be essential to better understand its association with both toxicity profiles or quality of life-related issues investigated in other NEAT 001/ANRS 143 sub-studies.

OBJECTIVES
Primary objective

- To compare the extent of adherence across the two different first-line treatment strategies in HIV-1 infected antiretroviral naïve adults over 96 weeks: darunavir/r + tenofovir/emtricitabine vs. darunavir/r + raltegravir.

Secondary objectives

To compare the two different regimens in terms of:

- level of adherence required for successful virologic suppression
- relative prognostic value of adherence and plasma drug concentrations on the virological success
- relative prognostic value of adherence and plasma drug concentrations on the risk of resistance mutations to one or more drugs in case of virological failure
- relationship between adherence and drug-specific toxicities
- relationship between adherence and patient-reported quality of life.

ADHERENCE ASSESSMENTS

- All patients included in the NEAT 001/ANRS 143 trial are systematically evaluated for their adherence behaviour by using:
  - Previously validated patient-administered questionnaire to investigate self-reported adherence in terms of number of omitted doses/pills; number of days of complete adherence to trial regimen; overall estimation of adherence at VAS; timing deviation; drug holidays (modified version of the ACTG questionnaire) at W04, W12, W24, W48, and W96.
  - Pill count

At the start of the study, each subject should receive counselling regarding the importance of dosing adherence to antiretroviral medications. In order to document adherence with the treatment regimen, subjects should be instructed to bring their empty medication bottles to investigators at each study visit and their unused medications bottles at the end of the trial. An exact start and stop date, and the number of tablets dispensed and returned are recorded in the CRF. Pill counts are calculated as the number of pills taken (the number of pills dispensed – the number of pills counted). The number of pills expected to have been taken are calculated by multiplying the daily dose (1/2, 1 or 2 tablets) by the number of days since the date dispensed.
Moreover, at each study visit, patients are asked to complete a self-reported questionnaire (a partially modified ACTG questionnaire) investigating several non-adherence behaviours: overall estimated adherence using Visual Analogue Scale, number of missed doses over the last 4 days, number of days of complete adherence to trial regimen, timing deviation, and unplanned treatment interruption.

DERIVED ADHERENCE MEASURES

- From patient-reported assessment:
  - Percentage of doses taken/prescribed
  - Percentage of days of complete adherence to trial regimen
  - Percentage at VAS
- From pill count:
  - Percentage of pills returned/delivered

Sample size of the adherence study:
all patients

APPENDIX 14: PHARMACOECONOMICS STUDY

SUB-STUDY TITLE
Pharmacoeconomics in HIV-infected people

AUTHORS
Andrea Tramarin, Raffaella Bucciardini, Rita Murri, Pythia Nieuwkerk, Bruno Spire, Antonio Morselli, Damiano Abeni, Vincenzo Fragola, Francesca Borghetti, Marco Lauriola, Maurizio Massella, Nikos Dedes, Alain Volny-Anne, Brian West

DOCUMENT VERSION AND DATE
V2.0, March 1, 2010

RATIONALE
The introduction of HAART has resulted in significantly higher costs for therapeutics, while reductions have been observed in healthcare resource use due to the prevention of opportunistic infections (Gebo et al, 1999). Since the mid-1990s, this has resulted in a shift from inpatient towards outpatient care (Tramarin et al, 2004, Hubben et al, 2008). Given the fact that HIV prevalence rates are increasing due to falling death rates (for instance more than 56000 prevalent cases were estimated in Italy at the end of 2005 – Suligoi et al, 2006), the impact of HIV on national health budgets can be expected to increase (UNAIDS, 2007; Yazdanpanah et al., 2002).
According to an Italian recent study, the societal cost (direct, indirect, intangible) of HIV/AIDS (Hubben et al, 2008), is substantial, related to the treatment regimen followed, to the HAART success or failure, highly influenced by the indirect costs (loss of productivity).
The two alternative treatment strategies of the trial are compared in terms of cost-consequence: specifically the difference in the relevant costs and effects (see below for details), between the comparative options, are computed. The following possible scenarios mayl be identified:
1. The new/innovative option is both more effective and more costly than the reference option. In this case, the ratio of the difference of costs with the difference of effects is computed to estimated the Incremental Cost Effectiveness Ratio (ICER), i.e. the amount of incremental costs necessary to gain one unit of effects, for the more effective treatment option.
2. The new/innovative option is more effective and also less costly. In this case this treatment option is recognised to be dominant, as contemporarily more effective and less costly

OBJECTIVE
- To compare the two different regimens in terms of: 1) cost-effectiveness and 2) cost-utility in a subset of patients (Italy)
- To describe in-patient care resource utilisation in all patients

ENDPOINTS
- cost-effectiveness
- cost-utility

SUB-STUDY COMPONENTS AND PROCEDURES

- Data regarding in-patient care resource utilisation is recorded in all patients from W00 on.
- A detailed pharmacoeconomics substudy is conducted in Italy only:
- For patients participating in the trial in Italy, economic data are collected at W00, W24, W48, W64 and W96.
- Perspective of the analysis: the perspective of the main payer(s) is adopted (National Health Service or other third party payers or patients; country specific issue). Costs and effects to be included in the analyses: both direct costs (for instance for drug therapy, for hospitalisation) and effects (expressed in clinical terms, as from the main study) and intangible costs and effects (i.e. impairment/improvements of HRQoL) are analysed.
- Technique: Cost-Effectiveness and Cost-Utility Analysis is conducted in order to estimate the incremental cost per physical/clinical effectiveness and/or per Quality Adjusted Life Years (QALY) gained. To estimate the QALY the utility is estimated from the health states reported with the EQ-5D and the social tariffs estimated and approved within the EuroQoL group.
- Time horizon: the time horizon adopted for this study is the same as for the core trial: accordingly costs and effects occurring after the first year of observation are discounted.
- Effects on health related quality of life, expressed in terms of utility, obtained from the assessment with the EQ-5D questionnaire (quality of life assessment) are also evaluated.

APPENDIX 15: VIRAL AND IMMUNOLOGIC DYNAMICS AND INFLAMMATION SUB-STUDY

TITLE
Viral and immunologic dynamics and inflammation

AUTHORS
Brigitte Autran, Guido Poli, Reinhold Schmidt, Deenan Pillay, José Arribas, Peter Reiss, Christine Katlama

DOCUMENT VERSION AND DATE
V4.0, November 19, 2010

RATIONALE
Apoptotic loss of uninfected T cells appears to be a consequence of the generalised chronic high levels of immune activation that accompany pathogenic HIV infection. This concept is supported by observations that T cell activation is a good predictor of disease progression in untreated HIV-infected individuals, and that CD4 T cell depletion correlates more closely with levels of immune activation than with viral load. Thus, immune activation is considered to be a major factor contributing to HIV-related quantitative and functional immune deficiencies and CD4 defects as well as to HIV-related cardiovascular alterations. It is supposed to be linked to HIV both directly through activation of immune competent cells releasing pro-inflammatory cytokines or adhesion molecules and indirectly through tissue (mainly gut) associated damages.
Some cellular markers of activation such as CD38 and HLA-DR on CD8 T cells have been clearly defined as the best markers of immune activation. The T cell immune activation is also detectable on CD4 T cells and is known to favour HIV entry and integration partly by enhancing cell surface expression of CCR5, the main HIV entry co-receptor, in parallel to HLA-DR. In both cases the classical monitoring using the enumeration of percentages of CD4 or CD8 T cells expressing those markers can be improved by using a direct quantification of the CD38 or CCR5 molecules, thus allowing a highly sensitive measurement of immune activation and its consequences for HIV entry.
Many seric markers of immune activation have also been proposed such as pro-inflammatory cytokines released by the activated immune competent cells, among which IL-6 appears as the best marker, or adhesion molecules shed from the endothelial surface, among which V-CAM appears as the best marker.

NEAT-001/ANRS 143 is a 2 year randomised open-label, multinational, multicentre trial in HIV-infected naïve patients comparing two regimens of antiretroviral therapy including a combination of 2 potent new ARV drugs (boosted-darunavir + raltegravir) and a darunavir/ritonavir containing triple therapy (darunavir/r + tenofovir/emtricitabine). The new raltegravir containing drug combination is supposed to induce a very rapid, profound and sustained reduction in viral load. However, these observations require confirmation. Furthermore, the mechanism of such a reduction in potency requires study. For instance, it remains unclear whether any increased potency defined as more rapid decline of viral load is reflected in a reduction in the circulating reservoir, or the intracellular state of the viral genome. These parameters can be determined by quantifying episomal versus integrated proviral DNA, and also studying the nature of viral mRNA produced through transcription. Increased potency of the drug regimen would also be accompanied by a reduction in immune activation and a rapid, potent and sustained immune restoration. This supposed profound reduction in immune activation requires highly sensitive and reliable methods of

monitoring immune activation. Furthermore immune activation, endothelial damage, vascular dysfunction, activity of proinflammatory cytokines, hypercoagulable state and dyslipidemia are different mechanisms that could increase HIV-related cardiovascular risks. Therefore it is still an open scientific question if different types of antiretrovirals are associated with different metabolic and/or proinflammatory changes that could impact in different ways on the cardiovascular risk of HIV-infected patients. The NEAT 001/ANRS 143 trial thus provides a unique opportunity to evaluate within the network of high tech immunology laboratories linked to NEAT, the ability of those new drug combinations at better or more sustainably reducing immune activation than more conventional regimens using highly sensitive markers of immune activation and therefore at allowing a better efficacy and tolerability of these drug regimens.

OBJECTIVES

To study the correlation between viral decay and immunologic parameters

To compare between the two treatment strategies:
- the early immunologic dynamics in relationship with the viral dynamics
- the efficacy to
    - reduce the immune activation over 96 weeks as assessed by the numbers of CD38 molecules expressed by CD8 T cells and their co-expression with HLA-DR on CD8 T cells
    - reduce the CD4 cell expression of CCR5, the main HIV co-receptor of entry, the expression of which is associated to immune activation as assessed by the quantification of CCR5 molecules on CD4 T cells and their co-expression with HLA-DR
    - reduce plasma levels of seric markers of immune activation linked to inflammation and cardiovascular alterations (among those: IL-6, V-CAM…)
    - induce immune restoration of naïve and memory T cells numbers and functional competence against pathogens and HIV
- lipid changes
- glucose metabolism changes
- endothelial damage
- change in cardiovascular risk
- change in proinflammatory cytokines
- change in coagulation parameters

To identify the best surrogate of viral suppression in the two treatment arms

To study the association between residual viral replication, proviral DNA, immune activation, endothelial activation, and inflammation

ELIGIBILITY CRITERIA

Inclusion Criteria
- Patients having signed the consent form for the NEAT 001/ANRS 143 trial and having been successfully screened for the trial
- Patients followed in a centre participating in the Viral and immunologic dynamics and inflammation sub-study
- Signed consent for the sub-study

Non-inclusion Criteria
- Hemoglobin ≤ 10 g/dl (assessed during the last 3 months prior to screening visit)

If hemoglobin level falls ≤ 10 g/dl between W00 and W02, sampling for this sub-study should be suspended until hemoglobin is > 10 g/dl.

ENDPOINTS

- First-and second-phase decay rates of plasma HIV-1 estimated by a biexponential viral-dynamics model
- Change in Plasma HIV RNA, CD4 cell count and proportion of patients with viral suppression at time points defined in the table below
- Percentage and number of CD3+CD8+CD38+HLADR+ (at time points defined in the table below)
- CCR5 and DR expression on CD4
- Plasma levels of IL-6, V-CAM
- Percentage and number of naïve and memory cells
- CD4 and CD8 response (SFC)
- Change from W00 to W48, and W96 in:
  - Fasting total cholesterol, LDL cholesterol, HDL cholesterol and triglycerides, apo-A1, apo-B, lipoprotein profiling.
  - Fasting glucose and insulin.
  - Coagulation Markers

SUB-STUDY COMPONENTS AND PROCEDURES

The assessments evaluate in the two different treatment strategies (see table 1, page 136).

- CD4 count at W00, day 2-3, W01, W02, W04, W08, W12, W18, W24, W32, W48, W64, W80, W96
- immunologic markers W00, W01, W02, W03, W04, W08, W12, W24, W48, W96:
  - level of expression of CCR5 on CD4+ T cells in association to immune activation as assessed by the quantification of CCR5 molecules on CD4 T cells in relationship with the level of CD38 expression on CD8 T cells and the proportions of activated HLA-DR+ CD4+ and CD8 T cells, and the proportions of naïve and memory CD4 T cells
  - numbers and functional competence of T cells against pathogens and HIV
- markers of endothelial activation and inflammation at W00, W02, W04, W08, W12, W24, W48, W96 (among those: Ultra-sensitive C-reactive protein, IL-6, V-CAM, serum amyloid A, serum amyloid P etc)
- coagulation markers: D-dimer, PAI-1, prothrombin fragment 1+2 at W00, W48, W96
- intensive lipids: total cholesterol, triglycerides, HDL-cholesterol; LDL-cholesterol, apo-A1, apo-B, lipoprotein profiling by NMR spectroscopy at W00, W04, W48, W96
- intensive glucose: fasting glucose and insulin, urine glucose (dipstick), fasting proinsulin, fasting C-peptide, HOMA at W00, W04, W48, W96
- plasma HIV-1 RNA at W0, day 2-3, W01, W02, W03, W04, W08, W12, W18, W24, W32, W48, W64, W80, W96
- total proviral DNA, episomal and integrated DNA, and spliced/unspliced RNA at W00, W01, W02, W03, W04, W08, W12, W24, W48, W96
- residual viral replication (HIV-1 RNA < 1 copy/ml) at W24, W48, W96

Patients participating in this sub-study undergo additional blood sampling at W00, day 2-3, W01, W02, W03, W04, W08, W12, W24, W48, and W96 (see table 2, page 137). This

includes three extra visits to the study site (day 2-3, W01, W03) in addition to the core protocol visits.

This study requires core laboratories of immunology/virology with high tech performances in flow cytometry (these core laboratories could be in France, Italy, Germany, UK, Spain) and molecular virology, and local laboratories of immunology with very good performances in viable cell cryopreservation and reliable capacities of plasma storage.
Blood samples for analysis of immunologic and virologic markers need to be sent at room temperature and arrive at the closest core immunology lab during working hours and no later than 12h after sampling (see table 2, page 137).

In addition, this study requires expert laboratories to determine markers of endothelial activation and inflammation. These markers are measured on biobank samples of the core trial.

Sample size:
40 patients per arm
For viral dynamics, studies of optimal designs have shown that the scheduled measurements and number of subjects would be enough for 80% power for comparison of viral decays between treatment arms (Retout et al. Stat Med 2007; 26:5162-79). Assuming 10% of loss-to-follow-up and 25% of treatment interruption at W96, the inclusion of 80 patients allows for the evaluation of 60 patients still on treatment at the end of follow-up. This sample size (60) would be enough to show a difference of at least 0.5 log10 copies/ml in HIV RNA decline and a difference of at least 80 CD4+ cells/µL with a statistical power of 80%, assuming a common standard deviation of 0.67 log10 copies/ml and 102 cells/µL, respectively and using a Wilcoxon test.

Coparticipation
Patients participating in this sub-study should be encouraged to participate also in the bone substudy or the neurocognitive function sub-study.

Table 1: Viral and immunologic dynamics and inflammation sub-study: Overview of planned assessments

**Visit**	**W -6**	**W 00**	**Day 2-3**	**W 01**	**W 02**	**W 03**	**W 04**	**W 08**	**W 12**	**W 18**	**W 24**	**W 32**	**W 48**	**W 64**	**W 80**	**W 96**
**Study procedures**																
Informed consent	X	(X)														
**Local lab assessments**																
Plasma HIV-1 RNA[1]		(X)	X	X	X	X	(X)	(X)	(X)	(X)	(X)	(X)	(X)	(X)	(X)	(X)
CD4 cell count[1]		(X)	X	X	X		(X)	(X)	(X)	(X)	(X)	(X)	(X)	(X)	(X)	(X)
**Planned centralised assessments**																
Residual viral replication											X		X			X
Proviral DNA, episomal and integrated DNA, spliced/ unspliced RNA		X		X	X	X	X	X	X		X		X			X
PBMC activation		X			X		X	X	X		X					X
Immunologic markers (PBMC and serum)		X		X	X	X	X	X	X		X		X			X
Endothelial activation and inflammation		X			X		X	X	X		X		X			X
Intensive lipids		X					X						X			X
Intensive glucose		X					X						X			X
Coagulation Markers		X											X			X

1 Assessments in parentheses (X) are performed in the core trial and do not need to be programmed specifically for the sub-study.

Table 2: Viral and immunologic dynamics and inflammation sub-study: Specific laboratory assessments in addition to core protocol sampling, sampling volumes and sample circuit

Visit	W00	Day 2-3	W01	W02	W03	W04	W08	W12	W18	W24	W32	W48	W64	W80	W96
**Local lab**															
Plasma HIV-1 RNA		X	X	X	X										
CD4 cell count		X	X	X											
**Sampling for immediate shipment to core immunology lab**[1]	X		X	X	X	X	X	X		X		X			X
Total amount blood sample (ACD or heparin tubes) for centralisation	50 mL		5 mL	5 mL	5 mL	30 mL	30 mL	30 mL		50 mL		50 mL			50 mL
Temperature of shipment	RT[2]		RT[2]	RT[2]	RT[2]	RT[2]	RT[2]	RT[2]		RT[2]		RT[2]			RT[2]

1 Samples must arrive at the core immunology laboratory during working hours and no later than 12h after sampling.
2 RT = room temperature

APPENDIX 16: BONE SUB-STUDY

SUB-STUDY TITLE
NEAT 001/ANRS 143 "Bone Mineral Density and Bone Turnover Substudy"

AUTHORS
Jose R Arribas, Peter Reiss, Patrick Mallon, Jose Ignacio Bernardino, Stephan de Wit, Christine Katlama

DOCUMENT VERSION AND DATE
V3.0, June 22, 2010

RATIONALE
Although prevalence estimates of low bone mineral density (BMD) vary considerably, most studies indicate increased prevalence in HIV-infected individuals. The prevalence of osteopenia and osteoporosis may be affected by ART regimen, associated body composition changes, duration of HIV infection, chronic illness, and other concomitant risk factors for bone loss. Although initial reports indicated that HIV-infected patients exposed to a PI-containing regimen had a higher prevalence of reduced bone mineral density, this has not been consistently observed in subsequent cross-sectional and longitudinal studies The clinical relevance of the high prevalence of osteopenia is important. A recent study has shown a higher prevalence of bone fractures in HIV-infected patients (Triant et al J Clin Endoc Metab 2008) than in HIV negative controls.

Initiation of ART is associated with early reductions in BMD. Initiation of tenofovir- and stavudine-containing ART regimens has been shown to lead to initial, modest bone loss which was significantly greater at 48 weeks in patients randomised to receive tenofovir, but that subsequently stabilises (Gallant JE et al, JAMA. 2004;292:191-201). As both treated groups experienced loss of BMD, it is unclear if the observed losses arise as a direct result of ART toxicity or if they arise secondary to changes arising as a result of treated HIV infection, such as restoration of body fat mass, which in animal studies has been shown to affect BMD.

Very recently, the SMART trial has provided evidence supporting that ART use contributes to the decline in BMD. Continuous ART may increase the risk of fractures relative to intermittent, CD4-guided ART. In this study stavudine, zidovudine and lopinavir/r were associated with decline in spine BMD by quantitative computer tomography (qCT) and dual X-ray absorptiometry (DXA). No evidence was found for association of tenofovir with BMD loss, contrary to other studies. Since the sample size for this substudy was relatively small, these results should be interpreted with caution.

Comparing a tenofovir-containing regimen with a nucleoside/tenofovir sparing regimen in antiretroviral naïve patients in the NEAT001/ANRS 143 trial helps to determine the role of tenofovir in any early loss of bone mineral density and to identify if all patients experience early loss in BMD or if greater losses are experienced by those randomised to NRTI/tenofovir-containing ART. This is an important goal given the ageing HIV-infected population, the increased fracture risk in patients with HIV and the increased fracture risk associated with low BMD.

OBJECTIVES
- To compare changes in bone mineral density between the two treatments strategies
- To compare markers of bone metabolism between groups

- To determine the relationships between changes in BMD and changes in body composition

ELIGIBILITY CRITERIA

Participating sites should propose participation in the bone sub-study systematically to all patients at screening.

Inclusion Criteria
- Patients having signed the consent form for the NEAT 001/ANRS 143 trial and having been successfully screened for the trial
- Patients followed in a centre participating in the bone sub-study
- Signed consent for the bone sub-study

Non-inclusion Criteria
- Peripheral oedema or prosthetic limb.
- Taking systemic corticosteroids or oestrogen replacement therapy at the time of screening (oestrogen intake at a dosage for oral contraception is not a non-inclusion criterion)
- Ongoing anti-osteoporosis treatment at screening: biphosphonates, teriparatide, strontium ranelate, raloxifen, calcitonin

ENDPOINTS

- Primary endpoint: Mean % change of BMD in lumbar spine and hip from baseline estimated by DXA scan at week 48.
- Secondary endpoints:
  - Mean % change of BMD in lumbar spine and hip from baseline estimated by DXA scan at week 96
  - Time weighted AUC percentage change from baseline in BMD at week 96
  - Proportion of patients meeting World Health Organization criteria for Osteoporosis (T-score<-2.5 standard deviation) at week 48 and 96
  - Proportion of patients meeting World Health Organization criteria for Osteopenia (T-score between-1 and-2.5 standard deviation) at week 48 and 96
  - Incidence of fractures
  - Association between baseline FRAX® score and incident fractures over 96 weeks of the study.
  - Proportion of patients with vitamin D deficiency
  - Proportion of patients with secondary hyperparathyroidism
  - Change in total and regional body fat at week 48 and 96

SUB-STUDY COMPONENTS AND PROCEDURES

Sub study-specific clinical history:
- Fracture history (including low-impact fractures)
- Family history of fractures
- Lifetime exposure to corticosteroids or recreational steroid use, heparin, oestrogen replacement therapies, vitamin D supplementation and opiates
- Estimates of physical activity
- History of depression
- Last menstrual period (female subjects only, to estimate onset of menopause / menarche)
- History of chronic inflammatory diseases
- History of renal failure
- Alcohol use: Non drinker. Ex-drinker. Current drinker: number of drinks per day.

DXA at W00, W48 and W96:

- Lumbar spine
- Proximal femur
- Total and regional (limb) body fat

The authorised visit windows for the DXA assessment at the visits are as follows:
W-4 to W08 for first DXA; W42 to W54 for second DXA; W80 to W96 for third DXA.
Should a woman become pregnant during her participation in the bone sub-study, no DXA measurement must be performed during pregnancy. Nevertheless, all other relevant data for the sub-study should be collected and follow-up in the sub-study should be continued during and after pregnancy.

Collection and storage of adequate blood and urine samples for full bone metabolism study at W00, W48, W96:

- Additional blood samples for biobank: 2 serum collection vials (7 ml per vial)
  - preparation of 5 aliquots of 1 ml of serum for storage
- Additional urine sample:
  - preparation of 5 aliquots of 1 ml urine for storage

The following assessment are planned on the stored samples:

- Serum and urinary calcium
- Serum intact PTH
- Serum vitamin D [25(OH)D and 1,25 (OH)D], fibroblast growth factor-23 (FGF-23).
- Serum bone formation markers (serum bone alkaline phosphatase, osteocalcin, serum procollagen type 1 amino-terminal propeptide (P1NP), serum procollagen type 1 carboxi-terminal propeptide (P1CP), serum osteoprotegerin, tartrate-resistant acid phosphatase 5 (TRAP-5)
- Serum bone resorption markers (serum skeletal acid phosphatase, serum c-telopeptide crosslinks (CTX), serum RANKL (receptor activator of nuclear factor κB ligand)
- Urinary bone resorption markers: hydroxyproline pyridinoline (Pyr), urinary DPD (deoxypyridinoline crosslinks), urine peptide-bound N-telopeptide crosslinks (NTX)
- Inflammatory markers (TNF-alpha, IL1, IL6)
- Markers of adipose tissue metabolism (leptin, adiponectin)

Patients have to be strictly fasting for the blood and urine sampling (nil by mouth). Urine samples should be collected on the 2nd morning urine.
Samples need to be stored at – 80° C until analysis.

RADIATION EXPOSURE

The radiation dose involved (0.0003mGy for total body scan and 0.037mGy for hip and spine scans [GE Lunar Prodigy Brochure, 2004]) is well below the yearly recommended radiation exposure.

SAMPLE SIZE

64 patients per arm would provide 80% power to detect a between treatment difference of 2.5% in BMD in lumbar spine assuming a standard deviation of 5 using a 2 sided t-test with alpha of 0.05. Assuming a 10% drop out rate a total of 142 patients would be included.

CENTRALISED ANALYSIS

Central Lab with prior expertise in performing biochemical markers of bone turnover

DATA ANALYSIS:

BMD:

- Between group absolute and percentage change form baseline in BMD at both hip and spine are estimated for all sub study participants at each of the pre-specified time points. Time weighted AUC percentage change in BMD is also estimated.
- Baseline demographic, treatment-related factors and changes in total or regional body fat associated with change in BMD both in the cohort as a whole and within randomised treatment groups are determined.
- An interim analysis is performed once the last enrolled patient has completed the week 48 assessments.

Biomarkers:

- If significant changes in BMD are observed in this study, further associations between changes in parameters of interest (including changes in hormonal measures and biomarker measures of bone metabolism from stored samples) and changes in BMD are to be examined. Models to investigate potential pathogenic pathways involved in any observed changes in BMD may be constructed.

Participating countries/centres:

Sites should have the correct equipment for bone densitometry by DXA and preferably have already used it in the context of multicenter trials. It is expected that 25-30 sites would participate in this substudy.

Coparticipation

These patients should be encouraged to also participate in the Viral and immunological dynamics and Inflammation sub-study.

APPENDIX 17: NEUROCOGNITIVE FUNCTION SUB-STUDY

SUB-STUDY TITLE
NEAT 001/ANRS 143 Substudy "Neurocognitive functions in HIV-infected patients"

AUTHORS
Hélène Amieva, Andrea Antinori, Valerio Tozzi, Alan Winston

DOCUMENT VERSION AND DATE
V2.0, November 19, 2010

RATIONALE
The introduction of antiretroviral therapies has substantially reduced the incidence of HIV-associated dementia. Nevertheless, milder patterns of cognitive impairment remain relatively frequent among patients with advanced HIV infection. According to studies, the prevalence of mild neurocognitive disorders varies from 20% to 40% of patients with HIV. Deficits generally affect memory, attention, psychomotor speed and for some patients result in difficulties in dealing with complex activities of daily living such as driving or managing domestic finances. Such cognitive difficulties have also been associated with diminished adherence to treatment. Regarding the effects of antiretroviral drugs on neurocognitive deficits, it is generally admitted that drugs have rather positive impact. Nonetheless, the benefits reported are considerably variable within studies. One of the reasons is that the effects are different according to the cognitive domains considered. Several studies have shown that antiretroviral drugs reduce HIV-related psychomotor slowing while the effect on memory has been shown to be more limited. In addition, the effects of antiretroviral drugs on cognition depend on their capacity to penetrate the central nervous system. Some antiretroviral drugs have indeed been identified as being more neurologically active on the basis of their capacity to reduce cerebrospinal HIV viral load to an undetectable level.
Regarding NEAT 001/ANRS 143 drug regimens, a more rapid decline of plasmatic viral load is expected after darunavir/r + raltegravir regimen initiation compared to darunavir/r + tenofovir/emtricitabine regimen. Even if the relationship between viral load and cognitive impairment is still largely debated, a difference in scores of cognitive functioning (in particular psychomotor speed) between the two patients groups can be hypothesized due to the differences in viral responses dynamics expected. Taking advantage of the co-participation of some of the patients enrolled in this substudy and in the "viral and immunologic dynamics and inflammation" substudy, the relationships between cognitive deficits and viral responses dynamics will be particularly investigated.

OBJECTIVES
Primary objective
- To compare the efficacy of the two NEAT 001/ANRS 143 treatment strategies on psychomotor speed

Secondary objectives:
- To compare the impact of the two NEAT 001/ANRS 143 treatment strategies on other cognitive, professional and functional activity measures

- To investigate the association between viral load, immune activation and neurocognitive function

ELIGIBILITY CRITERIA

Inclusion Criteria

- Patients having signed the consent form for the NEAT 001/ANRS 143 trial and having been successfully screened for the trial.
- Patient followed in a centre participating in the neurocognitive sub-study
- Signed consent for the neurocognitive sub-study

Non-inclusion Criteria

- Active drugs users (occasional/recreational drug users may be included)
- Patients with linguistic difficulties (according to clinician's appraisal)
- Patients with current or past opportunistic infections or tumors of the CNS, non HIV-related major neurological or psychiatric disorders

ENDPOINTS

Primary endpoint:

- Differences in scores measuring psychomotor speed

Secondary endpoints:

- Differences in the other attention and memory tests and questionnaires assessing subjective cognitive complaints, occupational attainment and functional abilities (abilities in complex activities of daily living)

SUB-STUDY COMPONENTS AND PROCEDURES

This sub-study is conducted at certain trial centres only. Eligible centres are those able to propose a full neuropsychological assessment of the patients performed by a psychologist or a study nurse who has received a brief training to the tests, scales and questionnaires selected for the purpose of the present sub-study. Eligible centres are also those able to use validated translated local language versions of the tests selected for this study.

At visit W00 and visit W96, HIV-infected patients enrolled into NEAT001/ANRS 143 trial in the eligible centres are proposed to undergo a neuropsychological assessment. Every effort should be made to perform the first neurocognitive assessment during the screening period, and if not possible then as soon as possible after inclusion and no later than W8 visit. The final neurocognitive assessment should be performed between W80 and W96 (ideally at W96).

The assessment is conducted as follows:

The patients are asked questions about their level of education and about their current occupational attainment. A short questionnaire assesses patient's own perception of cognitive abilities. Difficulties in coping with complex activities of daily living are investigated with Lawton's questionnaire of Instrumental Activities of Daily Living. In addition, a short neuropsychological battery is administered comprising the following tests:

- The Trail making test (parts A and B) assessing attention and mental flexibility

- The Digit symbol substitution task assessing psycho-motor speed

- The backwards digit span task assessing working memory
- The Free and cued selective reminding test assessing encoding and retrieval abilities in episodic memory
- Semantic and formal fluency tasks assessing verbal fluency
- The Frontal Assessment Battery assesing frontal / executive functions

In addition to these tests, the Lafayette Grooved Pegboard (dominant hand) test will be proposed to participants in italian centers.

The assessment takes about 50 minutes and is conducted by a trained psychologist or study nurse.
In addition to this assessment a short self-report questionnaire on recreational drug use will be given to the patient.

Sample size
The data used to estimate sample size are gathered from the "CogLoc study", an ongoing study conducted in a subsample of the "Aquitaine cohort" where about 400 patients with HIV are being assessed and followed-up with an extensive battery of cognitive tests.
In this sample, the mean score on the Digit Symbol Substitution task measuring psychomotor speed is 44.5 (SD: 12.9). A difference in the Digit Symbol Substitution task score of 10% (4-point difference) would be of clinical significance. Then, a sample of 182 subjects per arm would be required to detect with 90% power a difference in Digit Symbol Substitution task score of 10% between the two groups with a significance level of 5%.
Therefore, due to possible study dropouts, a sample size of 400 patients (200 per arm) is required.

APPENDIX 18: WORKING GROUPS

Virology working group:
Deenan Pillay (coordinator), Charles Boucher, Vincent Calvez, Carlo Federico Perno, Simon Collins

Pharmacology and adherence working group:
Marta Boffito (coordinator), Andrea Antinori

Immunology working group:
Brigitte Autran (coordinator), Reinhold Ernst Schmidt, Michal Odermarsky

Toxicity, including co-infection working group:
Jose Arribas (coordinator), Christine Katlama, Peter Reiss, Stephane De Wit, Xavier Franquet, Antonella Castagna

Quality of life and pharmaco-economics working group:
Rita Murri, Raffaella Bucciardini (coordinators of the Quality of Life study), Andrea Tramarin (coordinator of the pharmaco-economics study), Pythia Nieuwkerk, Brian West, Alain Volny-Anne

Neurocognitive function working group:
Hélène Amieva (coordinator), Valerio Tozzi, Alan Winston

APPENDIX 19: TRIAL MANAGEMENT

N°	COUNTRY	NATIONAL COORDINATING INVESTIGATOR	NEAT REGIONAL CTU
1	ITALY	Andrea Antinori	MRC, London
2	FRANCE	Christine Katlama	U897, Bordeaux
3	GERMANY	Georg Behrens	CHIP, Copenhagen
4	UNITED KINGDOM	Alan Winston	MRC, London
5	THE NETHERLANDS	Jan Prins	AIGHD, Amsterdam
6	IRELAND	Patrick Mallon	MRC, London
7	PORTUGAL	Francisco Antunes	CHIP, Copenhagen
8	SPAIN	Juan Gonzalez-Garcia	U897, Bordeaux, in collaboration with Gesida
9	BELGIUM	Stéphane de Wit	AIGHD/CHIP
10	POLAND	Andrzej Horban	CHIP, Copenhagen
11	HUNGARY	Denes Banhegyi	AIGHD, Amsterdam
12	SWEDEN	Anders Thalme	CHIP, Copenhagen
13	DENMARK	Jan Gerstoft	CHIP, Copenhagen
14	AUSTRIA	Brigitte Schmied	CHIP, Copenhagen
15	GREECE	Angelos Hatzakis	MRC, London

APPENDIX 20: INSURANCE POLICY

2

Copy of insurance policy to be inserted by the legal sponsor
